# Synthesis, spectroscopic characterization and thermogravimetric analysis of two series of substituted (metallo)tetraphenylporphyrins

**DOI:** 10.3762/bjnano.8.121

**Published:** 2017-06-02

**Authors:** Rasha K Al-Shewiki, Carola Mende, Roy Buschbeck, Pablo F Siles, Oliver G Schmidt, Tobias Rüffer, Heinrich Lang

**Affiliations:** 1 Inorganic Chemistry, Institute of Chemistry, Faculty of Natural Sciences, TU Chemnitz, 09107 Chemnitz, Germany; 2 Material Systems for Nanoelectronics, TU Chemnitz, 09107 Chemnitz, Germany; 3 Institute for Integrative Nanosciences, IFW Dresden, Helmholtzstrasse 20, 01069 Dresden, Germany

**Keywords:** electrospray ionization mass spectrometry, IR spectroscopy, metalloporphyrin, porphyrin, thermogravimetry, UV–vis spectroscopy

## Abstract

Subsequent treatment of H_2_TPP(CO_2_H)_4_ (tetra(*p*-carboxylic acid phenyl)porphyrin, **1**) with an excess of oxalyl chloride and HNR_2_ afforded H_2_TPP(C(O)NR_2_)_4_ (R = Me, **2**; iPr, **3**) with yields exceeding 80%. The porphyrins **2** and **3** could be converted to the corresponding metalloporphyrins MTPP(C(O)NR_2_)_4_ (R = Me/iPr for M = Zn (**2a**, **3a**); Cu (**2b**, **3b**); Ni (**2c**, **3c**); Co (**2d**, **3d**)) by the addition of 3 equiv of anhydrous MCl_2_ (M = Zn, Cu, Ni, Co) to dimethylformamide solutions of **2** and **3** at elevated temperatures. Metalloporphyrins **2a**–**d** and **3a**–**d** were obtained in yields exceeding 60% and have been, as well as **2** and **3**, characterized by elemental analysis, electrospray ionization mass spectrometry (ESIMS) and IR and UV–vis spectroscopy. Porphyrins **2**, **2a**–**d** and **3**, **3a**–**d** are not suitable for organic molecular beam deposition (OMBD), which is attributed to their comparatively low thermal stability as determined by thermogravimetric analysis (TG) of selected representatives.

## Introduction

**Figure 1:**
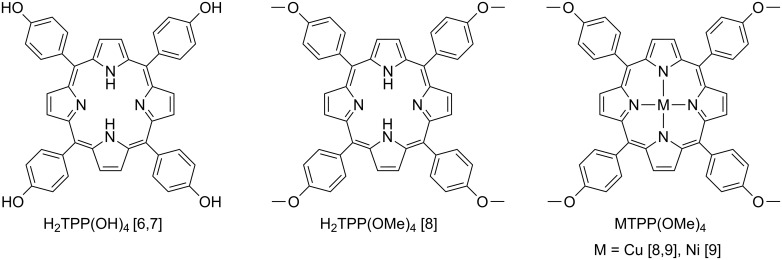
Chemical structures of porphyrins and metalloporphyrins successfully deposited by organic molecular beam deposition.

Over the last decades metalloporphyrins have been studied in great detail as they exhibit a high chemical and thermal stability, are aromatic and possess distinctive electrochemical and photophysical properties [1–4]. For example, access to the first organic spin valves, which were based on tris(8-hydroxyquinolinato)aluminium (Alq_3_) sandwiched between La_2/3_Sr_1/3_MnO_3_ and cobalt electrodes, was reported more than a decade ago [5]. This finding motivated the development of further novel devices as, for example, spin-OFETs (organic field effect transistors) [4]. The nature of the molecules integrated into spintronic devices ranges from purely diamagnetic molecules to individual single molecule magnets (SMMs) [4]. Among such molecules metalloporphyrins are very promising in terms of diverse applications [4]. Recently, we reported on the deposition of thin films of porphyrins of the type H_2_TPP(OH)_4_ (tetra(*p*-hydroxyphenyl)porphyrin) [6–7] and MTPP(OMe)_4_/H_2_TPP(OMe)_4_ (tetra(*p*-methoxyphenyl)porphyrin) (M = Cu [8–9], Ni [9]), cf. Figure 1.

The properties of the metalloporphyrins are governed by the (transition) metal ions and the exocyclic moieties on the individual pyrrole fragments and/or on the meso positions. Comparative studies of the accessibility and characterization of metalloporphyrins are scarcely reported in literature [1–3,10–12], which limits, for example, the possibility to select a certain metalloporphyrin with respect to a desired property by a knowledge-based approach. Along with a preliminary work of us, we noticed that “[…]the electrical analysis and the understanding of the underlying transport mechanism become important for future implementation of porphyrin-based (spintronic) devices.[…]” [8]. It was thus desired to have access to metalloporphyrins of which the central metal ion varies on the one hand, while on the other hand these metalloporphyrins should be sterically more demanding to vary the film morphology compared to our original report [8]. In order to support the idea that different central metals as well as sterically more demanding substituents will vary film morphologies one can, for example, inspect the results of the single-crystal crystallographic characterization even of the compounds displayed in Figure 1. It is instructive to notice, that for ZnTPP(OMe)_4_ [13] the formation of 2D layers is observed in which symmetry-related molecules with planar porphyrin cores interact with each other by, for example, formation of intermolecular Zn^II…^O contacts. Further intermolecular interactions refer to those that were described in detail by, for example, Goldberg et al. [14] or by us [15]. In contrast, saddle-shape distorted molecules of CuTPP(OMe)_4_ are described as interacting via C–H^…^π and C–H^…^O bonds to give a 3D supramolecular motif [16]. Furthermore, if one substitutes the terminal methyl substituents of H_2_TPP(OMe)_4_ (Figure 1) by sterically more demanding substituents as reported for H_2_TPP(OR)_4_ (OR = *p*-(*N*-*n*-butylcarbamoyl)methoxyphenyl) [17] one decreases the density to the materials to ρ = 1.036 g/cm^3^ compared to ρ = 1.491 g/cm^3^ for ZnTPP(OMe)_4_ [13] or ρ = 1.398 g/cm^3^ for CuTPP(OMe)_4_ [16].

Thus, we report herein on two novel series of (metallo)porphyrins of the type H_2_/MTPP(C(O)N(R)_2_)_4_ (R = Me, with H_2_TPP(C(O)NMe_2_)_4_ (**2**) and MTPP(C(O)N(iPr)_2_)_4_ (M = Zn (**2a**), Cu (**2b**), Ni (**2c**), Co (**2d**); R = iPr, with H_2_TPP(C(O)N(iPr)_2_)_4_ (3) and MTPP(C(O)N(iPr)_2_)_4_ (M = Zn (**3a**), Cu (**3b**), Ni (**3c**), Co (**3d**)). The aim of this report is not only to describe their synthesis and characterization (ESIMS, FTIR, NMR, UV–vis) but also to study to which extend these new (metallo)porphyrins are suitable to be deposited in form of thin films by OMBD. Therefore, the thermal stabilities derived from TG studies of selected representatives of **2/2a**–**d** and **3/3a**–**d** in comparison with that of H_2_TPP(OH)_4_ [6–7] will be discussed together with the results of OMBD studies.

## Results and Discussion

### Synthesis

**Scheme 1:**
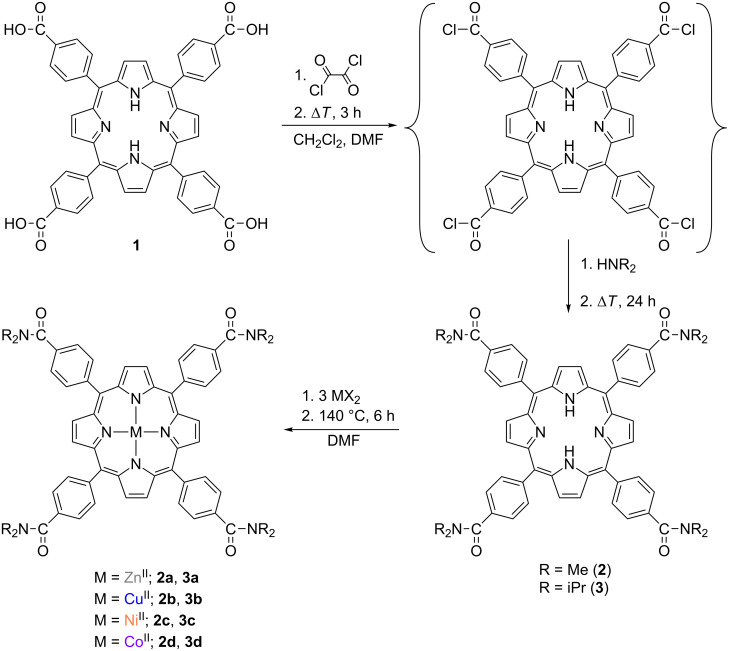
Synthetic methodology to prepare (metallo)porphyrins **2, 2a–d** and **3, 3a–d**.

Porphyrins **2** and **3** were synthesized as shown in Scheme 1 according to a procedure reported by Gradl et al. [18]. Literature-known H_2_TPP(CO_2_H)_4_ (**1**) was treated first with an excess of oxalyl chloride in dichloromethane in the presence of dimethylformamide. As we used a larger amount of dimethylformamide as indicated in [18], the yields of **2** and **3** could be increased significantly. This is attributed to the solubility of **1** in dimethylformamide. The addition of a large excess of the mild chlorinating agent oxalyl chloride converted **1** to H_2_TPP(C(O)Cl)_4_ (Scheme 1) which further reacted with the secondary amines HNMe_2_ and HN(iPr)_2_ to give **2** (H_2_TPP(C(O)NMe_2_)_4_) and **3** (H_2_TPP(C(O)N(iPr)_2_)_4_). The molar excess of oxalyl chloride compared to **1** should be above 25:1, as otherwise **1** cannot be fully converted to H_2_TPP(C(O)Cl)_4_. However, the use of thionyl chloride to convert **1** to H_2_TPP(C(O)Cl)_4_ is accompanied by chlorination of the β-pyrrolic positions. After formation of H_2_TPP(C(O)Cl)_4_ all volatiles must be removed in vacuum in order to avoid, for example, unwanted reactions upon the addition of HNMe_2_ and HN(iPr)_2_. Appropriate work-up, gave **2** and **3** in yields exceeding 80% without any column-chromatographic purification (cf. Experimental section).

The metalation reactions performed in this study correspond to the well-known “dimethylformamide method” (M^II^ = Zn, Cu, Ni, Co), cf. Scheme 1 and [19]. In agreement with details reported for the dimethylformamide method, “[…]best results have been obtained with anhydrous metal chlorides[…]” [19], although the reaction temperatures should be kept at 140 °C. According to [19], complete metalation needs the subsequent addition of an excess of the metal chlorides. Hence, we decided to use initially an excess of the metal chlorides. The metalloporphyrins **2a**–**d** and **3a**–**d** (Scheme 1) have been obtained in yields exceeding 60%. No purification by column chromatography was required although in case of **2d**, **3a** and **3d** the metalloporphyrins were re-precipitated for purification purposes (cf. Experimental section).

The purity of **2, 2a–d** and **3, 3a–d** was determined by CHN elemental analysis (EA), although this method has limits. For example, it is difficult to recognize by EA the presence of traces of impurities below ca. 0.5%. Furthermore, the measurement conditions of an EA may influence results as recently demonstrated for a series of octachlorometallophthalocyanines of the type MPcCl_8_ (M^II^ = Cu, Ni, Co, Fe, Mn) [20]. However, for the herein reported porphyrins **2** and **3** and their corresponding metalloporphyrins **2a**–**d** and **3a**–**d** the CHN contents deviate by at most ±0.5%. Since **2**/**3** and **2a**–**d**/**3a**–**d** are well soluble in solvents such as CH_2_Cl_2_, CHCl_3_, MeCN, DMSO, DMF it is possible to follow certain “criteria of purity” established by White, Bachmann and Burnham [21]. Thus, analytical amounts of these (metallo)porphyrins were chromatographed by thin layer chromatography (TLC) on alumina by using CHCl_3_/n-hexane mixtures (ratio 1:1, v/v) as eluent, showing that they were formed in high purity.

Furthermore, ^1^H NMR studies allowed us to monitor the progress of the metalation reactions of **2** and **3**, even for the paramagnetic metalloporphyrins **2b**,**d** and **3b**,**d**. For example, the complete metalations of the free-base porphyrins **2** and **3** are indicated by the disappearance of their N–*H* ^1^H NMR resonances.

### Electrospray ionization mass spectrometry

High-resolution mass spectrometry (HRMS) studies enable one to verify the successful formation of **2**/**3** and of **2a**–**d**/**3a**–**d**. The ESIMS measurements in positive-ionization mode were performed under identical conditions, including the use of MeCN/CH_2_Cl_2_ solutions of the respective (metallo)porphyrin. The ESIMS spectra and the respective isotopic patterns of the ion peaks in form of [M]^+^, [M + H]^+^, [M + Na]^+^ or [M + K]^+^ agree to the calculated ones (cf. the ESIMS spectra in Supporting Information File 1). In agreement with Buchler [19] and Budzikiewicz [22] the mass spectrometric measurements served well to identify the type of the incorporated transition metal since the ion peaks of [M]^+^ and/or [M + H]^+^ are the ones with the highest intensity. The observation of [M + Na]^+^ as well as [M + K]^+^ ions and of cations of low *m*/*z* values, for example [393]^+^ (observed in the ESIMS spectra of **2c**,**d** and **3c**,**d**), is due to contaminants that typically appear in such measurements as described in the literature [23–24]. For **2b**,**c**, **3** and **3a**–**c** double charged ion peaks are visible, clearly identifiable by an isotopic peak distance of *m*/*z* = 0.5. This is a common occurrence in ESI measurements when a higher concentration of the analyte is present [23].

### IR studies

**Figure 2:**
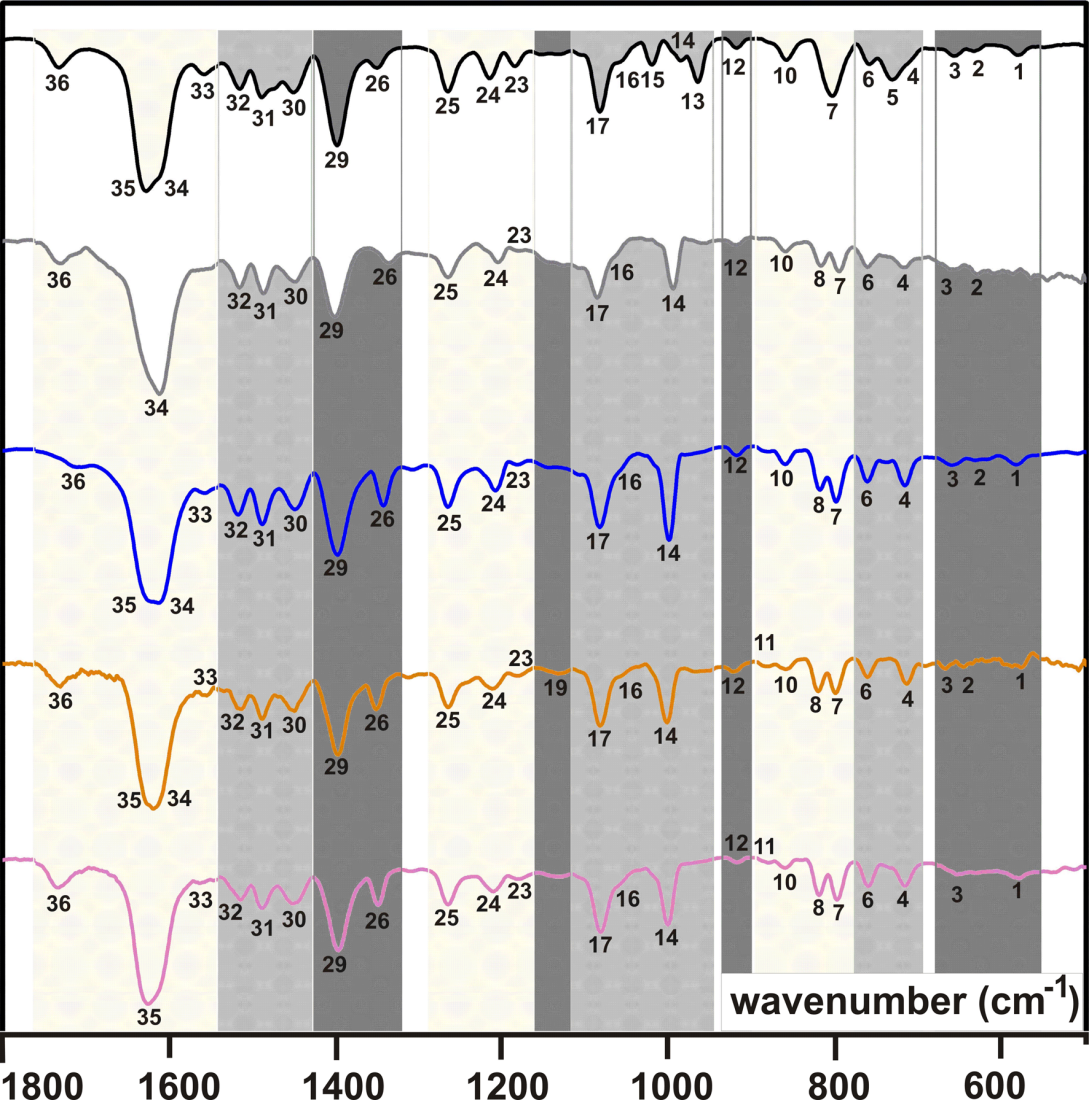
IR spectra (KBr) in the range of 500–1800 cm^−1^ for H_2_TPP(CONMe_2_)_4_ (**2**, top) and MTPP(CONMe_2_)_4_ (M^II^ = Zn, **2a** (gray); Cu, **2b** (blue); Ni, **2c** (orange); Co, **2d** (purple)).

**Figure 3:**
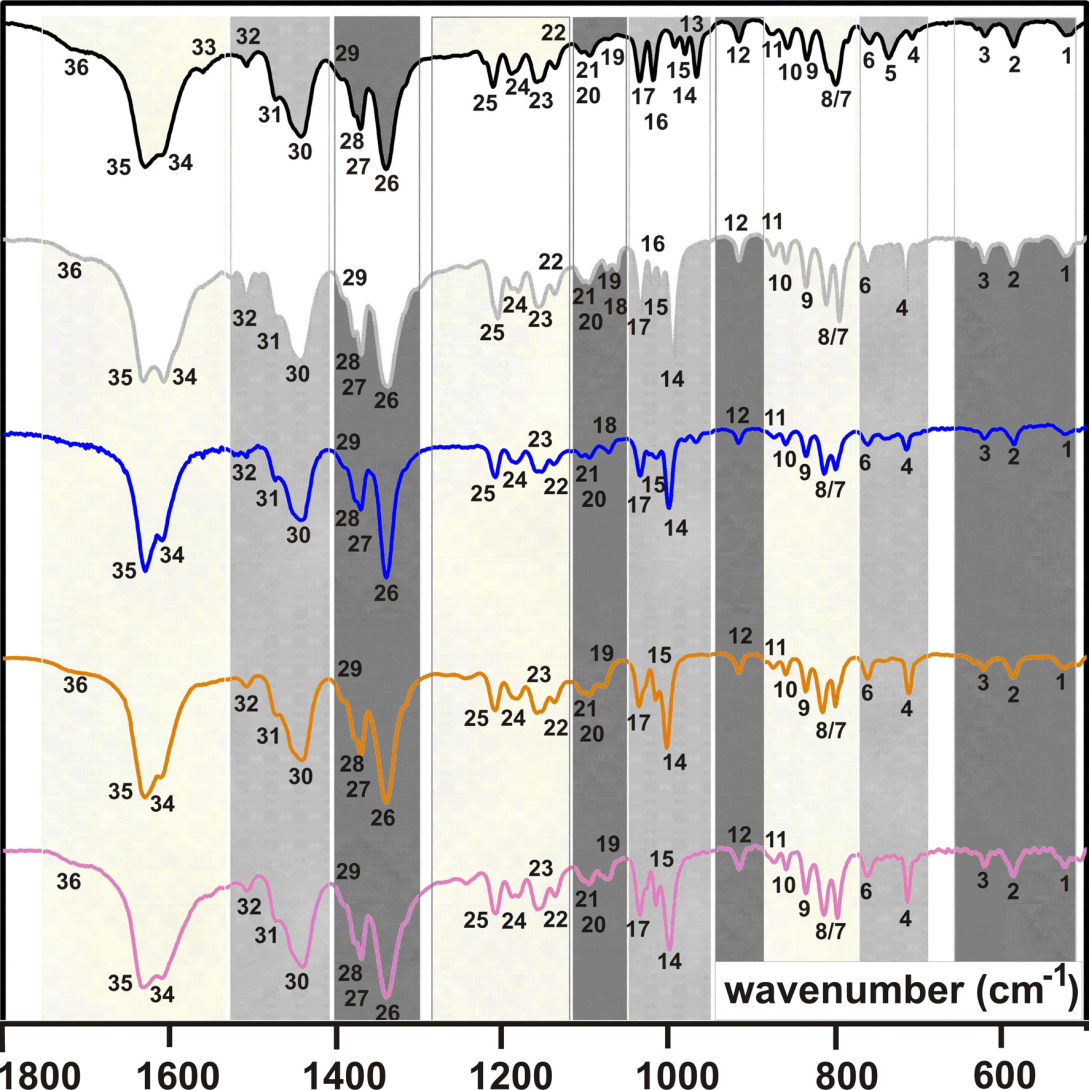
IR spectra (KBr) in the range of 500–1800 cm^−1^ for H_2_TPP(CON(iPr)_2_)_4_ (**3**, top) and MTPP(CON(iPr)_2_)_4_ (M^II^ = Zn, **3a** (gray); Cu, **3b** (blue); Ni, **3c** (orange); Co, **3d** (purple)).

Severe difficulties were noticed when measuring KBr pellets of **2**/**3** and **2a**–**d**/**3a**–**d**, as described by Alben [25]. These difficiculties are due to, for example, the optical inhomogeneity of the pellets. In order to avoid them, and as suggested by Alben [25], all (metallo)poprhyrins were intensively grinded to a fine flour before further grinding with KBr was done. It must be emphasized that due to the recommended intense and thus time-consuming grinding of the pure (polycrystalline) materials the IR spectra reveal the presence of water, likely due to the hygroscopic nature of the compounds and/or of KBr. In Figure 2 (**2**, **2a**–**d**) and Figure 3 (**3**, **3a**–**d**) the spectral region between 500 and 1800 cm^−1^ is displayed. Shaded areas within individual IR spectra displayed in Figure 2 and Figure 3 belong to related absorptions and are numbered. The wavenumbers of these absorptions are summarized in Table 1 for **2/2a**–**d** and **3**/**3a**–**d**. Full IR spectra (KBr) of **2**/**3** and of **2a**–**d**/**3a**–**d** are given in Supporting Information File 1. Furthermore, Supporting Information File 1 shows the IR spectra of **2**/**3** and of **2a**–**d**/**3a**–**d** as obtained by FTIR measurements with a Nicolet iS10 spectrometer (ATR attachment, ZnSe crystal) for comparison.

**Table 1:** Wavenumbers of numbered IR vibrations of **2**/**2a**–**d** and **3**/**3a**–**d** in the range from 500–1800 cm^−1^.^a^

no.	H_2_		Zn		Cu		Ni		Co	

	**2**	**3**	**2a**	**3a**	**2b**	**3b**	**2c**	**3c**	**2d**	**3d**

1	582	524	—	524	585	526	588	526	581	528
2	632	587	630	587	634	588	647	588	632	587
3	658	622	659	622	668	622	668	623	656	623
4	711	709	718	716	719	715	715	712	718	715
5	732	737	—	—	—	—	—	—	—	—
6	760	760	762	762	761	762	762	762	761	762
7	804	800	796	797	800	798	800	801	798	800
8	—	810	818	812	819	815	822	816	820	815
9	—	835	—	836	—	836	—	837	—	836
10	860	859	860	860	861	860	860	860	861	861
11	—	878	—	875	—	875	883	876	882	875
12	920	917	919	917	918	917	921	917	919	917
13	966	968	—	—	—	—	—	—	—	—
14	987	982	996	996	1000	1000	1003	1003	1002	1001
15	1021	994	—	1011	—	1016	—	1016	—	1017
16	1059	1019	1063	1021	1059	—	1055	—	1057	—
17	1084	1036	1086	1034	1083	1035	1083	1037	1082	1036
18	—	—	—	1063	—	1072	—	—	—	—
19	—	1073	—	1076	—	—	1030	1078	1134	1072
20	—	1096	—	1097	—	1095	—	1096	—	1096
21	—	1106	—	1105	—	1105	—	1106	—	1106
22	—	1137	—	1138	—	1138	—	1137	—	1138
23	1186	1160	1180	1156	1182	1158	1180	1161	1180	1160
24	1216	1190	1205	1181	1206	1190	1211	1183	1211	1186
25	1266	1212	1264	1206	1266	1209	1265	1209	1265	1209
26	1351	1340	1337	1339	1345	1340	1351	1340	1349	1339
27	—	1370	—	1370	—	1371	—	1370	—	1370
28	—	1378	—	1378	—	1378	—	1379	—	1379
29	1399	1395	1400	1390	1398	1391	1397	1393	1397	1392
30	1451	1442	1448	1443	1450	1441	1450	1441	1451	1440
31	1489	1473	1487	1472	1489	1472	1489	1472	1488	1473
32	1516	1508	1515	1507	1518	1506	1514	1507	1514	1508
33	1558	1561	—	—	1560	—	1556	—	1560	—
34	1609	1609	1612	1607	1608	1608	1619	1610	—	1609
35	1628	1630	—	1632	1622	1632	1626	1630	1625	1629
36	1732	1701	1730	1700	1711	—	1730	1710	1733	1699

^a^cf. Figure 2 and Figure 3.

For the porphyrins **2** and **3** three different N–H vibrations at 3310–3326 cm^−1^, 975–990 cm^−1^ and 675–700 cm^−1^ are expected according to [25]. The one observed at 3317 cm^−1^ for both **2** and **3** (Supporting Information File 1) fits well into the expected range. The vibrations no. 5 and no. 13 for **2** (966 and 732 cm^−1^) and **3** (968 and 737 cm^−1^), cf. Figure 2 and Figure 3 and Table 1, are attributed to the other two N–H vibrations. They deviate to some extend from the expected ranges, see above, but the corresponding metalloporphyrins do not show related vibrations (Figure 2 and Figure 3).

The spectral range from 3000 to 2800 cm^−1^ is governed by ν_as_(C–H) and ν_s_(C–H) absorptions of the aliphatic substituents R of the –C(O)NR_2_ groups of both **2/2a**–**d** and **3/3a**–**d** (Supporting Information File 1). According to [26], CH_3_ groups can be identified by one ν_as_(C–H) absorption at ca. 2950 cm^−1^ and up to two ν_s_(C–H) absorptions at lower spatial frequencies of ca. 2800 cm^−1^. The number of CH_3_ groups is eight for **2/2a**–**d**, that of **3/3a**–**d** is 16. This difference is nicely reflected in the intensities and shapes of the ν_as_(C–H) and ν_s_(C–H) absorptions. Among **2/2a**–**d** only for **2a** and **2c** all three possible absorptions could be observed, while further members exhibit only one ν_s_(C–H) and the ν_as_(C–H) vibration (Supporting Information File 1). For **3/3a**–**d** the ν_as_(C–H) vibration is always the most intensive one at 2970 ± 1 cm^−1^, followed by a less intensive first ν_s_(C–H) absorption (2932 ± 1 cm^−1^) and a third even less intensive ν_s_(C–H) band (2874 ± 4 cm^−1^). Due to these different spectral features it is possible to differentiate between a type **2/2a**–**d** or **3/3a**–**d** (metallo)porphyrin.

For the porphyrin cores and the aromatic C_6_H_4_ moieties, respectively, ν(C
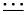
H) and ν(C=H) vibrations are expected above 3000 cm^−1^. However, these vibrations as well as combinations of γ(C
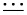
H) vibrations between 2000 and 1600 cm^−1^, could not be identified unambiguously or were too weak. Likely, this is due to the substitution of the aromatic C_6_H_4_ rings, decreasing the intensities of these vibrations [26].

The presence of CH_3_ groups in a compound is indicated in the IR spectra in general by one δ_as_(C–H) (ca. 1465 cm^−1^) vibration and at least one δ_s_(C–H) (ca. 1380 cm^−1^) vibration [26]. Furthermore, a single δ_s_(C–H) absorption verifies that the CH_3_ group belongs to an aliphatic chain that is not branched, or that the Me group is terminal as in the –NMe_2_ entities of **2/2a**–**d**. For branched alkyl chains the δ_s_(C–H) vibration splits into two [26]. Thus, the absorptions no. 30 and no. 26 of **2/2a**–**d** (1450 ± 2 cm^−1^ and 1344 ± 7 cm^−1^) are attributed to the δ_as_(C–H) and δ_s_(C–H) vibrations of the terminal CH_3_ groups (Figure 2 and Table 1). Due to a larger number of CH_3_ groups in **3/3a**–**d** compared to **2/2a**–**d** the ν_as_(C–H), ν_s_(C–H), δ_as_(C–H) and δ_s_(C–H) absorptions of **3/3a**–**d** are more intensive compared to **2/2a**–**d**. For example, the absorption no. 30 of **3/3a**–**d** (δ_as_(C–H), 1442 ± 2 cm^−1^) is significantly more intensive compared to **2/2a**–**d** (Figure 2, Figure 3 and Table 1). As expected, for **3/3a**–**d** two δ_s_(C–H) vibrations are observed, see no. 28 (1379 ± 1 cm^−1^) and no. 27 (1371 ± 1 cm^−1^) in Figure 3 and Table 1. The presence of iPr groups in **3/3a**–**d** was recognized further by their skeletal vibrations at 1158 ± 3 cm^−1^ (no. 23), shouldered at 1136 ± 2 cm^−1^ (no. 22) [21], while for **2/2a**–**d** only a weak absorption at 1183 ± 3 cm^−1^, denoted as no. 23, is observed.

For para-substituted C_6_H_4_ aromatic units one γ(C
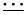
H) absorption between 800 and 860 cm^−1^ is expected [27], which is one of absorptions no. 7, 8 or 10 of **2/2a**–**d** and **3/3a**–**d,** (Figure 2, Figure 3 and Table 1). A more precise assignment is not possible, because C
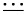
H vibrations of the β-pyrrolic hydrogens are expected to lead to absorptions at 772–805 cm^−1^ [27]. Further β-pyrrolic C
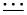
H vibrations are expected at 1045–1065 cm^−1^ [13], and thus no. 17 of **2/2a**–**d** and **3/3a**–**d** can be assigned to them (Figure 2, Figure 3 and Table 1).

The two strongest absorptions of **2/2a**–**d** and **3/3a**–**d** are due to ν(C
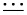
C) vibration of the aromatic moieties and ν(C=O) vibrations of the terminal –C(O)NR_2_ groups [27]. The ν(C
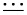
C) vibrations are expected at ca. 1600 cm^–1^, while the more intense ν(C=O) are observed between 1650 and 1690 cm^−1^ [27]. This allows for an assignment of no. 35 and no. 34 (Figure 2, Figure 3 and Table 1) to the former and the latter type of vibration, respectively. However, **2/2a**–**d** always exhibit one broad absorption band at ca. 1620 cm^−1^, which hinders a more precise assignment. For **3/3a-d** this situation is different and these two absorption bands occur well resolved. Most likely, that difference can be attributed to the different substitution of the terminal –C(O)NR_2_ groups.

### UV–vis studies

**Figure 4:**
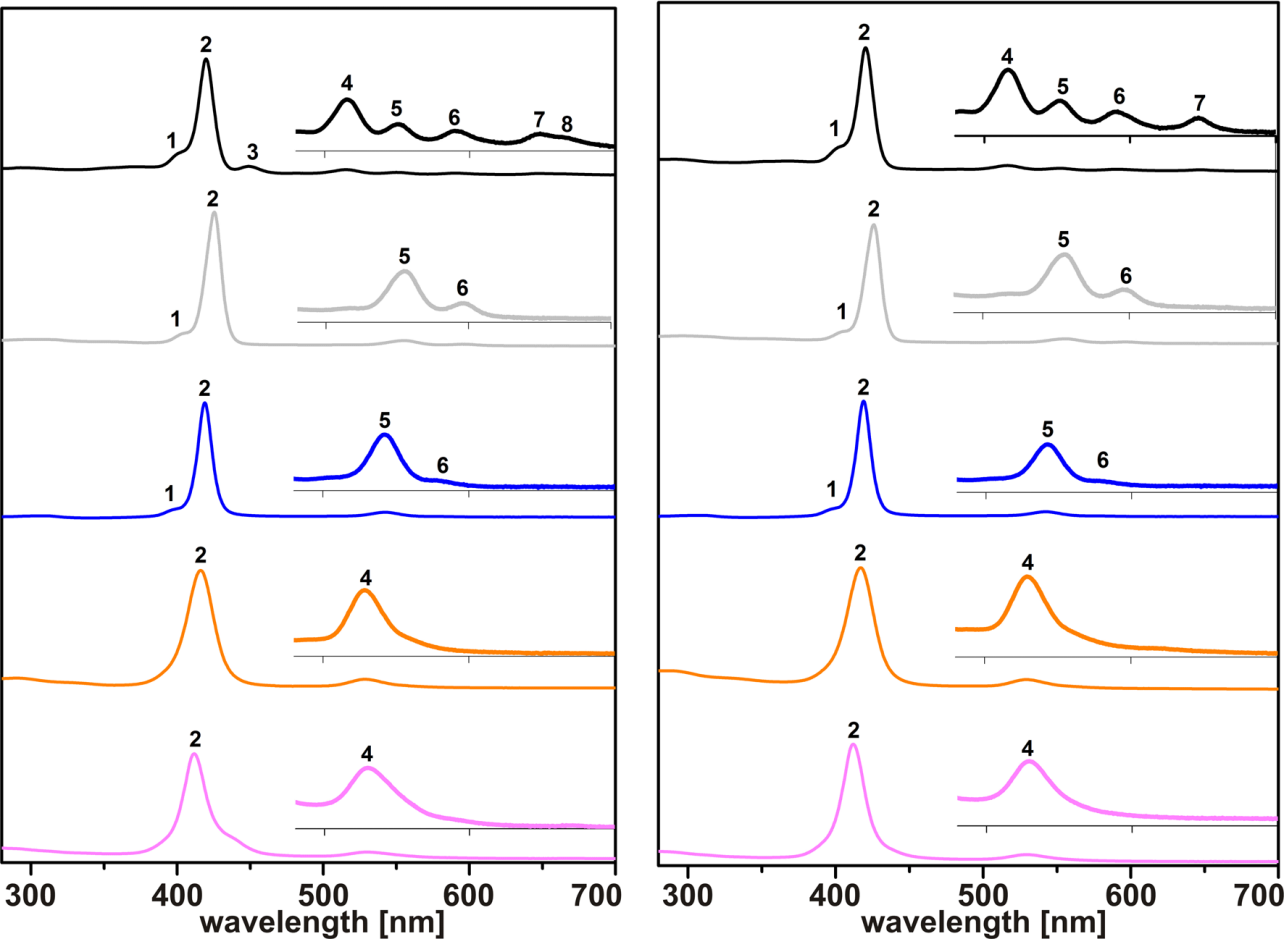
Left: UV–vis spectra (CHCl_3_, 280–700 nm) of H_2_TPP(C(O)NMe_2_)_4_ (**2**) and MTPP(C(O)NMe_2_)_4_ (M^II^ = Zn, **2a** (gray); Cu, **2b** (blue); Ni, **2c** (orange); Co, **2d** (purple)). Right: UV–vis spectra (CHCl_3_, 280–700 nm) of H_2_TPP(C(O)N(iPr)_2_)_4_ (**3**) and MTPP(C(O)N(iPr)_2_)_4_ (M^II^ = Zn, **3a** (gray); Cu, **3b** (blue); Ni, **3c** (orange); Co, **3d** (purple)).

The UV–vis absorption spectra of **2/2a**–**d** and **3/3a**–**d** were recorded in CHCl_3_ solution in the spectral range of 230–700 nm. In order to avoid possible impact of the concentrations on λ_abs_ and ε, which was reported for (metallo)phthalocyanines [28], we performed concentration-dependent UV–vis measurements. According to [28] the nature (cofacial, face-to-face, tilted) and degree (dimer, oligomer, polymer) of mutual interactions between (metallo)phthalocyanine molecules might modify their optical absorption spectra [28]. However, the UV–vis studies of **2/2a**–**d** and **3/3a**–**d** with varying concentrations revealed marginal impact on λ_abs_ (max. ±1 nm) and ε (max. ±4%), see Supporting Information File 1. Larger deviations of ε are attributed to random errors due to, for example, uncertainties in diluting the sample solutions. The UV–vis spectra of **2/2a**–**d** and **3/3a**–**d** displaying the absorption spectral range from 280–700 nm are shown in Figure 4. For better comparison we select the spectrum of an individual (metallo)porphyrin in which the maximum of the absorption is closest to 1.5 (Supporting Information File 1). Inserts in Figure 4 correspond to the enlarged spectral range of 480–700 nm. Optical absorptions are numbered in relation to the wavelength, λ_abs_ and log ε values are summarized in Table 2.

**Table 2:** Wavelengths of UV–vis absorption bands of **2**/**2a**–**d** and **3**/**3a**–**d** in the range of 280–700 nm.^a^

compound	absorption band no.							

	1	2	3	4	5	6	7	8

	λ_abs_ (log ε)							

**2**	401 (4.95)	420 (5.64)	449 (4.74)	516 (4.35)	551 (4.08)	591 (3.96)	647 (3.93)	666 (3.93)
**3**	400 (4.80)	421 (5.49)	—	517 (4.17)	552 (3.91)	590 (3.74)	648 (3.58)	—

**2a**	403 (4.55)	426 (5.55)	—	—	555 (4.19)	596 (3.73)	—	—
**3a**	404 (4.33)	426 (5.29)	—	—	556 (3.93)	597 (3.53)	—	—

**2b**	396 (4.44)	419 (5.65)	—	—	542 (4.28)	578 (3.39)	—	—
**3b**	397 (4.58)	419 (5.74)	—	—	542 (4.37)	579 (3.43)	—	—

**2c**	—	416 (5.30)	—	528 (4.17)	—	—	—	—

**3c**	—	417 (5.32)	—	530 (4.23)	—	—	—	—
**2d**	—	412 (5.24)	442 (4.44)	530 (4.03)	—	—	—	—
**3d**	—	412 (5.47)	—	529 (4.25)	—	—	—	—

^a^cf. Figure 4

Generally, absorption spectra of free-base porphyrins consist of characteristic absorption bands: The more intense Soret band (or *B* band) arising from a_1u_(π)→e_g_*(π) transitions and two *Q* bands (*Q*_x_(0,0) and *Q*_y_(0,0) from a_2u_(π)→e_g_*(π) transitions [29–30]. According to Goutermann the *B*(0,0) band appears between 380 and 420 nm (ε > 10^5^ M^−1^·cm^−1^) and is accompanied in case of well-resolved spectra by a blue-shifted (ca. 1250 cm^−1^) *B*(1,0) band [29–30]. *Q*-band absorptions occur in the spectral region between 500 and 700 nm (ε > 10^4^ M^−1^·cm^−1^) [29–30]. The *Q*_x_(0,0) and *Q*_y_(0,0) bands of *D*_2_*_h_*-symmetric porphyrins, separated by ca. 3000 cm^−1^, might be observed inclusive a vibronic overtone absorption of each *Q* band, denoted as *Q*_x_(1,0) and *Q*_y_(1,0) [29–30]. For metalloporphyrins adapting *D*_4_*_h_*-type symmetry, the four *Q* bands are observed to collapse into two *Q* bands, in some cases into only one [19,29]. The accompanying “[…]Soret band may remain in the usual range or shifted to higher or lower frequency.[…]”, according to Buchler [19]. Furthermore, (metallo)porphyrins may show a weak *N* (ca. 325 nm) and *M* band (ca. 215 nm), often with an even weaker *L* band [29].

As expected, for **2** and **3** the intensive *B*(0,0) band appears at ca. 420 nm (no. 2 in Figure 4, Table 2) and is followed by four significantly weaker *Q* bands at ca. 516, 551, 591 and 647 nm (no. 4–7 in Figure 4, Table 2). The separation between absorption no. 4 and no. 6 as well as between no. 5 and no. 7 amounts to, respectively, 2394 cm^−1^ as well as 2684 cm^−1^ for **3**, in good agreement with the expected difference between the *Q*_x_(0,0) and *Q*_y_(0,0) band of free-base porphyrins (see below). The blue-shifted shoulder of the *B*(0,0) band at 401/400 nm (no. 1 in Figure 4, Table 2) corresponds to the *B*(1,0) band of **2** and **3**, confirmed by blue-shifts of 1128 and1247 cm^−1^ (see above). As described earlier, and due to symmetry reasons, for Zn^II^- and Cu^II^-containing **2a**/**3a** and **2b**/**3b**, two *Q* bands are observed, while Ni^II^- and Co^II^-containing **2c**/**3c** and **2d**/**3d** possess only one *Q* band (Figure 4). The difference in numbers of the *Q* bands could be caused by a higher molecular symmetry of **2c**/**3c** or **2d**/**3d** compared to **2a**/**3a** and **2b**/**3b**, but is most likely attributable to weak perturbations by the central metal according to Goutermann [29]. A comparison of the λ_abs_ values of both the *B*(0,0) and the *Q* band(s) along **2/2a**–**d** and **3/3a**–**d** reveals a red-shift along the series Co^II^ < Ni^II^ < Cu^II^ < Zn^II^ (Figure 4 and Table 2). This observation is in agreement with observations summarized by Buchler [19] and Goutermann [29]. The same tendency has been observed more recently [11] and no significant differences of λ_abs_ values have been noticed [12], although the UV–vis spectra were recorded in both cases in CHCl_3_.

### Thermogravimetric studies

Part of our motivation to synthesize **2/2a**–**d** and **3/3a**–**d** originates from a number of cooperations with our partners in the DFG-supported research unit “Towards Molecular Spintronics” [6–9]. For example, (metallo)porphyrins were synthesized and deposited by OMBD for different kinds of physical thin-film studies [6–9]. In one of these contributions thin films of CuTPP(OMe)_4_ (Figure 1) were investigated by current-sensing atomic force microscopy [8]. It was concluded that for the investigation of films with different morphologies and transport properties further (metallo)porphyrins should be studied, as outlined in the Introduction section [6–9].

However, we were not able to deposit thin films of **3**, **3b** and **3d** nor of **2**, **2c** and **2d** by means of OMBD. In more detail: OMBD parameters were initially chosen as reported in [8]. Thus, at 2 × 10^−7^ mbar a deposition rate of 5 Å/min was adjusted. In all investigated cases, deposition rates were not stable and constantly decreased over time. In order to maintain a stable deposition rate, the deposition temperatures were constantly increased from 300 to 350 °C in a Knudsen cell. After keeping the materials for ca. 20 min at these high temperatures, it was observed that the deposition rates dropped significantly. From this point onwards, it was not possible to perform any (further) deposition of the materials. In case of **3b** and **3d** the remaining material in the Knudsen cell was subjected to IR measurements (Supporting Information File 1) in comparison with measurements of the starting materials, showing that both metalloporphyrins decomposed during the OMBD studies.

**Figure 5:**
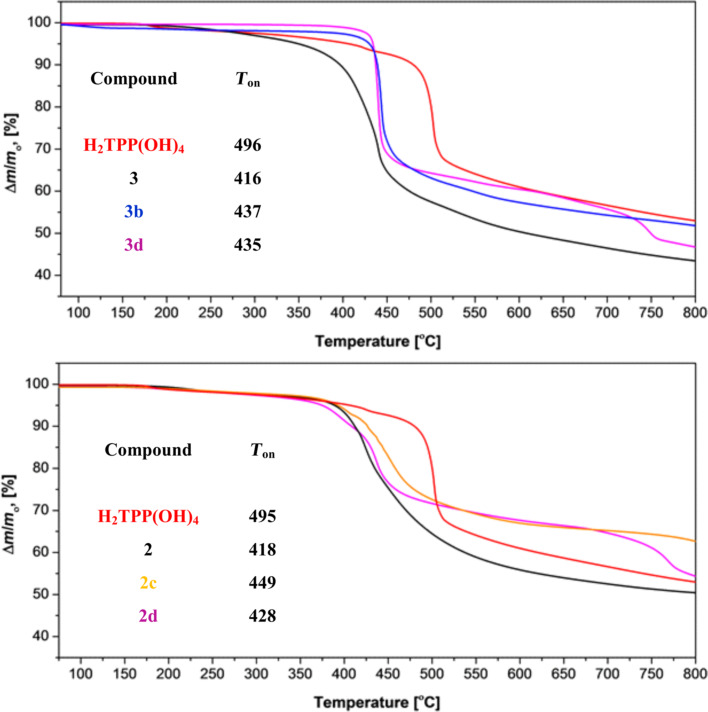
Top: TG traces of **3**, **3b** and **3d** in comparison with H_2_TPP(OH)_4_. Bottom: TG traces of **2**, **2c** and **2d** in comparison with H_2_TPP(OH)_4_.

In order to shine more light into the temperature stability we carried out TG studies for **3**, **3b**, **3d**, **2**, **2c** and **2d**. The TG traces are shown in Figure 5 together with the one of H_2_TPP(OH)_4_. In our earlier studies [6–7], H_2_TPP(OH)_4_ could be deposited successfully by applying OMBD parameters analogous the those described above. A comparison especially of the onset temperatures of the decomposition processes reveals that H_2_TPP(OH)_4_ is obviously significantly more thermally stable than the here reported (metallo)porphyrins. Because of this, OMBD of **2/2a**–**d** and **3/3a**–**d** is not possible and we are recently fabricating thin layers of these compounds by spin-coating [31].

## Conclusion

Two series of metalloporphyrins MTPP(C(O)NR_2_)_4_ (M = Co^II^, Ni^II^, Cu^II^, Zn^II^) derived out of their free-base species H_2_TPP(C(O)NR_2_)_4_ (R = Me (**2/2a**–**d**), iPr (**3/3a**–**d**)) were synthesized and characterized by NMR, IR and UV–vis spectroscopy as well as by ESI mass-spectrometry. The comparison of the obtained analytical results revealed only minor differences in vibrational and optical spectra, both with respect to the varied transition metal ions as well as the terminal organic substituent R. That provides potentially useful insight into the material properties of these porphyrins. It was anticipated that the variation of the central transition metal ions along **2a**–**d** and **3a**–**d** modify to the local transport characteristics of OMBD-deposited thin films of these compounds. In addition, in order to modify thin-film morphologies of successfully OMBD-deposited CuTPP(OMe)_4_ **2/2a**–**d** and **3/3a**–**d** were equipped with sterically more bulky terminal organic groups. Unfortunately, all trials to deposit members of **2/2a**–**d** and **3/3a**–**d** by OMBD failed, which is attributed to a significantly lower thermal stability compared to CuTPP(OMe)_4_ [8]. Most likely, the decreased thermal stability of **2/2a**–**d** and **3/3a**–**d** can be attributed to fragmentations of the terminal –C(O)NR_2_ functionalities during heating. Thus, this study shows that the thermal stability of (metallo)porphyrins is subjected to certain limits, and the application of other thin-film depositions techniques is required for **2/2a–d** and **3/3a–d**.

## Experimental

### General conditions

All chemicals were purchased from commercial sources and were used as received, unless stated otherwise. All reactions were carried out under argon atmosphere using standard Schlenk techniques and vacuum-line manipulations unless stated otherwise. All solvents were distilled prior to use and were purified/dried according to standard procedures [32].

### Starting materials

5,10,15,20-Tetra(4-carboxyphenyl)porphyrin (H_2_TPP(COOH)_4_, **1**) was synthesized according to [33] and MCl_2_·*n*H_2_O salts (M = Zn^II^, Cu^II^, Ni^II^, Co^II^) were dried according to [34].

### Instruments

NMR spectra were recorded at ambient temperature with a Bruker Avance III 500 Ultra Shield Spectrometer (^1^H at 500.300 MHz and ^13^C{^1^H} at 125.813 MHz) in the Fourier transform mode. Chemical shifts are reported in δ (ppm) versus SiMe_4_ with the solvent as the reference signal CDCl_3_: ^1^H NMR, δ = 7.26; and ^13^C{^1^H} NMR, δ = 77.16. FTIR spectra were recorded in the range of 400–4000 cm^−1^ with a Perkin-Elmer 1000 FTIR spectrometer as KBr pellets and in the range of 650–4000 cm^−1^ with a Thermo Scientific Smart iTR, Nicolet iS10. (The two absorptions at ca. 2360 cm^−1^, which appear different in intensity from spectra to spectra, are due to CO_2_.) C, H, N elemental analyses were performed using a Thermo FlashAE 1112 series analyzer. High-resolution mass spectra were recorded with a Bruker micrOTOF QII equipped with an Apollo II ESI source. UV–vis absorption spectra were recorded with a Spectronic GENESYS 6 UV–visible spectrophotometer (Thermo Electron Corporation) between 200–800 nm. TG experiments were performed using a Mettler Toledo TGA/DSC1 1600 system with an MX1 balance.

### Synthesis of **2**

To a suspension of **1** (1.00 g, 1.26 mmol) in dichloromethane (140 mL) dimethylformamide (1 mL, 12.9 mmol) was added. This reaction mixture was cooled to 0 °C and oxalyl chloride (3.20 mL, 37.31 mmol) was added dropwise (within 20 min) under continuous stirring. The mixture was stirred at 0 °C for further 30 min followed by refluxing for 3 h. After all volatiles were removed under reduced pressure the obtained crude product was dissolved in dichloromethane (30 mL) and a mixture of dimethylamine (2 M in tetrahydrofuran, 16 mL, 32 mmol) and triethylamine (1 mL, 7.17 mmol) was added dropwise at ambient temperature. The reaction mixture was stirred at this temperature for another 3 h, followed by refluxing for 24 h. Afterward, all volatiles were removed under reduced pressure and hot distilled water (100 mL) was added to the crude product with continuous stirring for 30 min. The purple precipitate formed was filtered off, washed with hot distilled water (5 × 20 mL) and dried at 110 °C in an oven. Yield: 0.91 g (80% based on **1**). Anal. calcd for C_56_H_50_N_8_O_4_ (899.05): C, 74.81; H, 5.61; N, 12.46; found: C, 74.3; H, 5.7; N, 12.2; ^1^H NMR (CDCl_3_) δ −2.80 (s, 2H, H^a,a′^), 3.32 (s, 24H, H^1,2^), 7.84 (d, 8H, H^6,6′^), 8.26 (d, 8H, H^5,5′^), 8.87 (s, 8H, H^10,10′^); ^13^C{^1^H} NMR (CDCl_3_) δ 35.80 (C^1^), 40.15 (C^2^), 119.62 (C^8^), 125.80 (C^6,6′^), 134.57 (C^7^), 135.93 (C^5,5′^), 143.46 (C^4^), 171.80 (C^3^); HRMS (ESI-TOF, positive mode, MeCN/CH_2_Cl_2_): *m*/*z* 899.4058 [**2 +** H]^+^, 937.3515 [**2 +** K]^+^; calcd for C_56_H_51_N_8_O_4_/C_56_H_50_KN_8_O_4_ ([**2 +** H]/[**2** + K]) = 899.4028/937.3587; IR (KBr, cm^−1^) ν: 3317 (w, N–H); 2929/2897/2866 (m/w/w, C–H); 1629/1609 (s/w, C=O); UV–vis (CHCl_3_) λ_abs_ [nm] (log ε): 401 (5.24), 420 (5.95), 449 (4.83), 516 (4.64), 551 (4.36), 591 (3.24), 647 (4.17), 666 (4.07); Supporting Information File 1 gives the IR, ^1^H NMR, ^13^C{^1^H} NMR, UV–vis and ESIMS spectra of **2**.

**Comments:** According to Jones and Wilkins [35] for the –NMe_2_ groups two ^13^C NMR chemical shifts are observed. According to Manke et al. [36] the ^13^C NMR resonances of the pyrrole carbon atoms C^9,9′^ and C^10,10′^ are not observable.

### Synthesis of **3**

To a suspension of **1** (1.00 g, 1.26 mmol) in dichloromethane (140 mL), dimethylformamide (1 mL, 12.9 mmol) was added. This reaction mixture was cooled to 0 °C and oxalyl chloride (3.20 mL, 37.31 mmol) was added dropwise (within 20 min) under continuous stirring. The mixture was stirred at 0 °C for further 30 min followed by refluxing for 3 h. After all volatiles were removed under reduced pressure the obtained crude product was dissolved in dichloromethane (30 mL), and a mixture of diisopropylamine (11.52 g, 0.114 mol, 16 mL) and triethylamine (1 mL, 7.17 mmol) was added dropwise at ambient temperature. The reaction mixture was refluxed for 24 h. After cooling to ambient temperature, all volatiles were removed under reduced pressure, and hot distilled water (100 mL) was added to the crude product under continuous stirring for 30 min. The purple precipitate formed was filtered off, washed with hot distilled water (5 × 20 mL) and dried at 110 °C. Yield: 1.21 g (85% based on **1**). Anal. calcd for C_72_H_82_N_8_O_4_ (1123.47): C, 76.97; H, 7.36; N, 9.97; found: C, 76.8; H, 7.2; N 9.9. ^1^H NMR (CDCl_3_) δ −2.78 (s, 2H, H^a,a′^), 1.43/1.66 (s(broad)/s(broad), 24H/24H, H^1,1′,2,2′^), 3.71/4.31 (s(broad)/s(broad), 4H/4H, H^3,3′^), 7.74 (d, 8H, H^7,7′^), 8.24 (d, 8H, H^6,6′^), 8.90 (s, 8H, H^11,11′^); ^13^C{^1^H} NMR (CDCl_3_) δ 21.24 (C^1,1′,2,2′^), 119.8 (C^9^), 124.6 (C^7,7′^), 134.8 (C^8^), 138.6 (C^6,6′^), 142.8 (C^5^), 171.3 (C^4^); HRMS (ESI-TOF, positive mode, MeCN/CH_2_Cl_2_): *m*/*z* 1123.6520 [**3** + H]^+^, 1145.6319 [**3 +** Na]^+^; calcd for C_72_H_83_N_8_O_4_/C_72_H_82_NaN_8_O_4_ ([3 **+** H]/[**3 +** Na]) = 1123.6532/1145.6351; IR (KBr, cm^−1^) ν: 3317 (w, N–H); 2969/2932/2874 (m/w/w, C–H); 1630/1608 (s, C=O); UV–vis (CHCl_3_) λ_abs_ [nm] (log ε): 400 (4.80), 420 (5.49), 482 (3.74), 517 (4.17), 552 (3.91), 591 (3.74), 648 (3.58). Supporting Information File 1 gives the IR, ^1^H NMR, ^13^C{^1^H} NMR, UV–vis and ESIMS spectra of **3**.

**Comments:** The ^1^H NMR resonances of the N(iPr)_2_ groups are all broadened. The hydrogen atoms H^1,1′,2,2′^ are regarded to correspond to the two broad singlets at 1.39 and 1.69 ppm. The hydrogen atoms H^3,3′^ are regarded to correspond to the two singlets at 3.71 and 4.31 ppm. Both assignments could, however, not be verified by additional 2D NMR experiments (^1^H,^1^H-COSY,^1^H,^13^C-HSQCETGP and HMBCGP) because of too broad NMR resonances and/or the comparatively poor solubility. According to Jones and Wilkins [35] for the –NMe_2_ groups two ^13^C NMR chemical shifts are observed. According to Manke et al. [36] the ^13^C NMR resonances of the pyrrole carbon atoms C^9,9′^ and C^10,10′^ are not observable.

### General procedure for the synthesis of **2a–d** and **3a–d**

**Unless stated otherwise, the following procedure was used:** To a solution of **2** (0.200 g, 0.222 mmol) for **2a**–**d**, or **3** (0.200 g, 0.178 mmol) for **3a**–**d** in dimethylformamide (25 mL), a solution of the MCl_2_ salt (3 equiv) in dimethylformamide (5 mL) was added dropwise (within 5 min) at ambient temperature. The reaction temperature was raised to 140 °C for 6 h. After cooling the reaction mixture to ambient temperature, chloroform (50 mL) was added and the combined organic phases were washed with water (3 × 40 mL) and brine (3 × 40 mL) to remove the excess of the MCl_2_ salt. The organic phase was dried over magnesium sulfate, and all volatiles were removed in vacuo to afford solids of the corresponding metalloporphyrins, which were dried additionally in vacuo for 12 h. Afterward, the corresponding solids were dissolved in CHCl_3_ and precipitated with *n*-hexane. That procedure is referred to in the following as “re-precipitation”.

### Data for **2a**

**2** (0.200 g, 0.222 mmol), ZnCl_2_ (0.0909 g, 0.667 mmol). Yield: 0.156 g (73% based on **2**); purple solid. Anal. calcd for C_56_H_48_N_8_O_4_Zn (962.44): C, 69.88; H, 5.03; N, 11.64; found: C, 69.5; H, 5.0; N, 11.5; ^1^H NMR (CDCl_3_) δ 3.16/3.26 (s/s, 12H/12H, H^1,2^), 7.68 (d, 8H, H^6,6′^), 8.23 (d, 8H, H^5,5′^), 8.93 (s, 8H, H^10,10′^); HRMS (ESI-TOF, positive mode, MeCN/CH_2_Cl_2_): *m*/*z* 960.3058/961.3149 [**2a**]^+^/[**2a +** H]^+^, 983.2908 [**2a +** Na]^+^, 999.2716 [**2a +** K]^+^; calcd for C_56_H_48_N_8_O_4_Zn/C_56_H_49_N_8_O_4_Zn, C_56_H_48_NaN_8_O_4_Zn, C_56_H_48_KN_8_O_4_Zn ([**2a**]/[**2a +** H], [**2a +** Na], [**2a +** K] = 960.3058/961.3163, 983.2982, 999.2722; IR (KBr, cm^−1^) ν: 2929 (w, C–H); 1612 (s, C=O); UV–vis (CHCl_3_) λ_abs_ [nm] (log ε): 403 (4.55), 426 (5.55), 555 (4.19), 596 (3.73). Supporting Information File 1 gives the IR, ^1^H NMR, UV–vis and ESIMS spectra of **2a**.

**Comments:** No re-precipitation needed. Due to the poor solubility of **2a** a ^13^C NMR spectrum could not be recorded. The ESIMS spectra of **2a** reveals as basis peak 988.3599. The origin of this peak remains unclear and may likely correspond to a fragmentation/recombination process under ESIMS measurement conditions.

### Data for **2b**

**2** (0.200 g, 0.222 mmol), CuCl_2_ (0.0897 g, 0.667 mmol). Yield: 0.130 g (61% based on **2**); wine red solid. Anal. calcd for C_56_H_48_CuN_8_O_4_ (960.58): C, 70.02; H, 5.04; N, 11.76; found: C, 69.9; H, 5.0; N, 11.6; HRMS (ESI-TOF, positive mode, MeCN/CH_2_Cl_2_): *m*/*z* 960.3254 [**2b**]^+^; calcd for C_56_H_48_CuN_8_O_4_ [**2b**] 960.3128; IR (KBr, cm^−1^) ν: 2928/2932 (w/w, C–H); 1622 (C=O); UV–vis (CHCl_3_) λ_abs_ [nm] (log ε): 396 (4.44), 419 (5.65), 543 (4.28), 578 (3.39). Supporting Information File 1 gives the IR, UV–vis and ESIMS spectra of **2b**.

**Comments:** No re-precipitation needed.

### Data for **2c**

**2** (0.200 g, 0.222 mmol), NiCl_2_ (0.0865 g, 0.667 mmol). Yield: 0.149 g (70% based on **2**); brown solid. Anal. calcd for C_56_H_48_N_8_NiO_4_ (955.72): C, 70.38; H, 5.06; N, 11.72; found: C, 70.1; H, 5.0; N, 11.6; ^1^H NMR (CDCl_3_) δ 3.27 (s, 24H, H^1,2^), 7.76 (d, 8H, H^6,6′^), 8.05 (d, 8H, H^5,5′^), 8.76 (s, 8H, H^10,10′^); ^13^C{^1^H} NMR (CDCl_3_) δ 24.41 (C^1^), 33.87 (C^2^), 118.47 (C^8^), 125.96 (C^6,6′^), 132.46 (C^10,10′^), 133.73 (C^7^), 135.96 (C^5,5′^), 142.20 (C^9,9′^), 142.67 (C^4^), 171.69 (C^3^); HRMS (ESI-TOF, positive mode, MeCN/CH_2_Cl_2_): *m*/*z* 955.3153 [**2c +** H]^+^; calcd for C_56_H_49_N_8_NiO_4_ [**2c +** H] = 955.3225; IR (KBr, cm^−1^) ν: 2924/2854 (w/w, C–H); 1626 (s, C=O); UV–vis (CHCl_3_) λ_abs_ [nm] (log ε): 416 (5.30), 528 (4.17). Supporting Information File 1 gives the IR, ^1^H NMR, ^13^C{^1^H} NMR, UV–vis and ESIMS spectra of **2c**.

**Comments:** No re-precipitation needed. Due to a better solubility of **2c** as compared to **2a**, ^13^C NMR spectra could be recorded. In contrast to comments made for **2**, all chemically different carbon atoms were observable, although for the –NMe_2_ groups of **2c** two ^13^C NMR resonances were observed as reported for **2**.

### Data for **2d**

**2** (0.200 g, 0.222 mmol), CoCl_2_ (0.0867 g, 0.667 mmol). Yield: 0.155 g (73%, based on **2**); wine red solid. Anal. calcd for C_56_H_48_CoN_8_O_4_ (955.96): C, 70.36; H, 5.05; N, 11.72; found: C, 70.1; H, 5.0;N, 11.7; HRMS (ESI-TOF, positive mode, MeCN/CH_2_Cl_2_): *m*/*z* 955.3125 [**2d**]^+^; calcd for C_56_H_48_N_8_CoO_4_ [**2d**] = 955.3125; IR (KBr, cm^−1^) ν: 2927/2852 (w/w, C–H); 1625 (s, C=O); UV–vis (CHCl_3_) λ_abs_ [nm] (log ε): 412 (5.24), 442 (4.44), 530 (4.03). Supporting Information File 1 gives the IR, UV–vis and ESIMS spectra of **2d**.

**Comments:** Re-precipitation needed.

### Data for **3a**

**3** (0.200 g, 0.178 mmol), ZnCl_2_ (0.0728 g, 0.534 mmol). Yield: 0.192 g (91% based on **3**); purple solid. Anal. calcd for C_72_H_80_N_8_O_4_Zn (1186.87): C, 72.86; H, 6.79; N, 9.44, found: C, 72.1; H, 6.6; N, 9.23; ^1^HNMR (CDCl_3_) δ 1.45/1.59 (s(broad)/s(broad), 24H/24H, H^1,1′,2,2′^), 3.68/4.31 (s(broad)/s(broad), 4H/4H, H^3,3′^), 7.65 (d, 8H, H^7,7′^), 8.22 (d, 8H, H^6,6′^), 8.98 (s, 8H, H^11,11′^); ^13^C{^1^H} NMR (CDCl_3_) δ 20.85 (C^1,1′,2,2′^), 120.46 (C^9^), 124.11 (C^7,7′^), 132.08 (C^11,11′^), 134.48 (C^8^), 137.85 (C^6,6′^), 143.34 (C^5^), 150.08 (C^10,10′^), 171.08 (C^4^); HRMS (ESI-TOF, positive mode, MeCN/CH_2_Cl_2_): *m*/*z* 1185.5632 [**3a +** H]^+^, 1207.5471 [**3a +** Na]^+^; calcd for C_72_H_81_ZnN_8_O_4_/C_72_H_80_NaZnN_8_O_4_ ([**3a + H**]/[**3a +** Na]) = 1185.5667/1207.5486; IR (KBr, cm^−1^) ν: 2969/2928/2869 (m/w/w, C–H); 1632 (s, C=O); UV–vis (CHCl_3_) λ_abs_ [nm] (log ε): 404 (4.33), 426 (5.29), 556 (3.93), 597 (3.53). Supporting Information File 1 gives the IR, ^1^H NMR, ^13^C{^1^H} NMR, UV–vis and ESIMS spectra of **3a**.

**Comments:** Re-precipitation needed. Because **3a** is better soluble than **2a**, ^13^C NMR spectra could be recorded. In contrast to comments made for **3**, all chemically different carbon atoms beside C^3,3′^ (belonging to the –N(iPr)_2_ groups) were observable. On the other hand, as discussed for **3** broad singlets in the ^1^H NMR spectra are regarded to correspond to the hydrogen atoms H^1,1′,2,2′,3,3′^.

### Data for **3b**

**3** (0.200 g, 0.178 mmol), CuCl_2_ (0.0718, 0.534 mmol). Yield: 0.124 g (59% based on **3**); wine red solid. Anal. calcd for C_72_H_80_CuN_8_O_4_(1185.0): C, 72.98; H, 6.80; N, 9.46; found: C, 72.5; H, 6.7;N, 9.4; HRMS (ESI-TOF, positive mode, MeCN/CH_2_Cl_2_): *m*/*z* 1184.5665 [**3b**]^+^; calcd for C_72_H_80_CuN_8_O_4_ [**3b**] = 1184.5671; IR (KBr, cm^−1^) ν: 2966/2928/2869 (m/w/w, C–H); 1632 (s, C=O); UV–vis (CHCl_3_) λ_abs_ [nm] (log ε): 397 (4.58), 419 (5.72), 542 (4.36), 579 (3.46). Supporting Information File 1 gives the IR, UV–vis and ESIMS spectra of **3b**.

**Comments:** No re-precipitation needed.

### Data for **3c**

**3** (0.200 g, 0.178 mmol), NiCl_2_ (0.0692 g, 0.534 mmol). Yield: 0.126 g (60%, based on **3**); brown solid. Anal. calcd for C_72_H_80_N_8_NiO_4_(1180.15): C, 73.28; H, 6.83; N, 9.49; found: C, 72.9; H, 6.8; N, 9.4; ^1^H NMR (CDCl_3_) δ 1.40/1.62 (s(broad)/s(broad), 24H/24H, H^1,1′,2,2′^), 3.70/4.23 (s(broad)/s(broad), 4H/4H, H^3,3′^), 7.66 (d, 8H, H^7,7′^), 8.03 (d, 8H, H^6,6′^), 8.79 (s, 8H, H^11,11′^); ^13^C{^1^H} NMR (CDCl_3_) δ 21.00 (C^1,1′,2,2′^), 118.55 (C^9^), 124.65 (C^7,7′^), 132.46 (C^11,11′^), 133.96 (C^8^), 138.57 (C^6,6′^), 141.34 (C^5^), 142.76 (C^10,10′^), 171.07 (C^4^); HRMS (ESI-TOF, positive mode, MeCN/CH_2_Cl_2_): *m*/*z* 1179.5713 [**3c +** H]^+^, 1201.5520 [**3c +** Na]^+^; calcd for C_72_H_81_NiN_8_O_4_/C_72_H_80_NaNiN_8_O_4_ ([**3c +** H]/[**3c +** Na]) = 1179.5729/1201.5548; IR (KBr, cm^−1^) ν: 2969/2928/2875 (w/w/w, C–H); 1630 (s, C=O); UV–vis (CHCl_3_) λ_abs_ [nm] (log ε): 417 (5.32), 530 (4.23). Supporting Information File 1 gives the IR, ^1^H NMR, ^13^C{^1^H} NMR, UV–vis and ESIMS spectra of **3c**.

**Comments:** No re-precipitation needed. As discussed for **3a** (above), analogous observations were made for **3c**.

### Data for **3d**

**3** (0.200 g, 0.178 mmol), CoCl_2_ (0.0693 g, 0.534 mmol). Yield: 0.164 g (78%, based on **3**); wine red solid. Anal. calcd for C_72_H_80_CoN_8_O_4_ (1180.39): C, 73.26; H, 6.83; N, 9.49; found: C, 72.8; H, 6.7; N, 9.3; HRMS (ESI-TOF, positive mode, MeCN/CH_2_Cl_2_): *m*/*z* 1179.5561 [**3d**]^+^; calcd for C_72_H_80_CuN_8_O_4_ [**3d**] = 1179.5629; IR (KBr, cm^−1^) ν: 2963/2931/2869 (m/w/w, C–H); 1629 (s, C=O); UV–vis (CHCl_3_) λ_abs_ [nm] (log ε): 412 (5.47), 529 (4.25). Supporting Information File 1 gives the IR, UV–vis and ESIMS spectra of **3d**.

**Comments:** Re-precipitation needed.

## Supporting Information

Supporting Information File 1 features ^1^H and ^13^C{^1^H} NMR spectra of **2**, **2a**, **2c**, **3**, **3a** and **3c**, ESIMS, UV–vis and IR spectra (ATR-IR and KBr) of **2**, **2a**–**2d**, **3** and **3a**–**3d**, and IR spectra of **3b** and **3d** before and after OMBD together with optical photographs of the materials.

File 1 Additional experimental data.
