# Supplementary material for: Synthesis, spectroscopic characterization and thermogravimetric analysis of two series of substituted (metallo)tetraphenylporphyrins
Source: Beilstein J Nanotechnol. 2017 Jun 2;8:1191–204. doi: 10.3762/bjnano.8.121 (PMC5480337; doi:10.3762/bjnano.8.121)
Supplement: File 1 — Additional experimental data. [file Beilstein_J_Nanotechnol-08-1191-s001.pdf]

# Supporting Information

for

# Synthesis, spectroscopic characterization and thermogravimetric analysis of two series of substituted (metallo)tetraphenylporphyrins

Rasha K. Al-Shewiki[1], Carola Mende[1], Roy Buschbeck[1], Pablo F. Siles[2,3], Oliver G. Schmidt[2,3], Tobias Rüffer*[1] and Heinrich Lang[1]

Address: [1]Inorganic Chemistry, Institute of Chemistry, Faculty of Natural Sciences, TU Chemnitz, 09107 Chemnitz, Germany; [2]Material Systems for Nanoelectronics, TU Chemnitz, 09107 Chemnitz, Germany and [3]Institute for Integrative Nanosciences, IFW Dresden, Helmholtzstrasse 20, 01069 Dresden, Germany

Email: Tobias Rüffer* **-** tobias.rueffer@chemie.tu-chemnitz.de

* Corresponding author

## Additional experimental data

# Table of contents


| | | |
|---|---|---|
| Figure S1 | $^{1}$H and $^{13}$C{$^{1}$H} NMR spectra of **2** | S4 |
| Figure S2 | ESI-MS spectrum of **2** | S5 |
| Figure S3a | ATR-IR spectrum of **2** | S6 |
| Figure S3b | IR spectrum of **2** | S6 |
| Figure S4 / Table S1 | UV–vis spectrum of **2** / UV–vis data of **2** | S7 |
| Figure S5 | $^{1}$H NMR spectrum of **2a** | S8 |
| Figure S6 | ESI-MS spectrum of **2a** | S9 |
| Figure S7a | ATR-IR spectrum of **2a** | S10 |
| Figure S7b | IR spectrum of **2a** | S10 |
| Figure S8 /Table S2 | UV–vis spectrum of **2a** / UV–vis data of **2a** | S11 |
| Figure S9 | ESI-MS spectrum of **2b** | S12 |
| Figure S10a | ATR-IR spectrum of **2b** | S13 |
| Figure S10b | IR spectrum of **2b** | S13 |
| Figure S11 / Table S3 | UV–vis spectrum of **2b** / UV–vis data of **2b** | S14 |
| Figure S12 | $^{1}$H and $^{13}$C{$^{1}$H} NMR spectra of **2c** | S15 |
| Figure S13 | ESI-MS spectrum of **2c** | S16 |
| Figure S14a | ATR-IR spectrum of **2c** | S17 |
| Figure S14b | IR spectrum of **2c** | S17 |
| Figure S15 / Table S4 | UV–vis spectrum of **2c** / UV–vis data of **2c** | S18 |
| Figure S16 | ESI-MS spectrum of **2d** | S19 |
| Figure S17a | ATR-IR spectrum of **2d** | S20 |
| Figure S17b | IR spectrum of **2d** | S20 |
| Figure S18 / Table S5 | UV–vis spectrum of **2d** / UV–vis data of **2d** | S21 |
| Figure S19 | $^{1}$H and $^{13}$C{$^{1}$H} NMR spectra of **3** | S22 |
| Figure S20 | ESI-MS spectrum of **3** | S23 |
| Figure S21a | ATR-IR spectrum of **3** | S24 |
| Figure S21b | IR spectrum of **3** | S24 |
| Figure S22 / Table S6 | UV–vis spectrum of **3** / UV–vis data of **3** | S25 |
| Figure S23 | $^{1}$H and $^{13}$C{$^{1}$H} NMR spectra of **3a** | S26 |
| Figure S24 | ESI-MS spectrum of **3a** | S27 |
| Figure S25a | ATR-IR spectrum of **3a** | S28 |
| Figure S25b | IR spectrum of **3a** | S28 |
| Figure S26 / Table S7 | UV–vis spectrum of **3a** / UV–vis data of **3a** | S29 |

| | | |
|---|---|---|
| Figure S27 | ESI-MS spectrum of **3b** | S30 |
| Figure S28a | ATR-IR spectrum of **3b** | S31 |
| Figure S28b | IR spectrum of **3b** | S31 |
| Figure S29 / Table S8 | UV–vis spectrum of **3b** / UV–vis data of **3b** | S32 |
| Figure S30 | $^{1}$H and $^{13}$C{$^{1}$H} NMR spectra of **3c** | S33 |
| Figure S31 | ESI-MS spectrum of **3c** | S34 |
| Figure S32a | ATR-IR spectrum of **3c** | S35 |
| Figure S32b | IR spectrum of **3c** | S35 |
| Figure S33 / Table S9 | UV–vis spectrum of **3c** / UV–vis data of **3c** | S36 |
| Figure S34 | ESI-MS spectrum of **3d** | S37 |
| Figure S35a | ATR-IR spectrum of **3d** | S38 |
| Figure S35b | IR spectrum of **3d** | S38 |
| Figure S36 / Table S10 | UV–vis spectrum of **3d** / UV–vis data of **3d** | S39 |
| Figure S37 | IR spectra of **3b** and dark material incl. optical photographs. | S40 |
| Figure S38 | IR spectra of **3d** and dark material incl. optical photographs. | S41 |

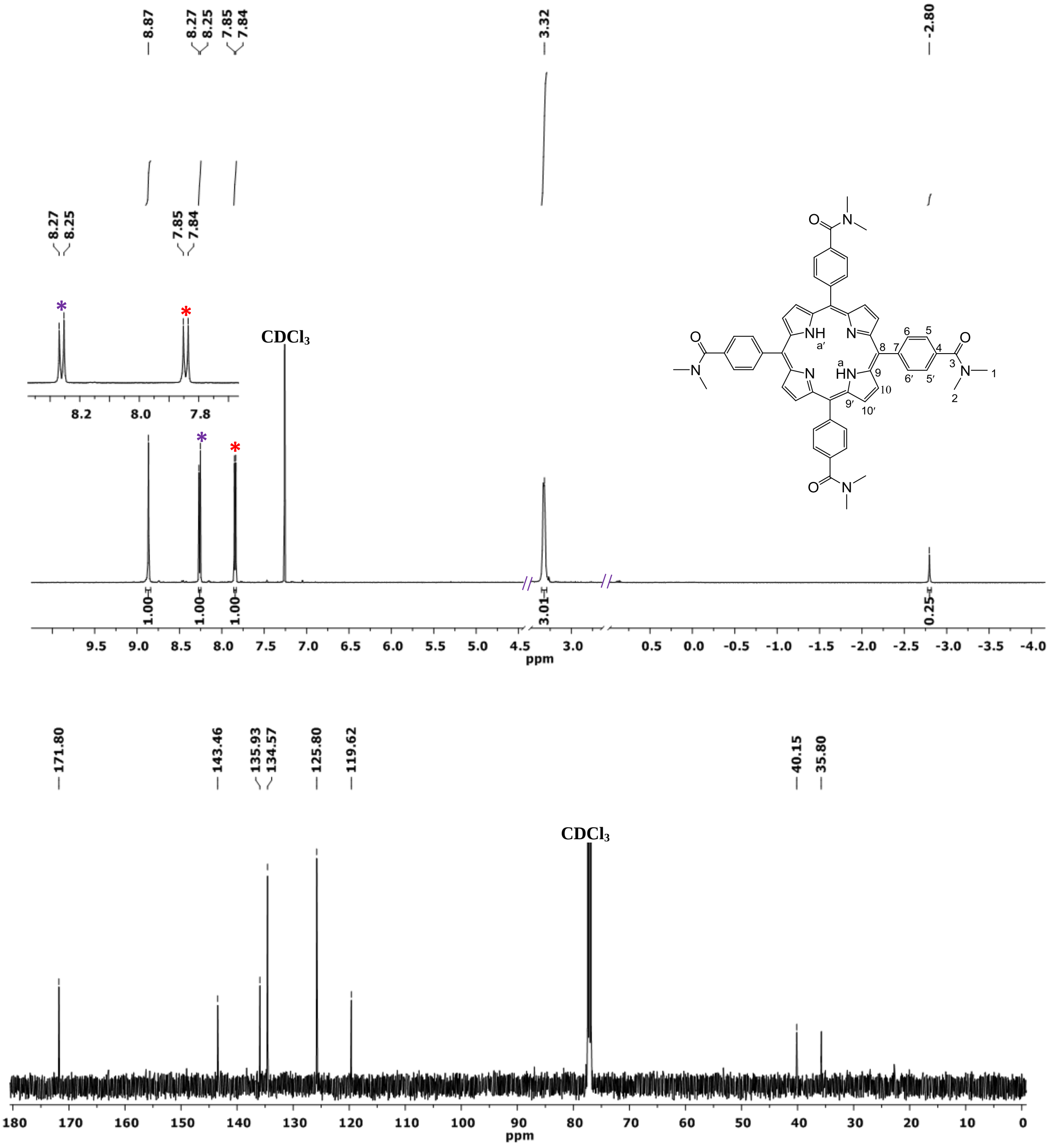


**Figure S1:** $^{1}H$ (above) and $^{13}C\{^{1}H\}$ NMR spectra (below) of **2**.

According to Jones and Wilkins [S1] for the -$NMe_2$ groups two $^{13}C$ NMR chemical shifts are observed. According to Manke et al. [S2] the $^{13}C$ NMR resonances of the pyrrole carbon atoms $C^{9,9'}$ and $C^{10,10'}$ are not observable.

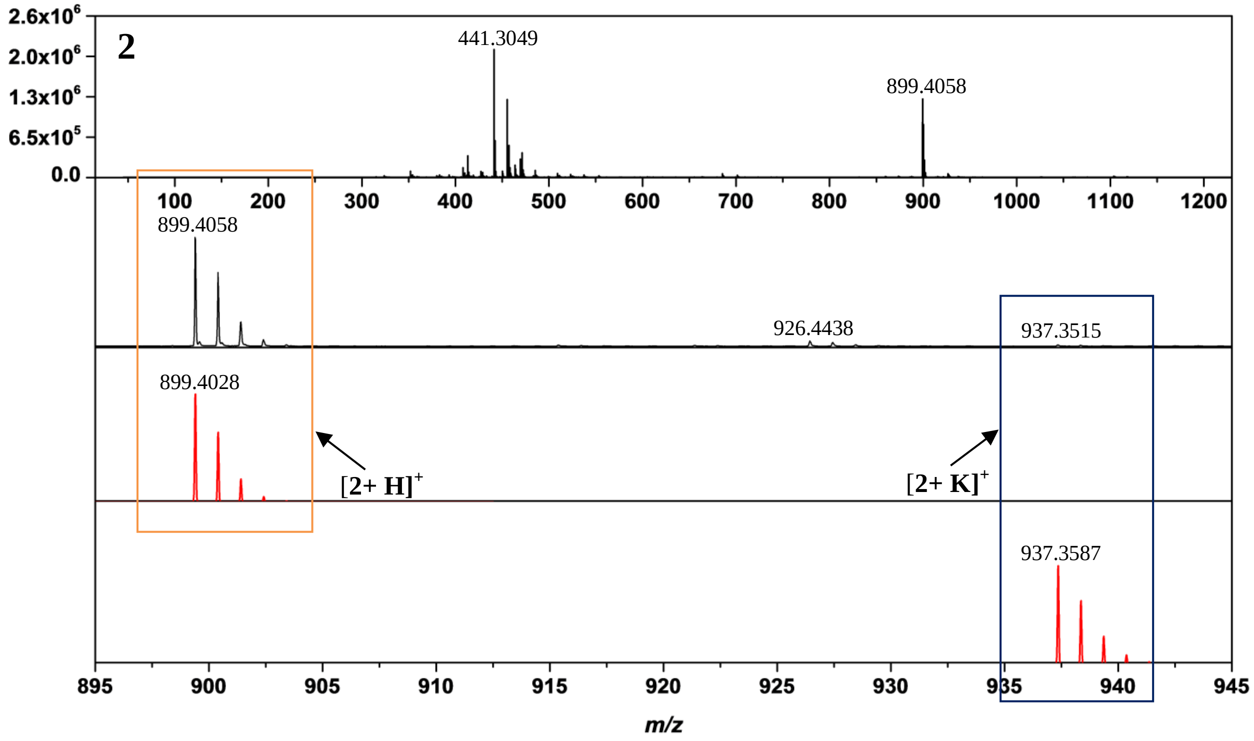


**Figure S2:** ESI-MS spectrum of **2**.

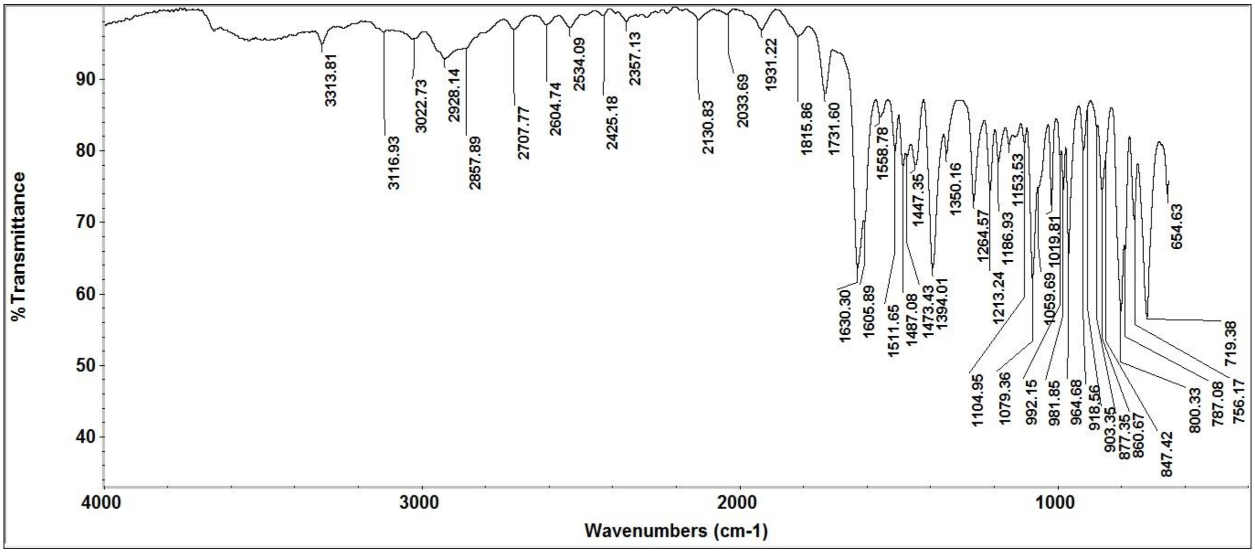


**Figure S3a:** IR spectrum (ATR-IR) of **2**.

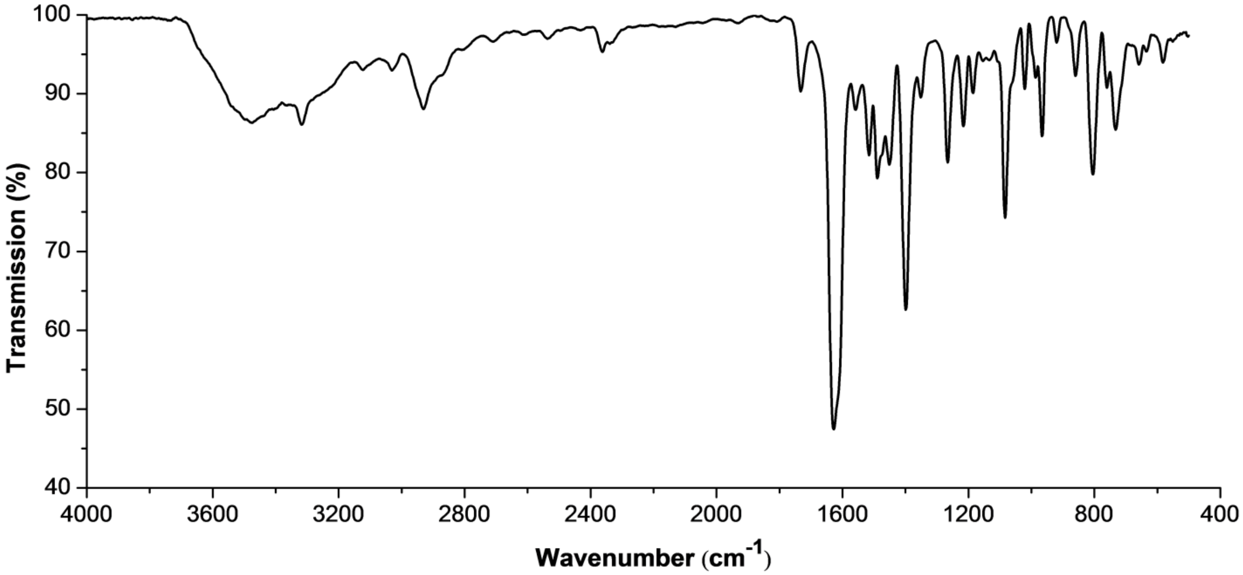


**Figure S3b:** IR spectrum (KBr) of **2**.

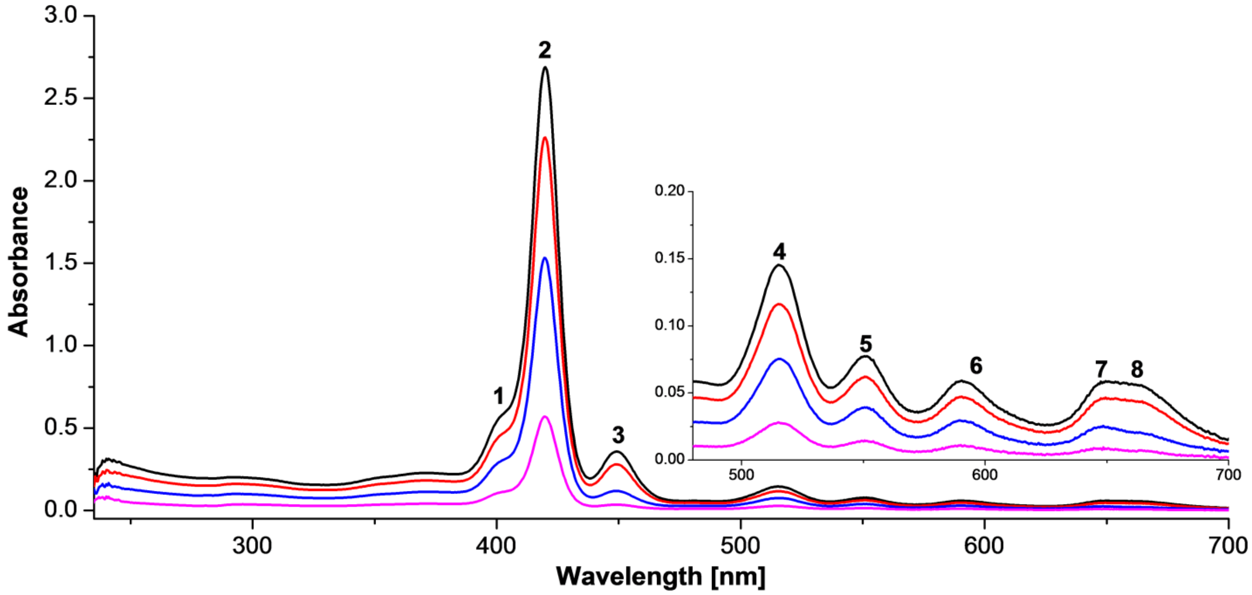


**Figure S4:** UV–vis spectrum of **2** in $CHCl_3$ at different concentrations.

**Table S1:** UV–vis data ($\lambda_{max}$ [nm] log (ε [L·mol$^{-1}$·cm$^{-1}$])) of **2** at different concentrations.

| | **Absorption $\lambda_{max}$ [nm] log (ε [L·mol$^{-1}$·cm$^{-1}$])** | | | | | | | |
|---|---|---|---|---|---|---|---|---|
| | **1** | **2** | **3** | **4** | **5** | **6** | **7** | **8** |
| ***C*$_1$ = 8.620·10$^{-6}$ mol/L** | 400.8 (4.81) | 419.8 (5.49) | 449.3 (4.63) | 515.6 (4.23) | 550.6 (3.951) | 590.8 (3.84) | 647 (3.83) | 665.5 (3.81) |
| ***C*$_2$ = 5.172·10$^{-6}$ mol/L** | 400.8 (4.93) | 419.8 (5.64) | 449.3 (4.74) | 515.6 (4.35) | 550.6 (4.08) | 590.8 (3.96) | 647 (3.93) | 665.5 (3.93) |
| ***C*$_3$ = 1.724·10$^{-6}$ mol/L** | 400.8 (5.24) | 419.8 (5.95) | 449.3 (4.83) | 515.6 (4.64) | 550.6 (4.36) | 590.8 (3.24) | 647 (4.17) | 666.3 (4.07) |
| ***C*$_4$ = 3.448·10$^{-6}$ mol/L** | 400.8 (4.49) | 419.8 (5.22) | 449.3 (4.05) | 515.6 (3.90) | 550.6 (3.64) | 590.8 (3.51) | 647 (3.41) | 666.3 (3.41) |

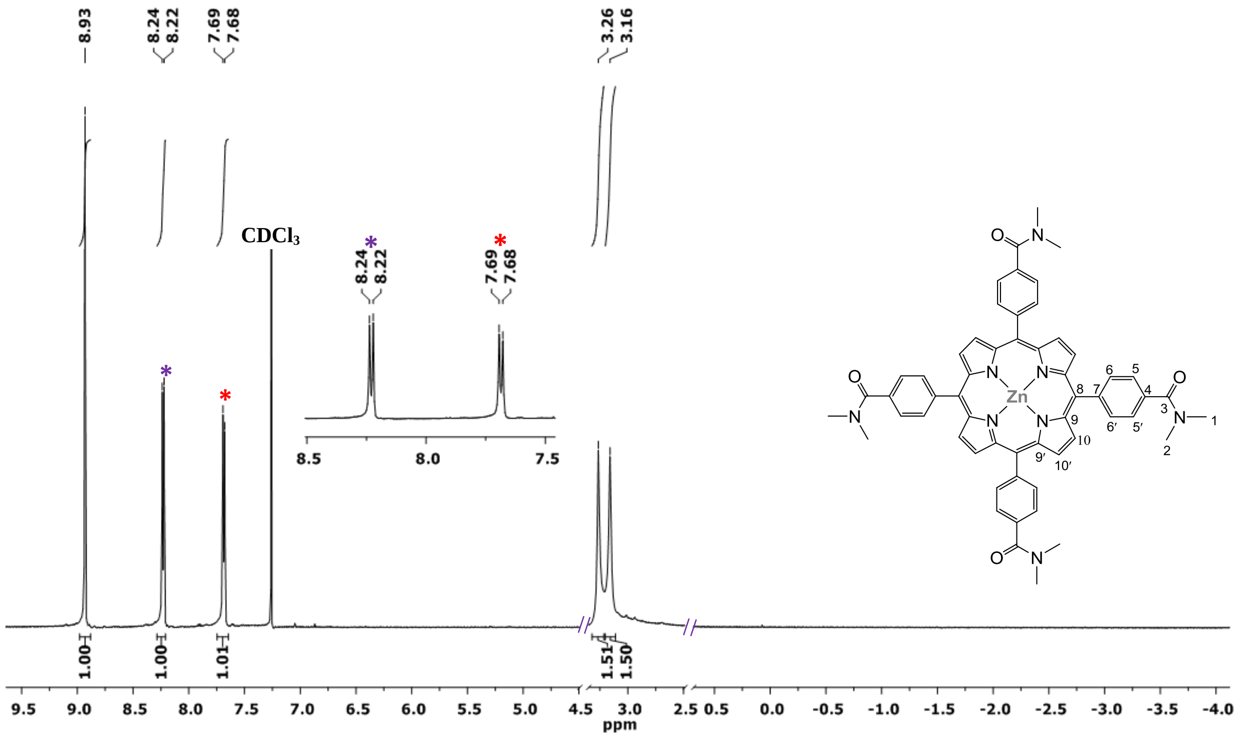


**Figure S5:** $^1$H NMR spectrum of **2a**.

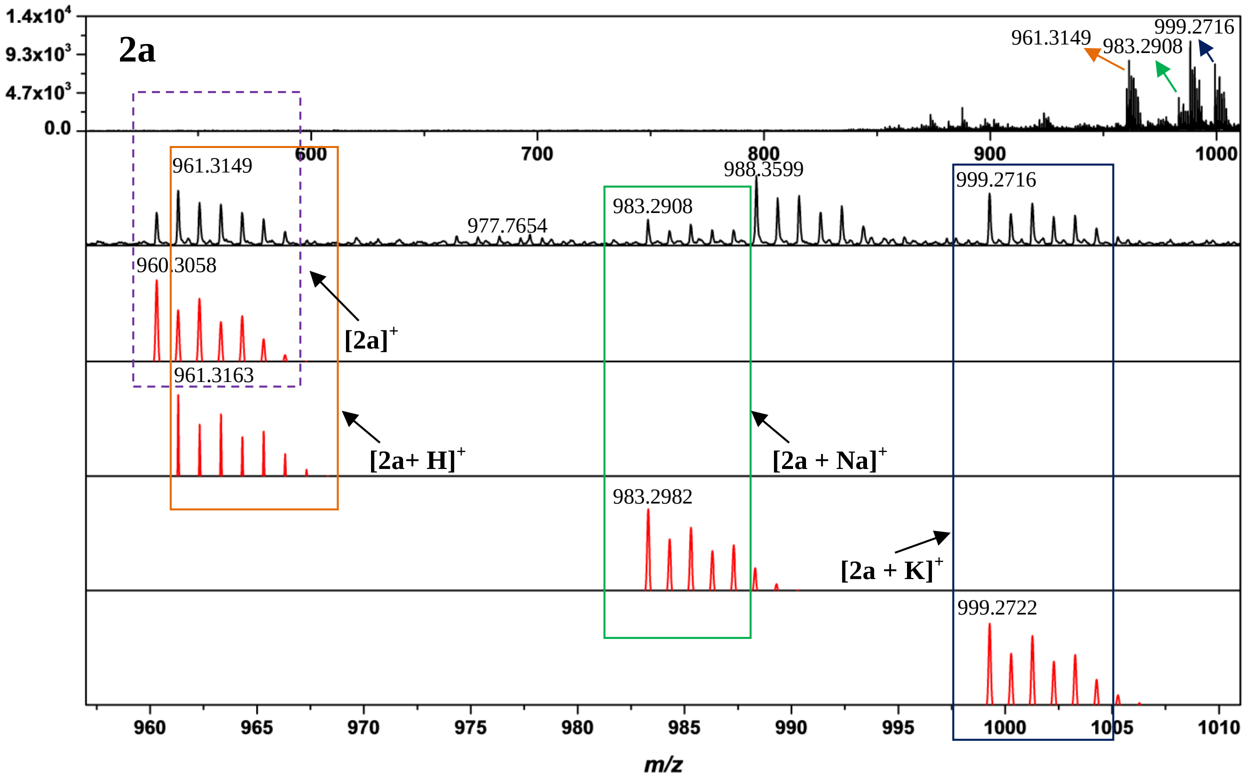


**Figure S6:** ESI-MS spectrum of **2a** (Black: Measured. Red: Calculated).

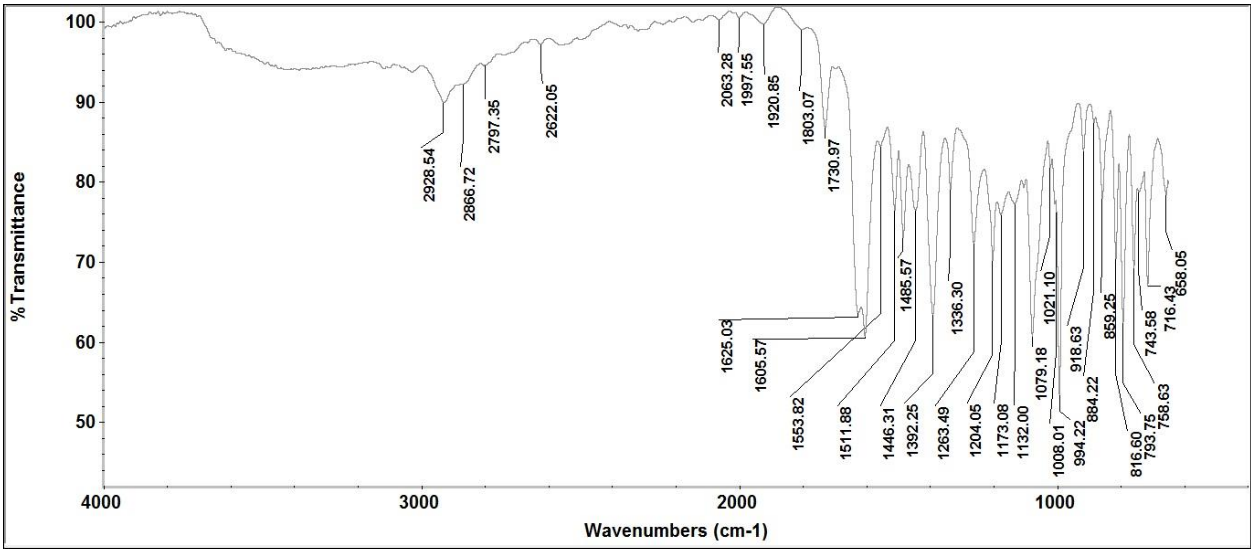


**Figure S7a:** IR spectrum (ATR-IR) of **2a**.

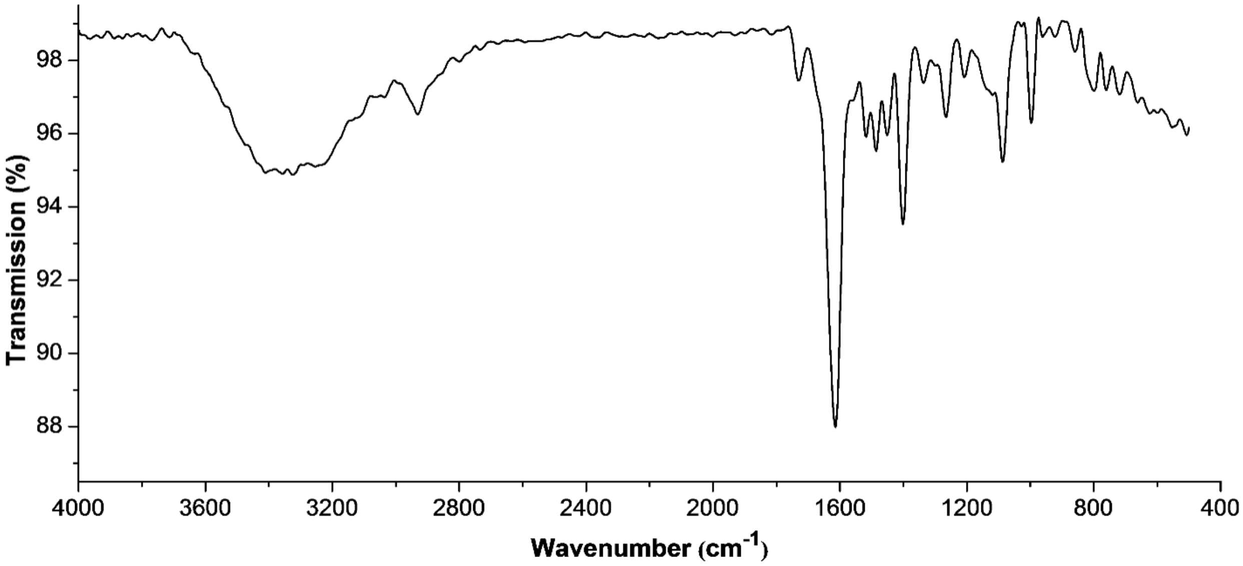


**Figure S7b:** IR spectrum (KBr) of **2a**.

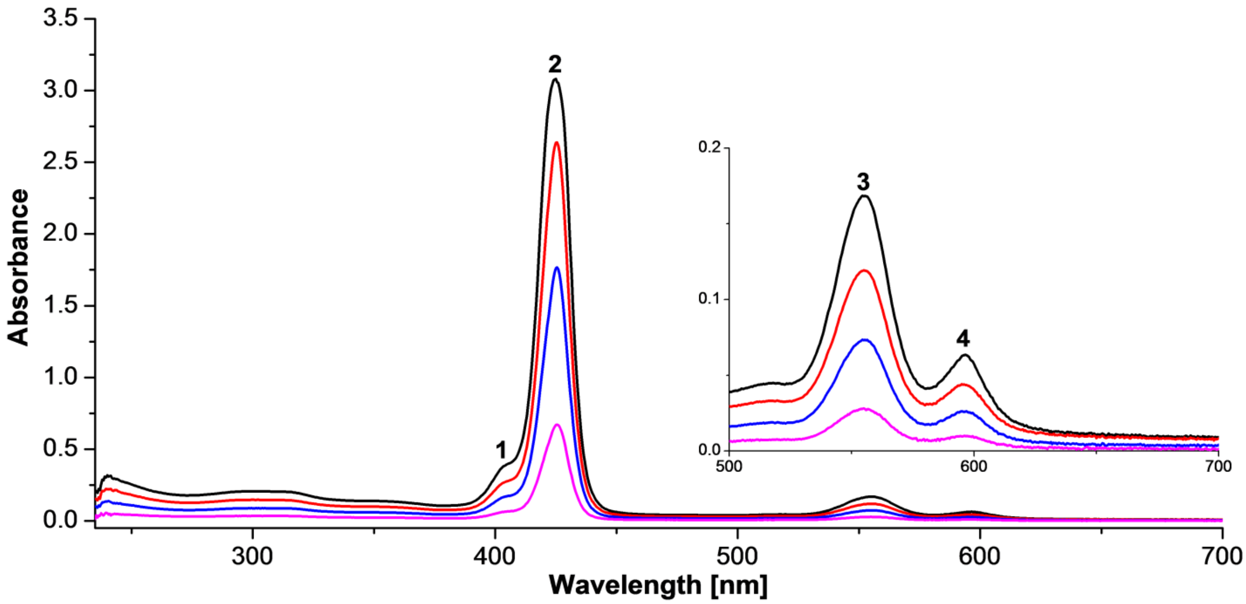


**Figure S8:** UV–vis spectrum of **2a** in $CHCl_3$ at different concentrations.

**Table S2:** UV–vis data ($\lambda_{max}$ [nm] log (ε [L·mol$^{-1}$·cm$^{-1}$])) of **2a** at different concentrations.

| | **Absorption $\lambda_{max}$ [nm] log (ε [L·mol$^{-1}$·cm$^{-1}$])** | | | |
|---|---|---|---|---|
| | **1** | **2** | **3** | **4** |
| **$C_1$ = 1.127·10$^{-5}$ mol/L** | 403.4 (4.54) | 425.1 (5.44) | 555.1 (4.18) | 596.3 (3.75) |
| **$C_2$ = 8.053·10$^{-6}$ mol/L** | 403.4 (4.54) | 425.1 (5.51) | 555.1 (4.17) | 596.3 (3.74) |
| **$C_3$ = 4.832·10$^{-6}$ mol/L** | 403.4 (4.55) | 425.6 (5.55) | 555.1 (4.19) | 596.3 (3.73) |
| **$C_4$ = 1.611·10$^{-6}$ mol/L** | 403.4 (4.60) | 425.6 (5.62) | 555.1 (4.22) | 596.3 (3.77) |

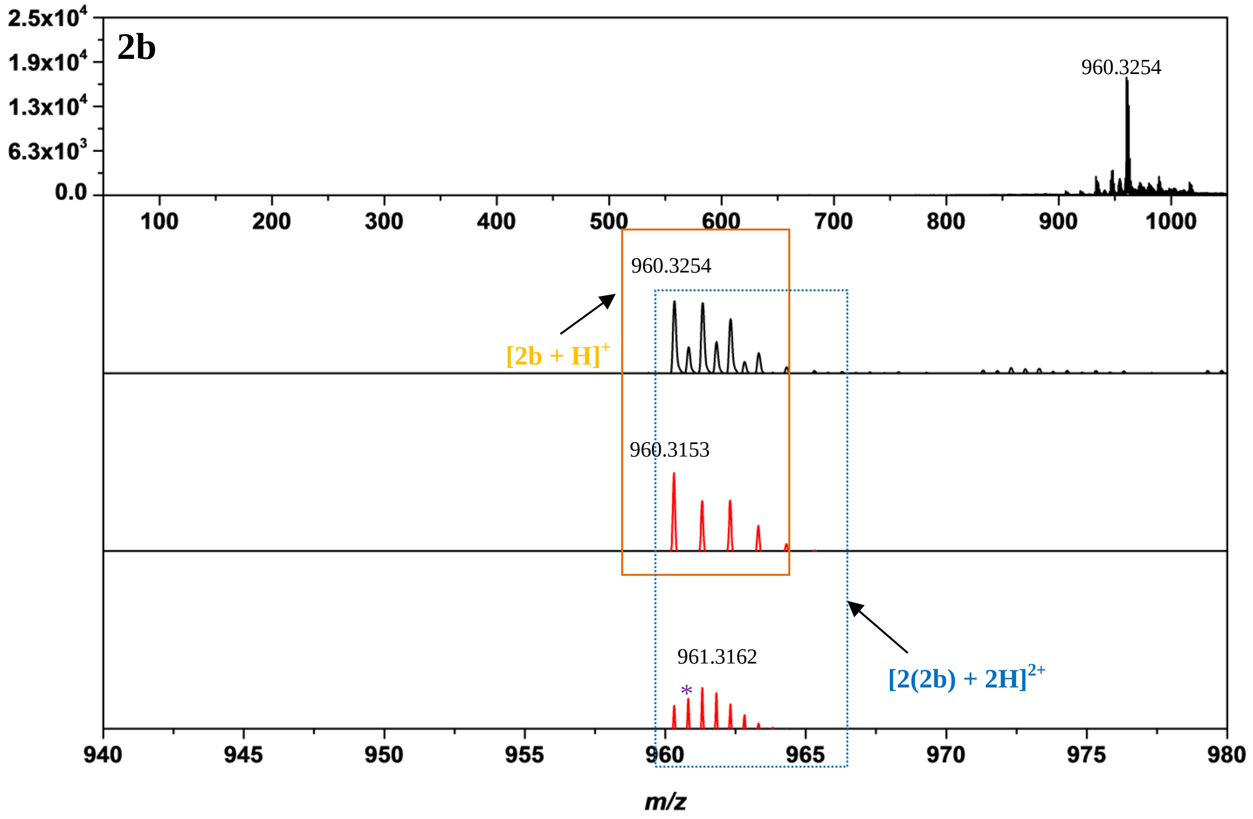


**Figure S9:** ESI-MS spectrum of **2b** (Black: Measured. Red: Calculated).

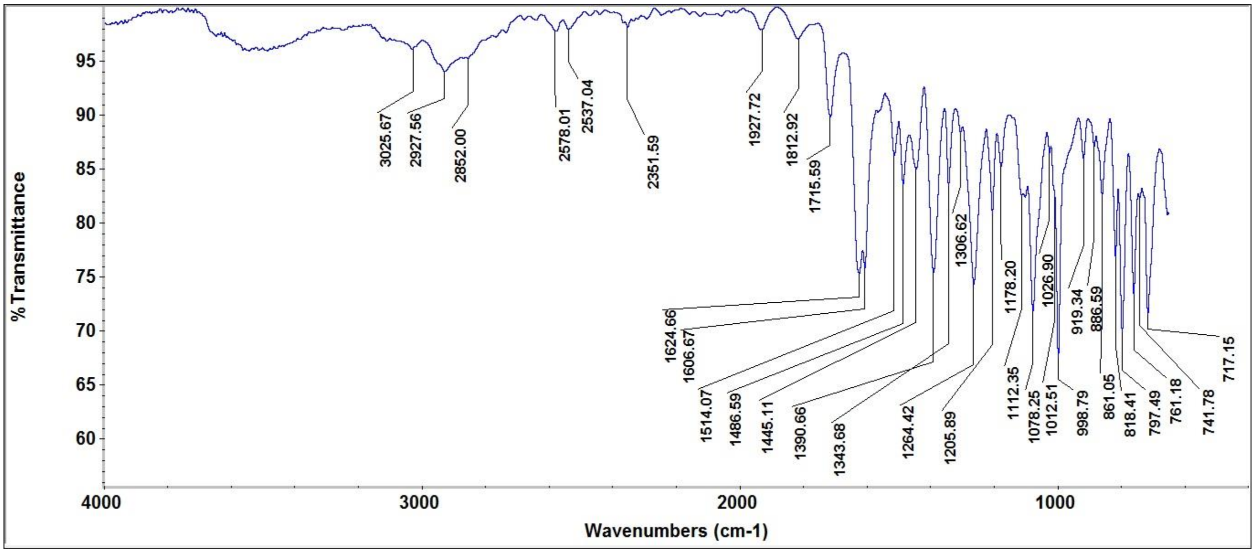


**Figure S10a:** IR spectrum (ATR-IR) of **2b**.

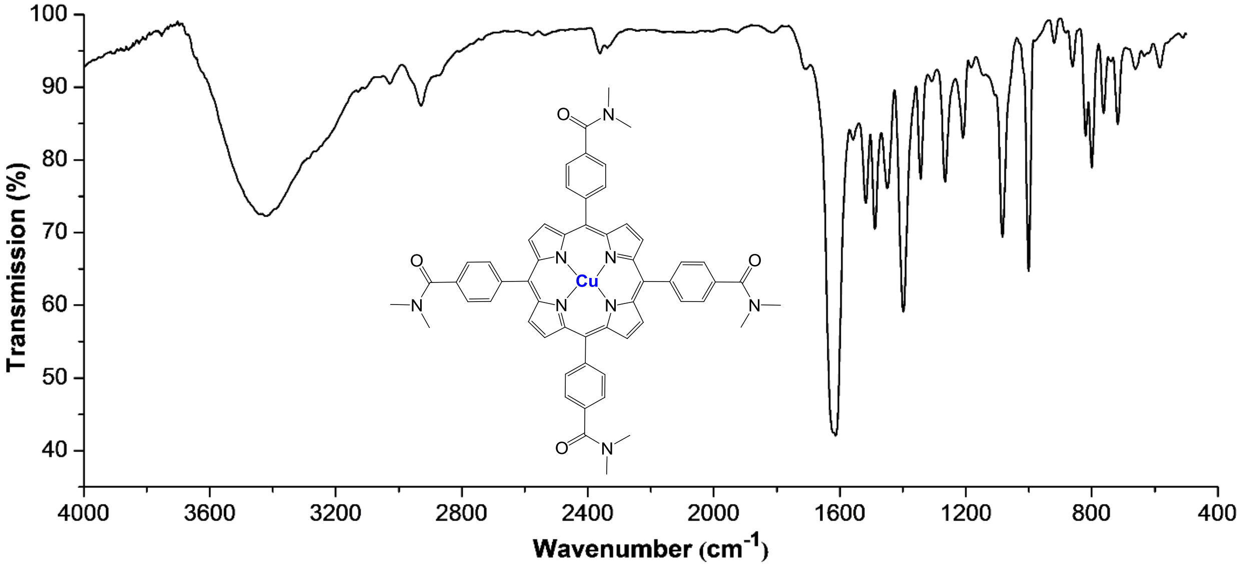


**Figure S10b:** IR spectrum (KBr) of **2b**.

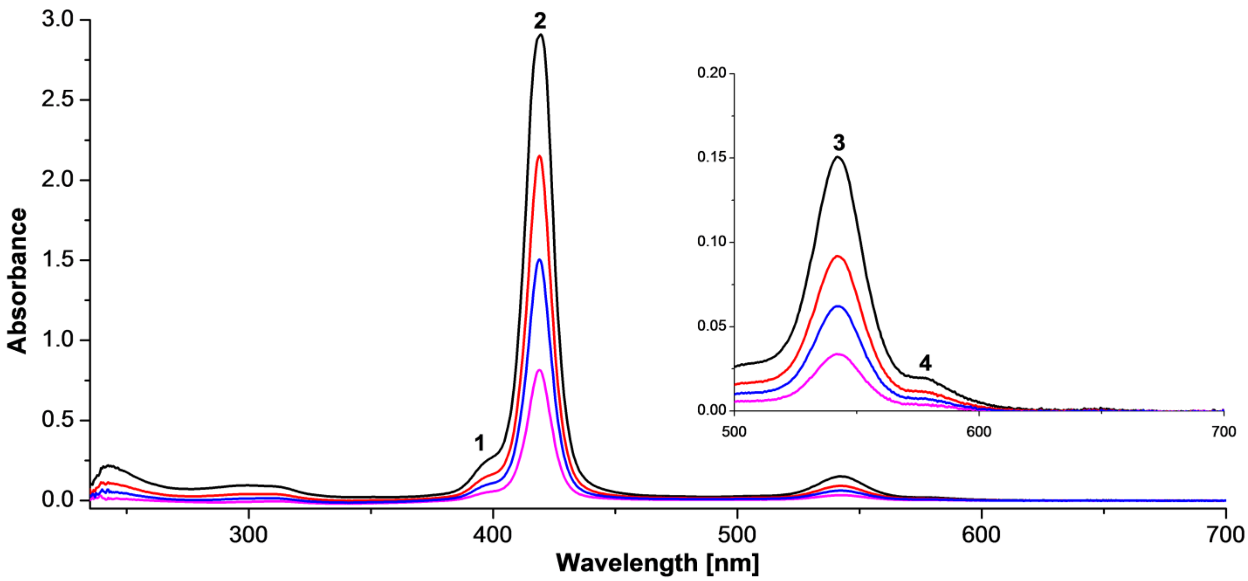


**Figure S11:** UV–vis spectrum of **2b** in $CHCl_3$ at different concentrations.

**Table S3:** UV–vis data ($\lambda_{max}$ [nm] log (ε [L·mol$^{-1}$·cm$^{-1}$])) of **2b** at different concentrations.

| | **Absorption $\lambda_{max}$ [nm] log (ε [L·mol$^{-1}$·cm$^{-1}$])** | | | |
|---|---|---|---|---|
| | **1** | **2** | **3** | **4** |
| **$C_1$ = 8.328·10$^{-6}$ mol/L** | 396 (4.48) | 419.2 (5.54) | 542.7 (4.26) | 578.4 (3.38) |
| **$C_2$ = 4.997·10$^{-6}$ mol/L** | 396 (4.47) | 419.2 (5.63) | 542.7 (4.27) | 578.4 (3.40) |
| **$C_3$ = 3.331·10$^{-6}$ mol/L** | 396 (4.44) | 419.2 (5.65) | 542.7 (4.28) | 578.4 (3.39) |
| **$C_4$ = 1.661·10$^{-6}$ mol/L** | 396 (4.45) | 419.2 (5.68) | 542.7 (4.31) | 578.4 (3.37) |

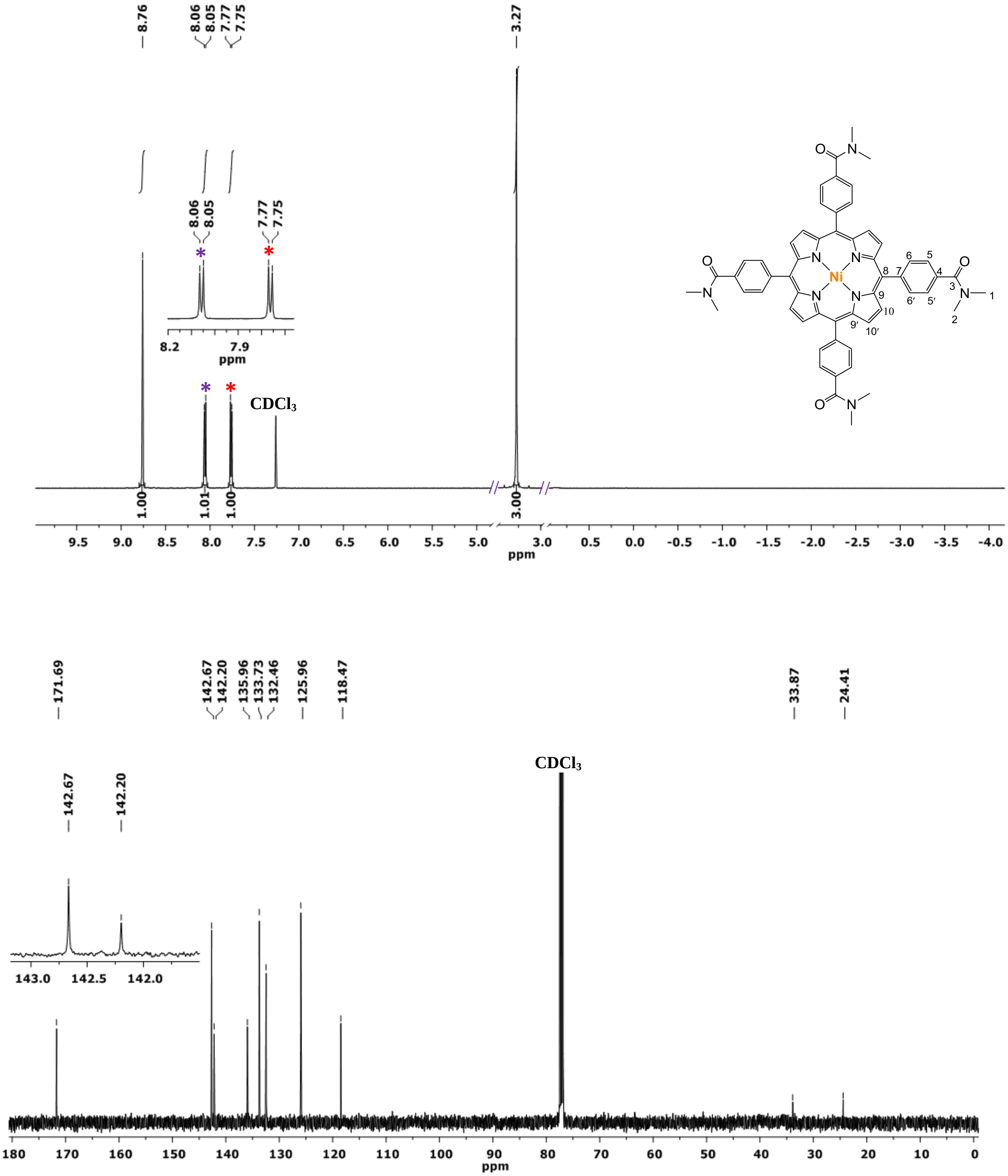


**Figure S12:** $^{1}H$ (above) and $^{13}C\{^{1}H\}$ NMR spectra (below) of **2c**.

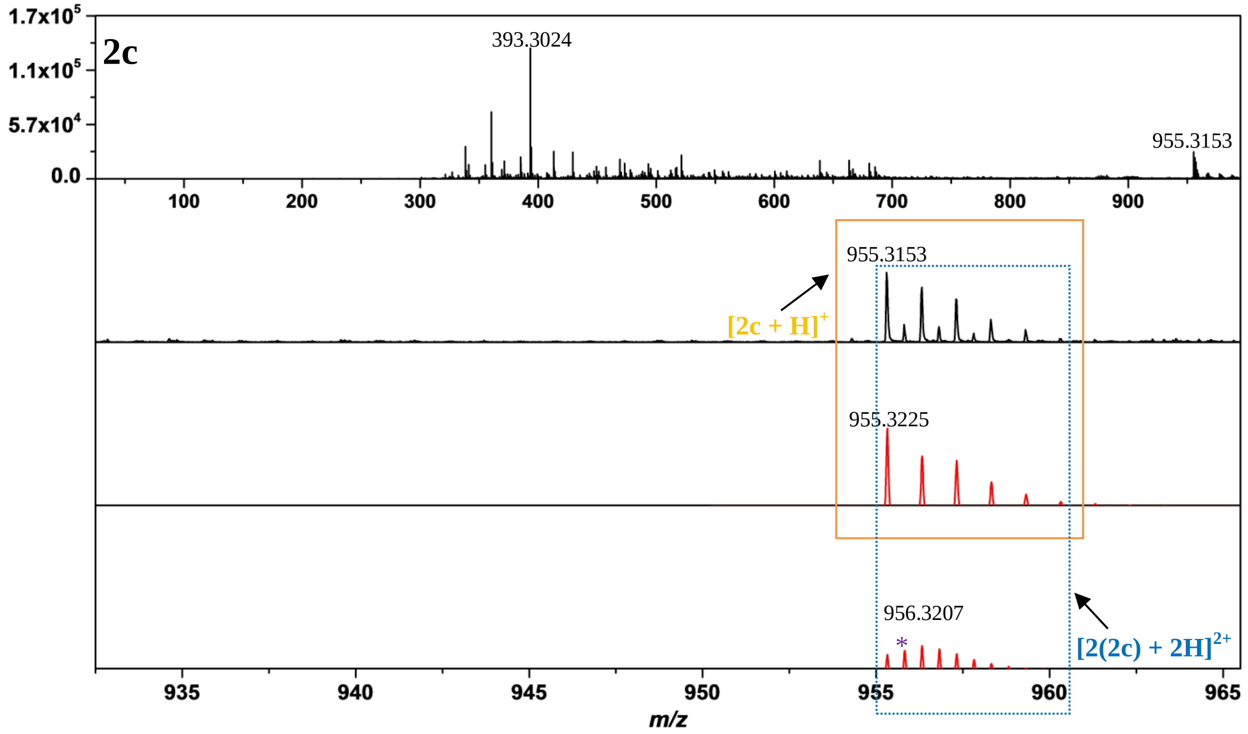


**Figure S13:** ESI-MS spectrum of **2c** (Black: Measured. Red: Calculated).

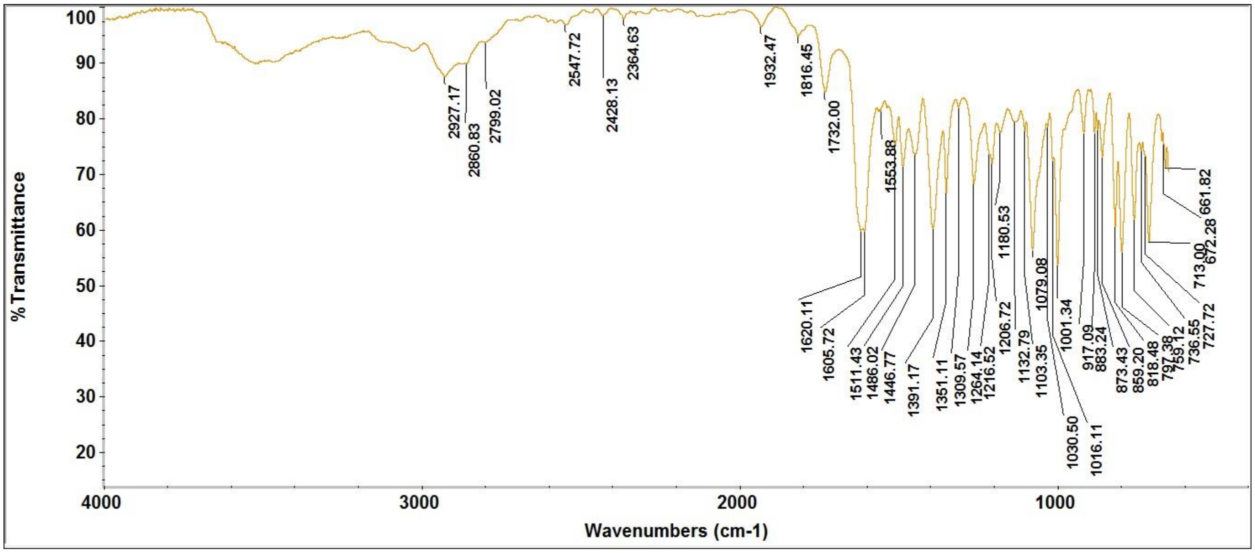


**Figure S14a:** IR spectrum (ATR-IR) of **2c**.

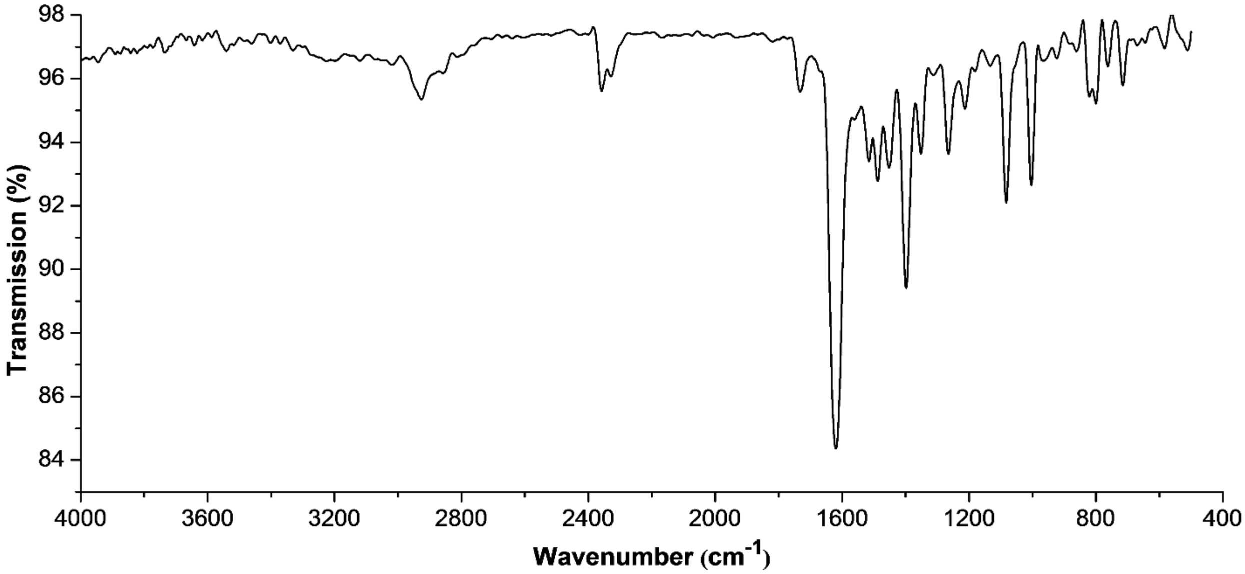


**Figure S14b:** IR spectrum (KBr) of **2c**.

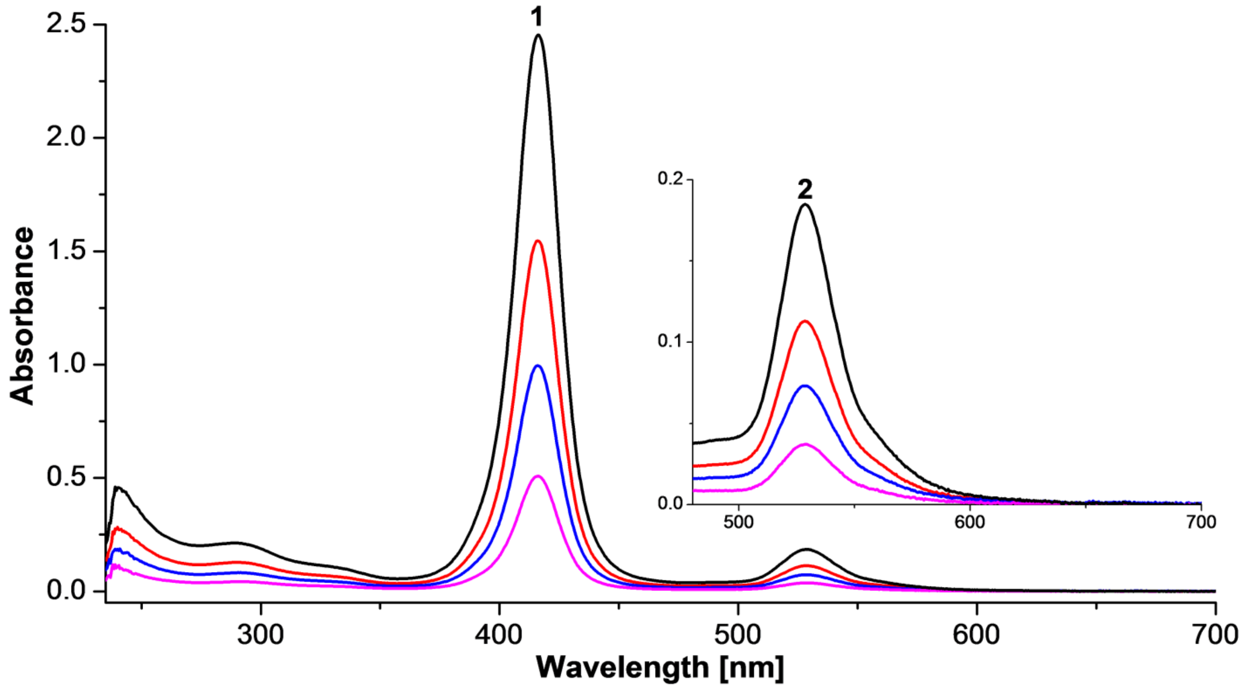


**Figure S15:** UV–vis spectrum of **2c** in $CHCl_3$ at different concentrations.

**Table S4:** UV–vis data ($\lambda_{max}$ [nm] log (ε [L·mol$^{-1}$·cm$^{-1}$])) of **2c** at different concentrations.

| | **Absorption $\lambda_{max}$ [nm] log (ε [L·mol$^{-1}$·cm$^{-1}$])** | |
|---|---|---|
| | **1** | **2** |
| ***C*$_1$ = 1.282·10$^{-5}$ mol/L** | 416.2 (5.28) | 528.2 (4.16) |
| ***C*$_2$ = 7.691·10$^{-6}$ mol/L** | 416.2 (5.30) | 528.2 (4.17) |
| ***C*$_3$ = 5.127·10$^{-6}$ mol/L** | 416.2 (5.29) | 528.2 (4.16) |
| ***C*$_4$ = 2.564·10$^{-6}$ mol/L** | 416.2 (5.30) | 528.2 (4.16) |

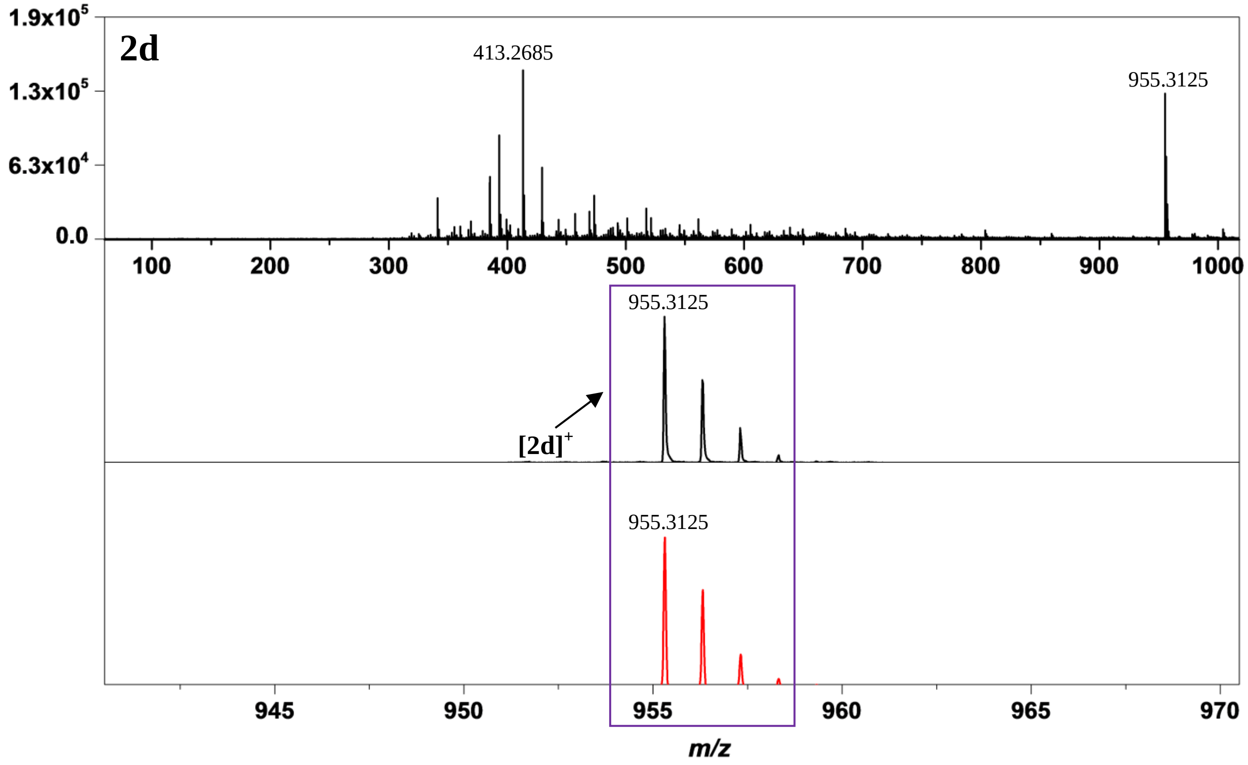


**Figure S16:** ESI-MS spectrum of **2d** (Black: Measured. Red: Calculated).

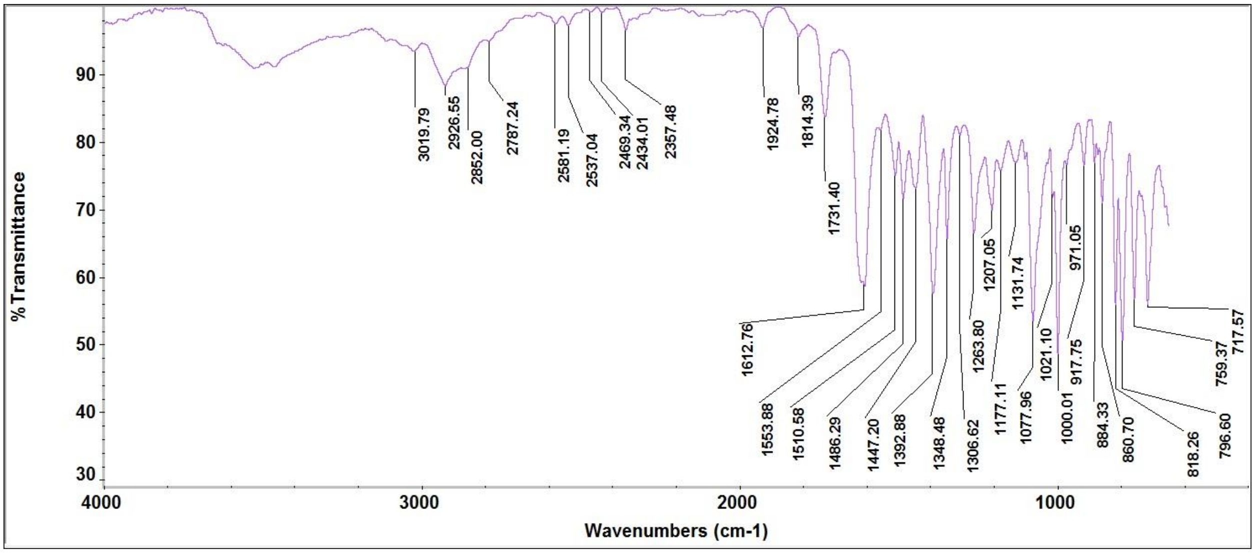


**Figure S17a:** IR spectrum (ATR-IR) of **2d**.

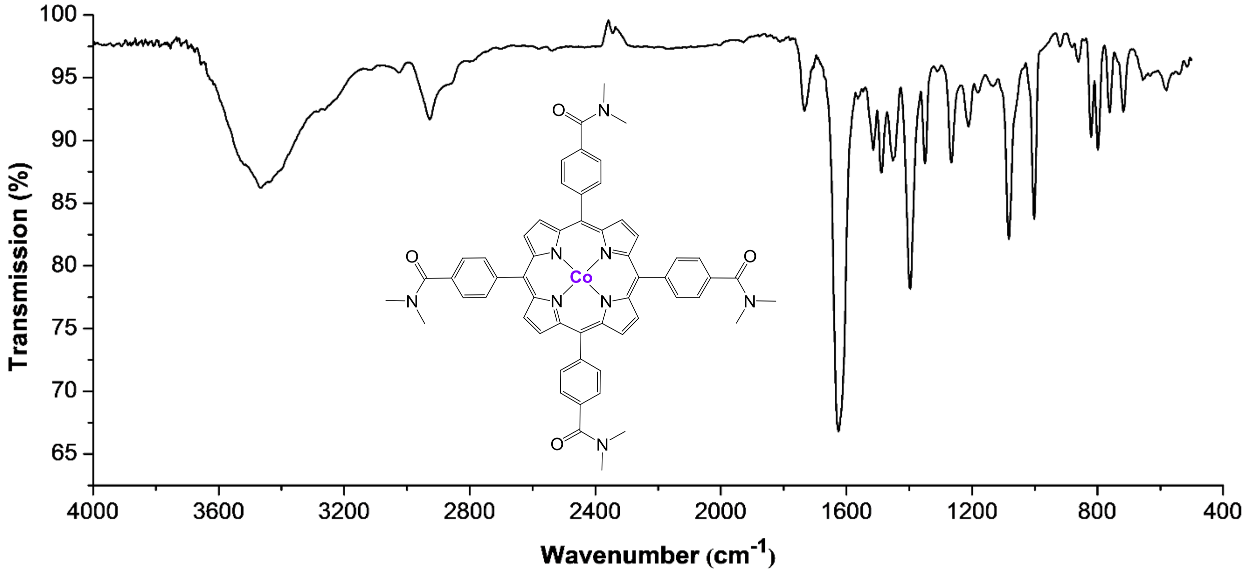


**Figure S17b:** IR spectrum (KBr) of **2d**.

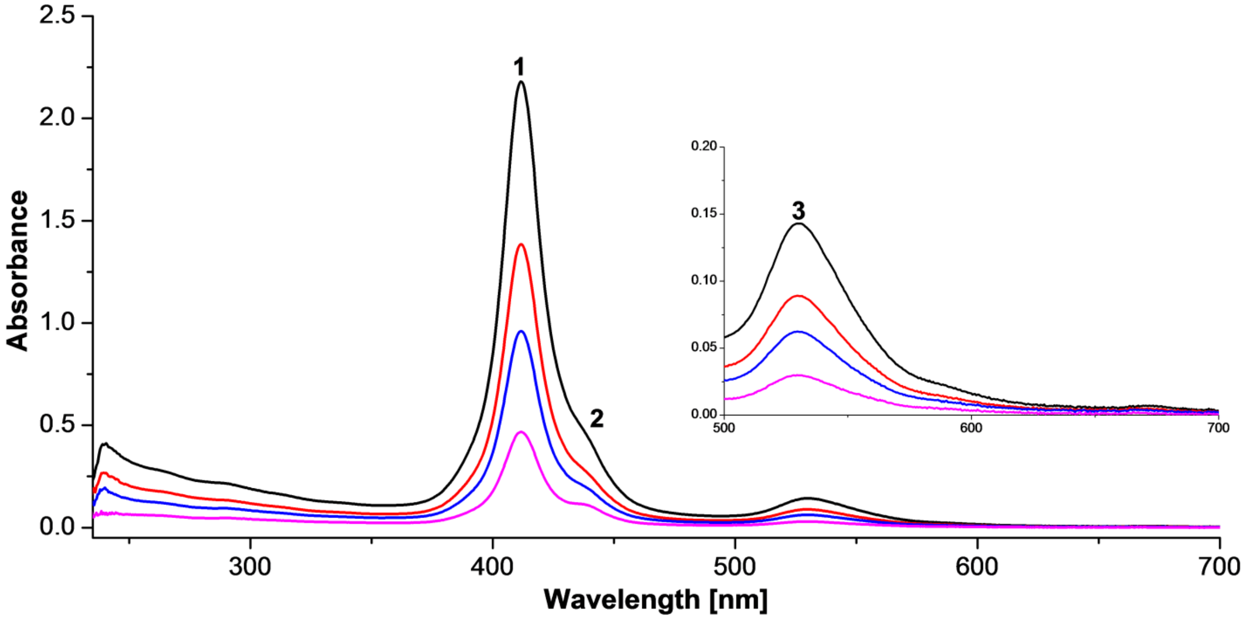


**Figure S18:** UV–vis spectrum of **2d** in $CHCl_3$ at different concentrations.

**Table S5:** UV–vis data ($\lambda_{max}$ [nm] log (ε [L·mol$^{-1}$·cm$^{-1}$])) of **2d** at different concentrations.

| | **Absorption $\lambda_{max}$ [nm] log (ε [L·mol$^{-1}$·cm$^{-1}$])** | | |
|---|---|---|---|
| | **1** | **2** | **3** |
| $\boldsymbol{C_1}$ **= 1.172·10$^{-5}$ mol/L** | 411.9 (5.26) | 442.3 (4.50) | 530 (4.09) |
| $\boldsymbol{C_2}$ **= 8.368·10$^{-6}$ mol/L** | 411.9 (5.24) | 442.3 (4.44) | 530 (4.03) |
| $\boldsymbol{C_3}$ **= 5.021·10$^{-6}$ mol/L** | 411.9 (5.28) | 442.3 (4.53) | 530 (4.10) |
| $\boldsymbol{C_4}$ **= 1.674·10$^{-6}$ mol/L** | 411.9 (5.45) | 441.6 (4.83) | 530 (4.26) |

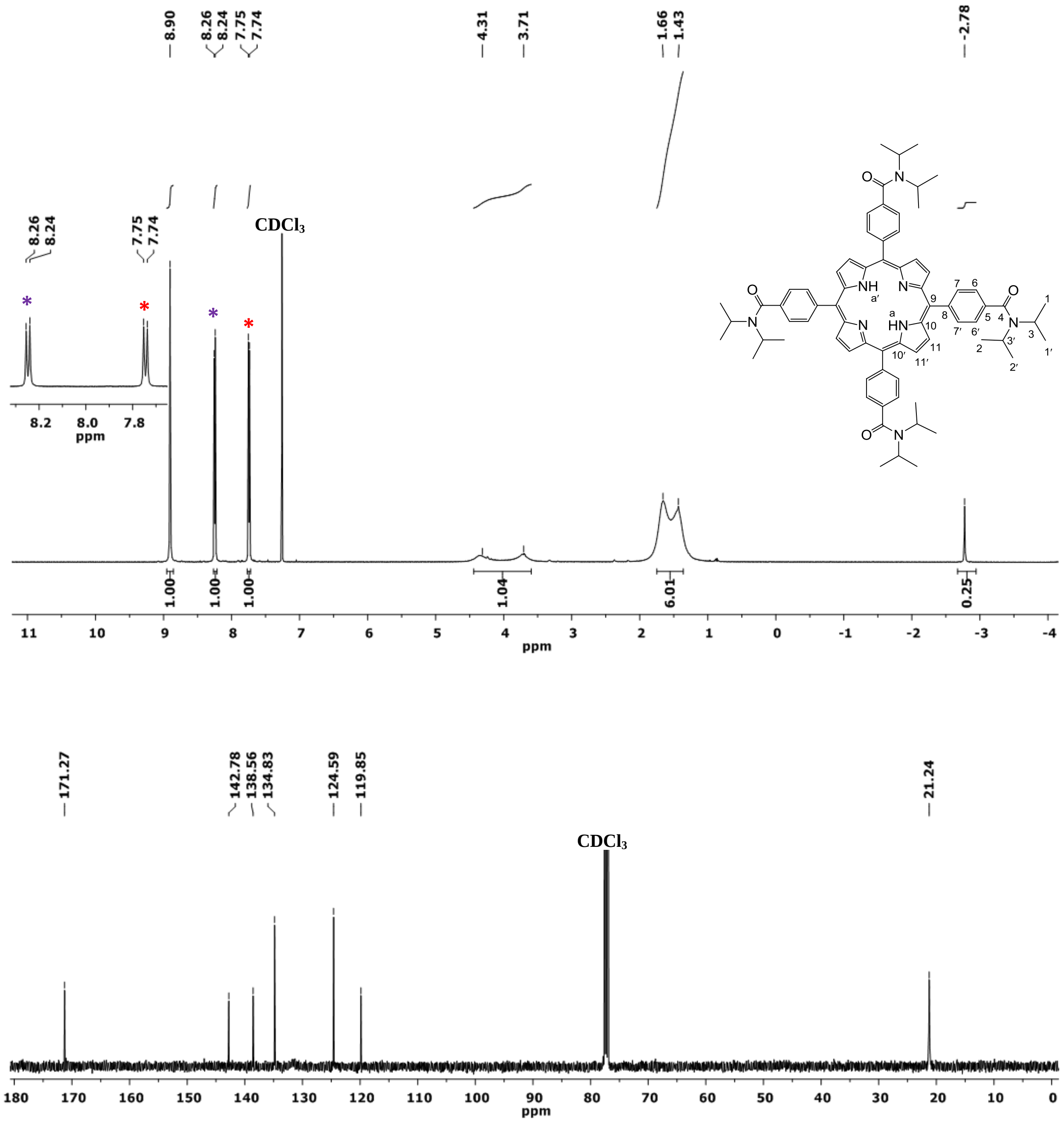


**Figure S19:** $^{1}$H (above) and $^{13}$C{$^{1}$H} NMR spectra (below) of **3**.

The $^{1}$H NMR resonances of the N$^{i}$Pr$_2$ groups are all broadened. The hydrogen atoms H$^{1,1',2,2'}$ are regarded to correspond to the two broad singlets at 1.39 and 1.69 ppm. The hydrogen atoms H$^{3,3'}$ are regarded to correspond to the two singlets at 3.71 and 4.31 ppm. Both assignments could be, however, not verified by additional 2D NMR experiments ($^{1}$H,$^{1}$H-COSY,$^{1}$H,$^{13}$C-HSQCETGP and HMBCGP) which is attributed to too broad NMR resonances and/or to the comparatively poor solubility.

According to Jones and Wilkins [S1] for the -NMe$_2$ groups two $^{13}$C NMR chemical shifts are observed. According to Manke et al. [S2] the $^{13}$C NMR resonances of the pyrrole carbon atoms C$^{9,9'}$ and C$^{10,10'}$ are not observable.

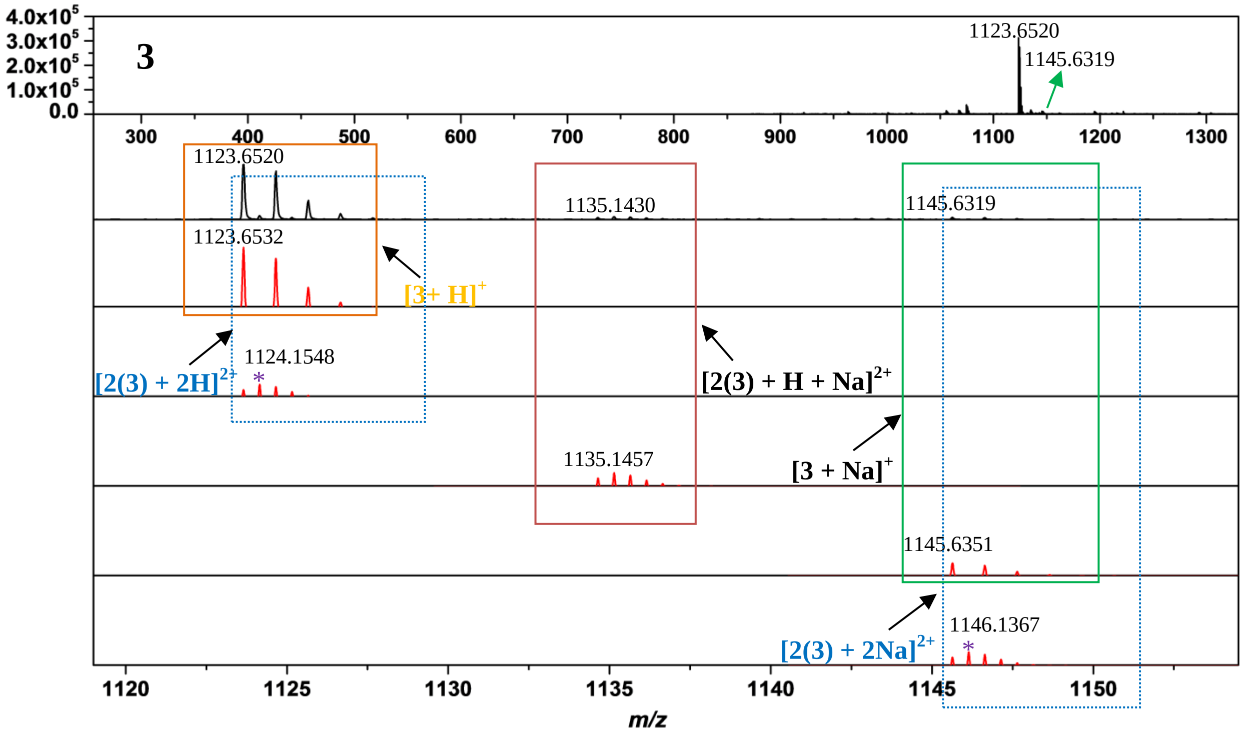


**Figure S20:** ESI-MS spectrum of **3** (Black: Measured. Red: Calculated).

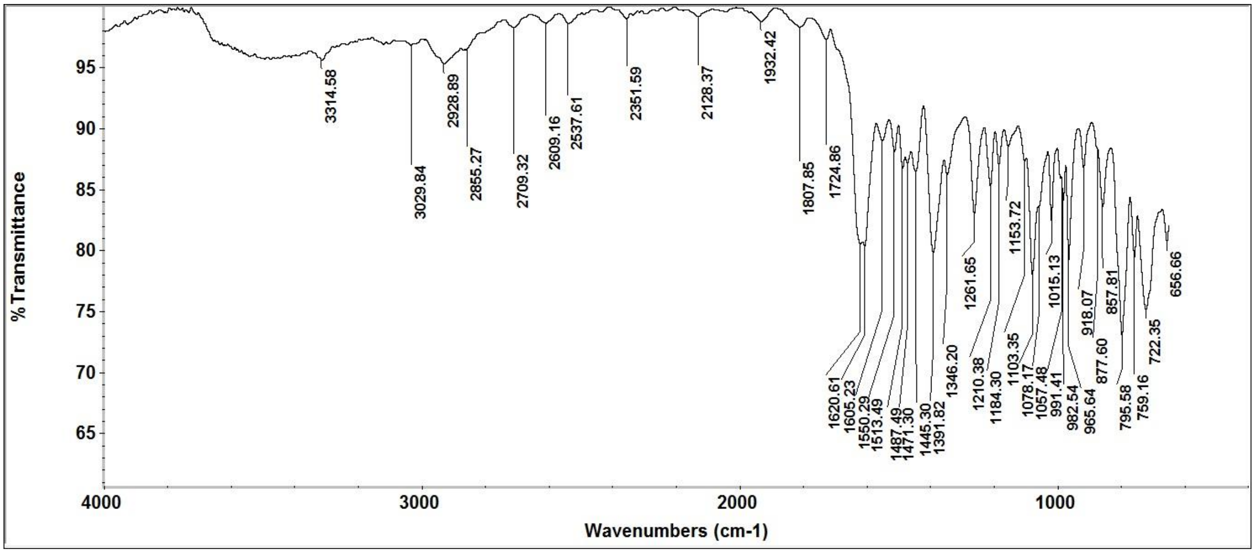


**Figure S21a:** IR spectrum (ATR-IR) of **3**.

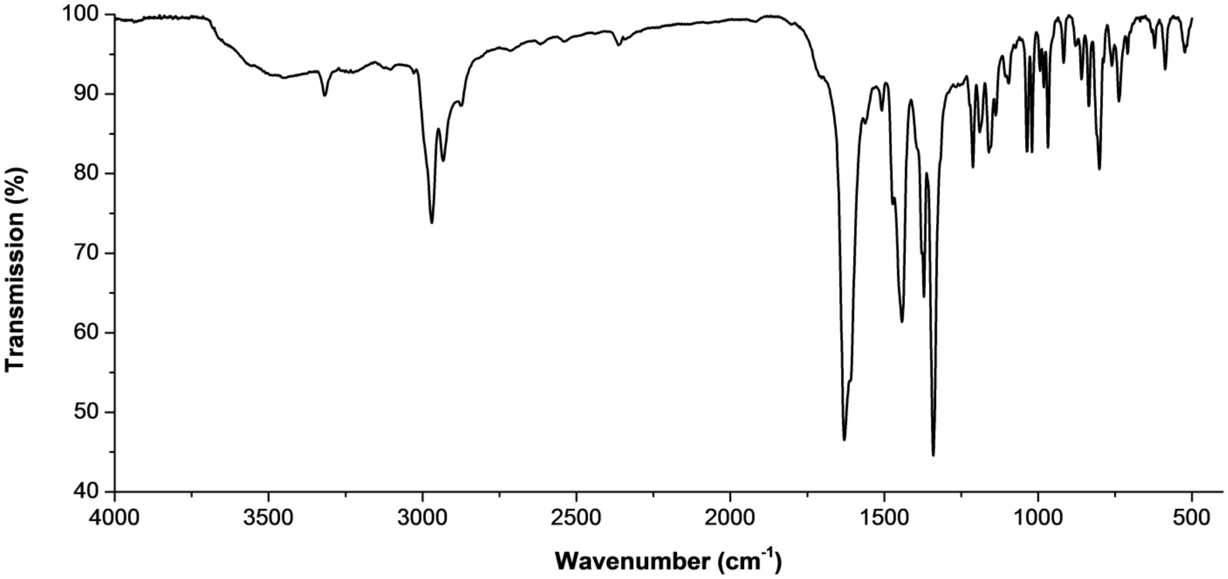


**Figure S21b:** IR spectrum (KBr) of **3**.

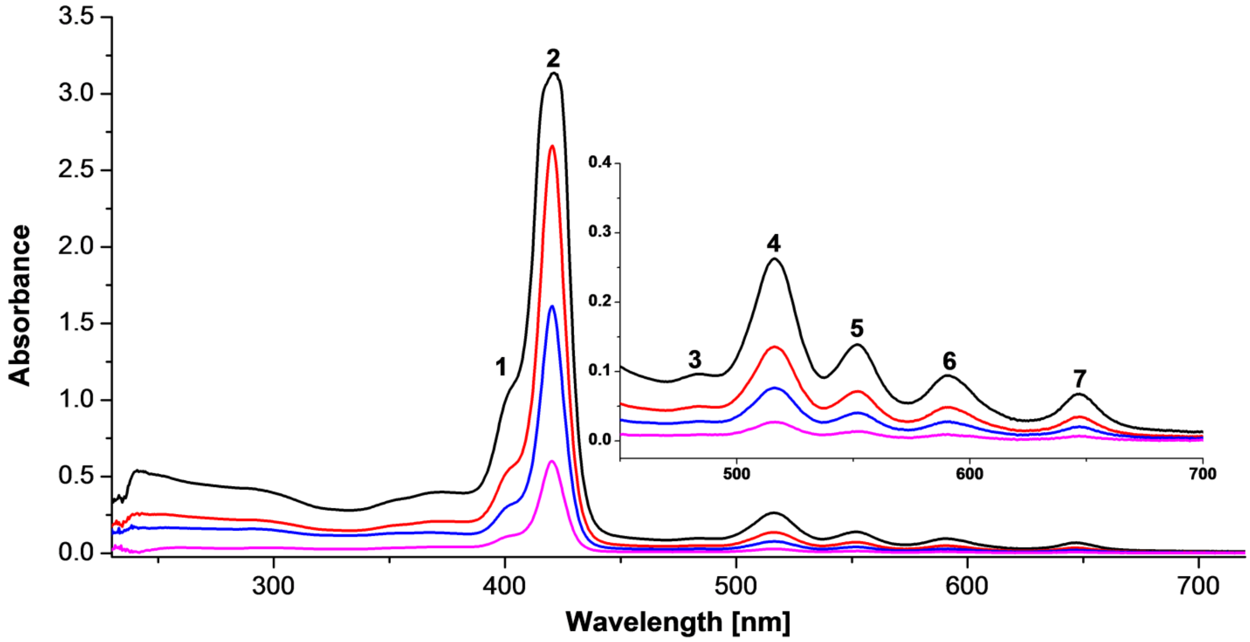


**Figure S22:** UV–vis spectrum of **3** in $CHCl_3$ at different concentrations.

**Table S6:** UV–vis data ($\lambda_{max}$ [nm] log (ε [L·mol$^{-1}$·cm$^{-1}$])) of **3** at different concentrations.

| | **Absorption $\lambda_{max}$ [nm] log (ε [L·mol$^{-1}$·cm$^{-1}$])** | | | | | | |
|---|---|---|---|---|---|---|---|
| | **1** | **2** | **3** | **4** | **5** | **6** | **7** |
| $\boldsymbol{C_1}$ **= 1.736·10$^{-5}$ mol/L** | 399.1 (4.77) | 421.1 (5.26) | 481.7 (3.75) | 516.5 (4.18) | 551.2 (3.91) | 590.7 (3.74) | 647.3 (3.60) |
| $\boldsymbol{C_2}$ **= 1.157·10$^{-5}$ mol/L** | 399.9 (4.66) | 420.8 (5.36) | 481.8 (3.65) | 516.6 (4.07) | 552.1 (3.80) | 590.4 (3.65) | 647.3 (3.51) |
| $\boldsymbol{C_3}$ **= 5.207·10$^{-6}$ mol/L** | 400.1 (4.80) | 420.2 (5.49) | 481.7 (3.74) | 516.5 (4.17) | 552.1 (3.91) | 591.2 (3.74) | 647.8 (3.58) |
| $\boldsymbol{C_4}$ **= 1.736·10$^{-6}$ mol/L** | 400.5 (4.86) | 420.2 (5.54) | 481.8 (3.70) | 516.5 (4.20) | 552.9 (3.97) | 591.1 (3.75) | 647.9 (3.67) |

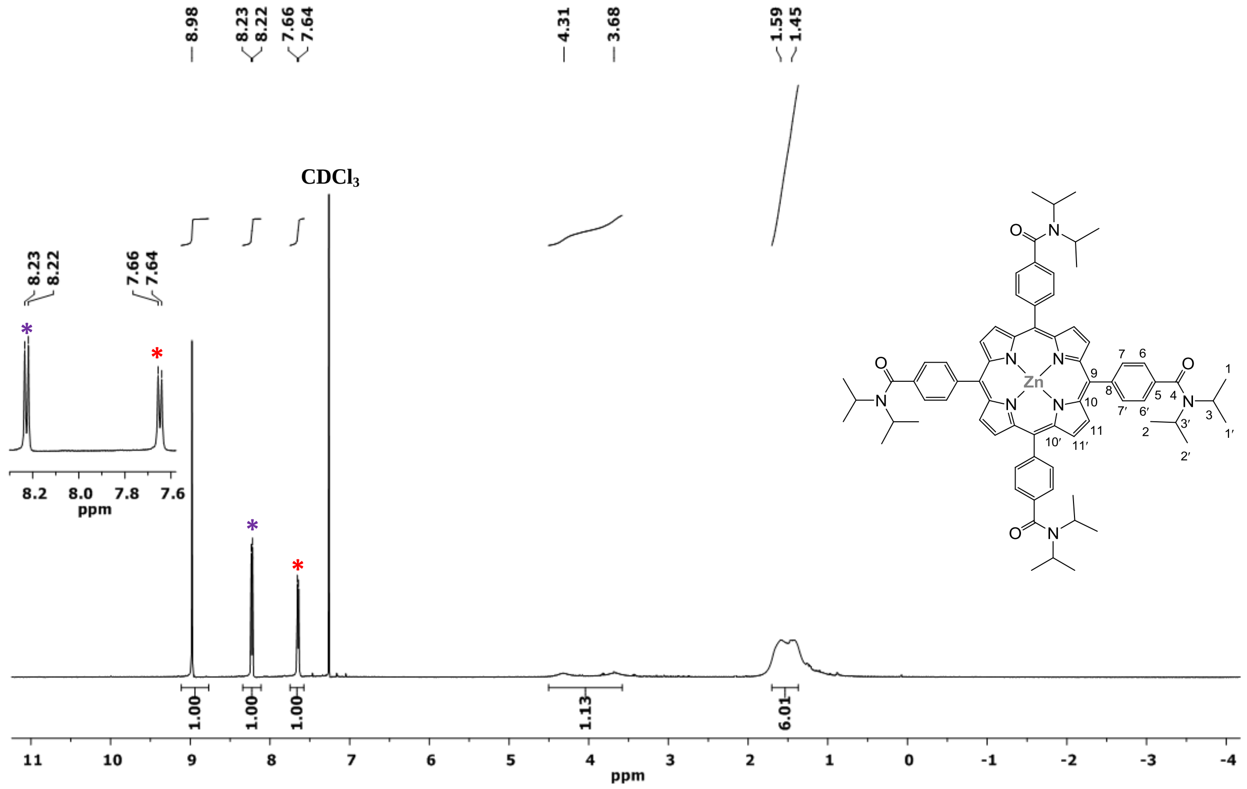


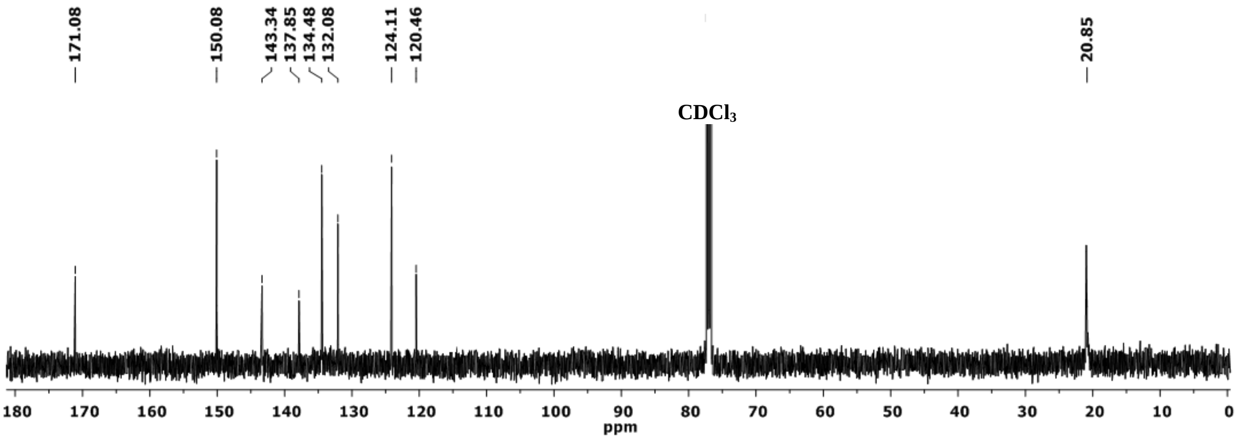


**Figure S23:** $^{1}$H (above) and $^{13}$C{$^{1}$H} NMR spectra (below) of **3a** in $CDCl_3$.

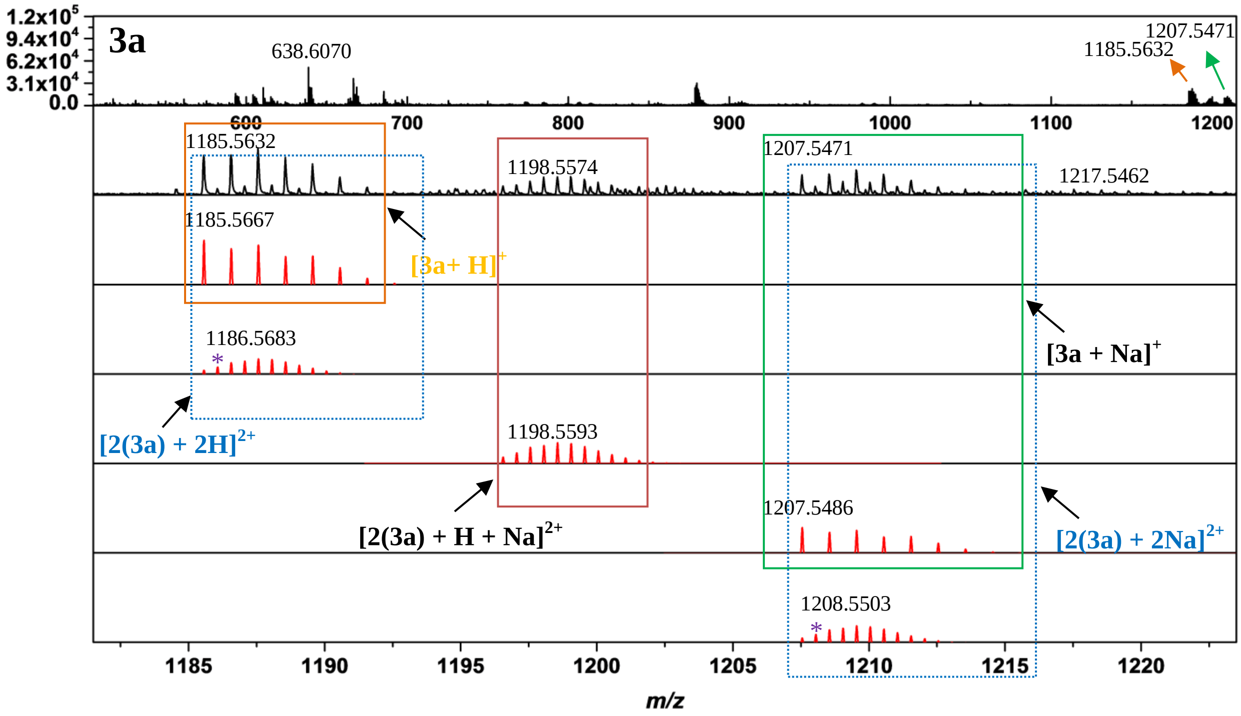


**Figure S24:** ESI-MS spectrum of **3a** (Black: Measured. Red: Calculated).

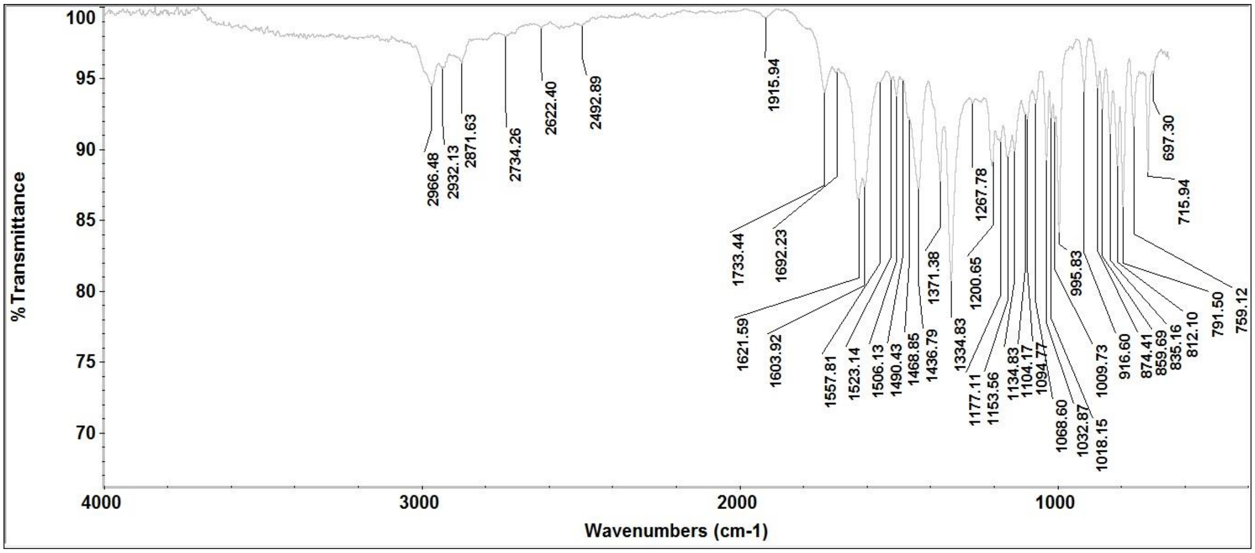


**Figure S25a:** IR spectrum (ATR-IR) of **3a**.

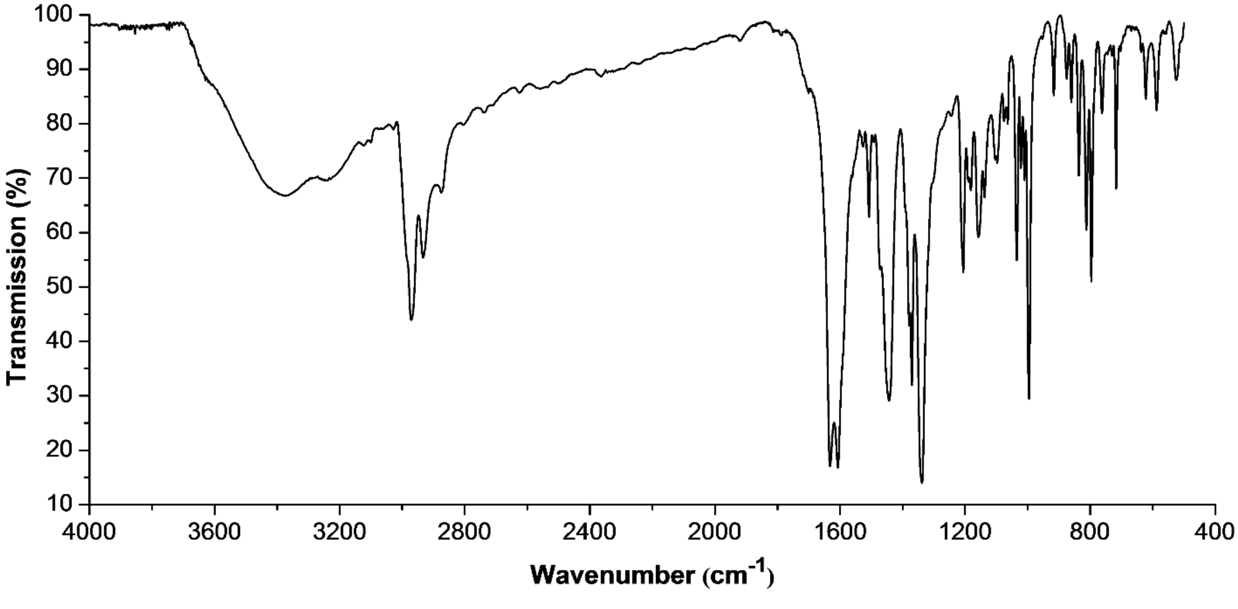


**Figure S25b:** IR spectrum (KBr) of **3a**.

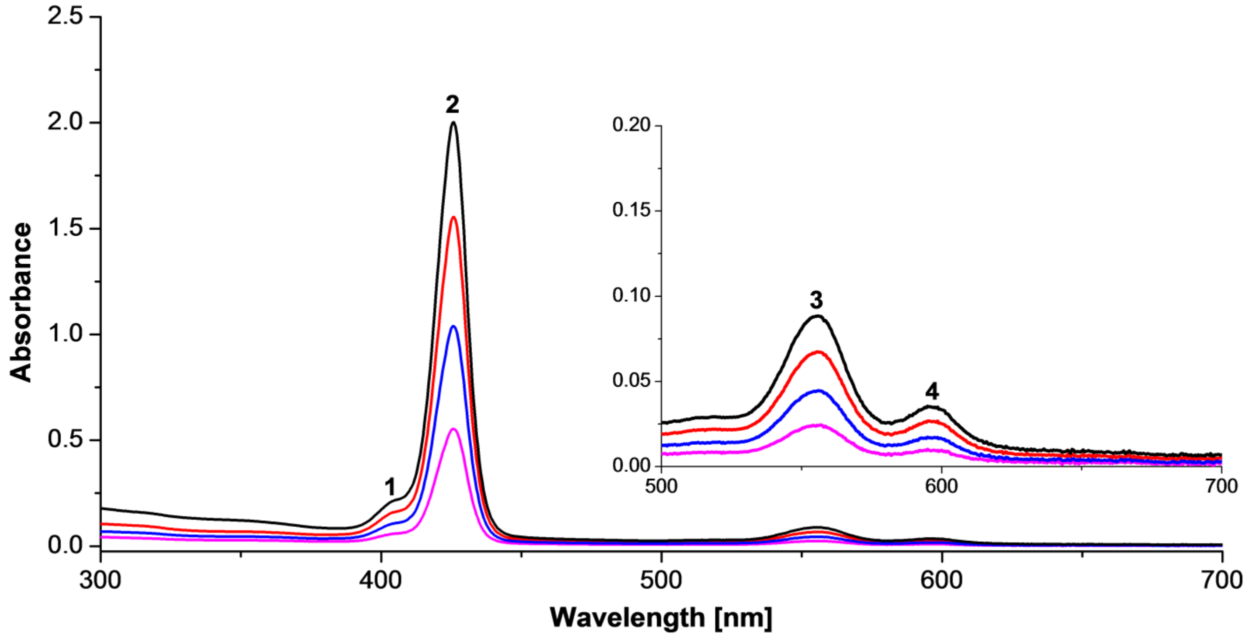


**Figure S26:** UV–vis spectrum of **3a** in $CHCl_3$ at different concentrations.

**Table S7:** UV–vis data ($\lambda_{max}$ [nm] log (ε [L·mol$^{-1}$·cm$^{-1}$])) of **3a** at different concentrations.

| | **Absorption $\lambda_{max}$ [nm] log (ε [L·mol$^{-1}$·cm$^{-1}$])** | | | |
|---|---|---|---|---|
| | **1** | **2** | **3** | **4** |
| ***C*₁ = 9.504·10$^{-6}$ mol/L** | 404.3 (4.36) | 425.9 (5.32) | 555.8 (3.97) | 596.7 (3.57) |
| ***C*₂ = 7.920·10$^{-6}$ mol/L** | 404.3 (4.33) | 425.9 (5.29) | 555.8 (3.93) | 596.7 (3.53) |
| ***C*₃ = 6.336·10$^{-6}$ mol/L** | 403.6 (4.26) | 425.9 (5.22) | 555.8 (3.85) | 596.7 (3.45) |
| ***C*₄ = 1.584·10$^{-6}$ mol/L** | 403.6 (4.57) | 425.9 (5.55) | 555.8 (4.19) | 596.7 (3.78) |

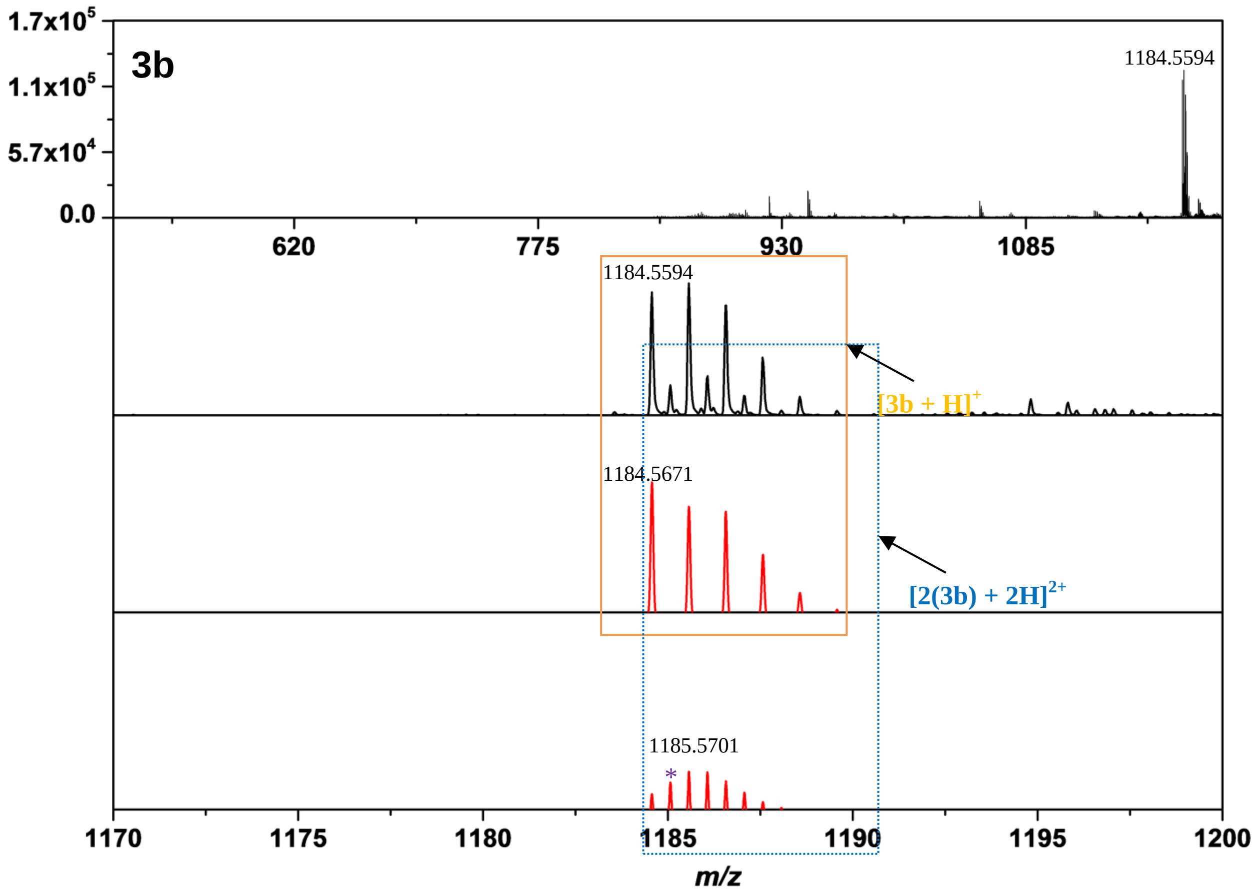


**Figure S27:** ESI-MS spectrum of **3b**. (Black: Measured. Red: Calculated).

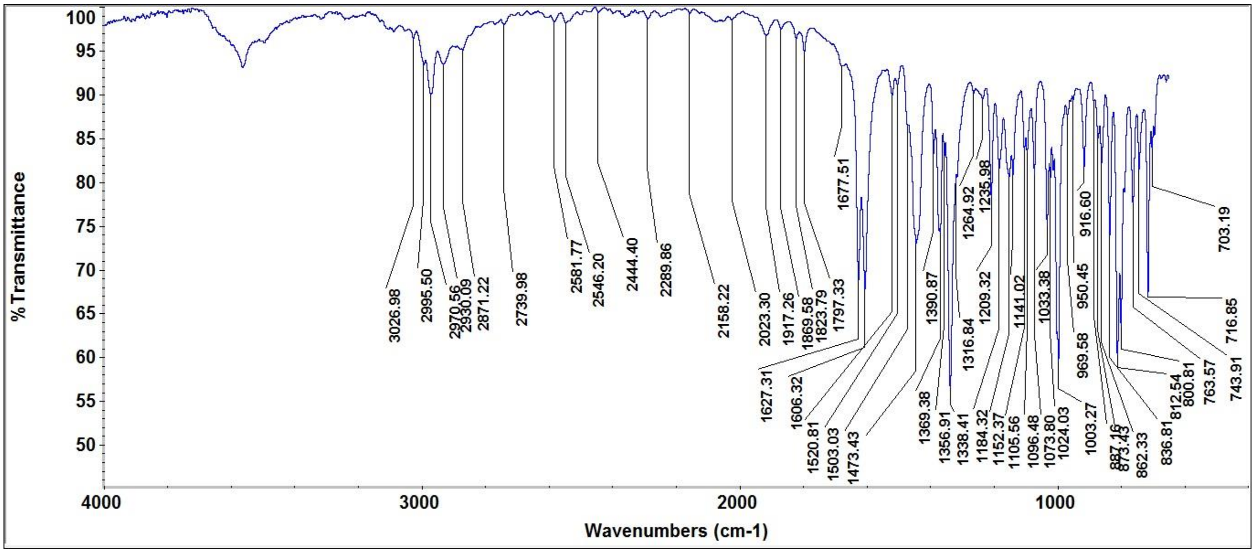

**Figure S28a:** IR spectrum (ATR-IR) of **3b**.

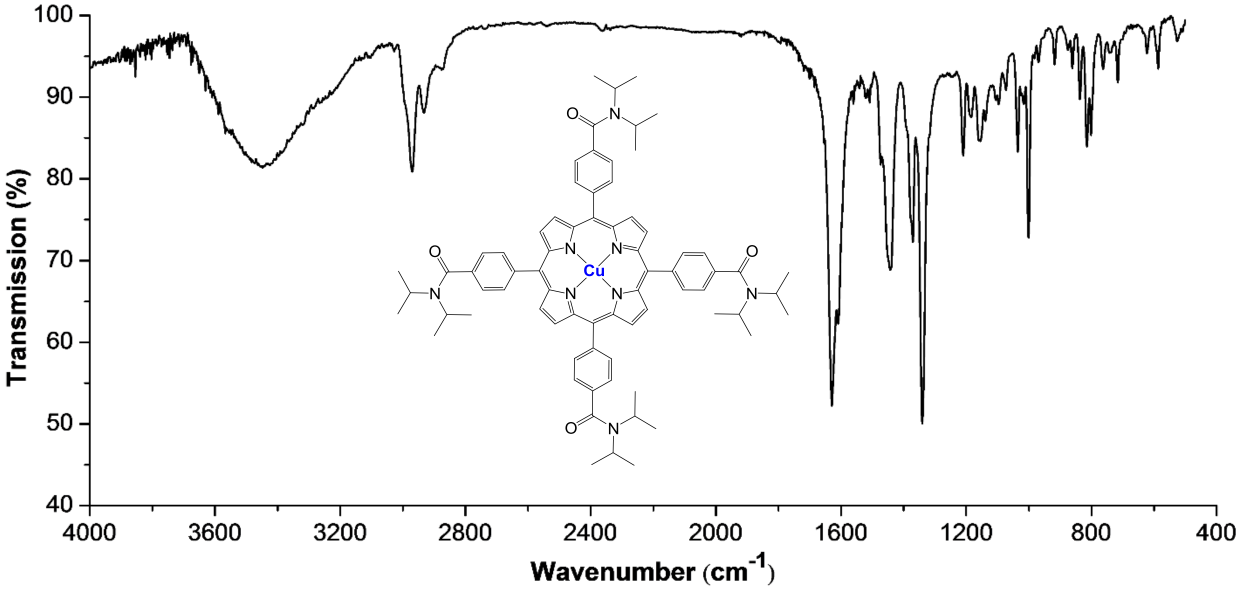

**Figure S28b:** IR spectrum (KBr) of **3b**.

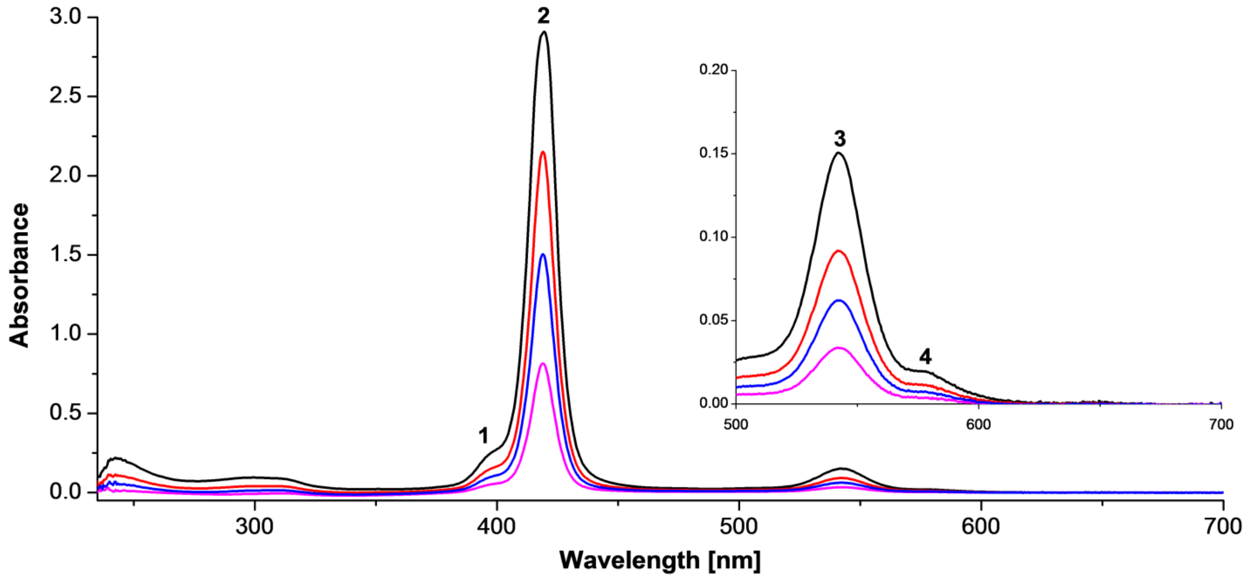


**Figure S29:** UV–vis spectrum of **3b** in $CHCl_3$ at different concentrations.

**Table S8:** UV–vis data ($\lambda_{max}$ [nm] log (ε [L·mol$^{-1}$·cm$^{-1}$])) of **3b** at different concentrations.

| | **Absorption $\lambda_{max}$ [nm] log (ε [L·mol$^{-1}$·cm$^{-1}$])** | | | |
|---|---|---|---|---|
| | **1** | **2** | **3** | **4** |
| ***$C_1$* = 5.401·10$^{-6}$ mol/L** | 397.3 (4.61) | 419 (5.69) | 542.4 (4.36) | 579.1 (3.44) |
| ***$C_2$* = 4.051·10$^{-6}$ mol/L** | 397.3 (4.58) | 419 (5.72) | 542.4 (4.36) | 579.1 (3.46) |
| ***$C_3$* = 2.700·10$^{-6}$ mol/L** | 397.3 (4.58) | 419 (5.74) | 542.4 (4.37) | 579.1 (3.43) |
| ***$C_4$* = 1.350·10$^{-6}$ mol/L** | 397.3 (4.58) | 419 (5.78) | 542.4 (4.39) | 579.8 (3.46) |

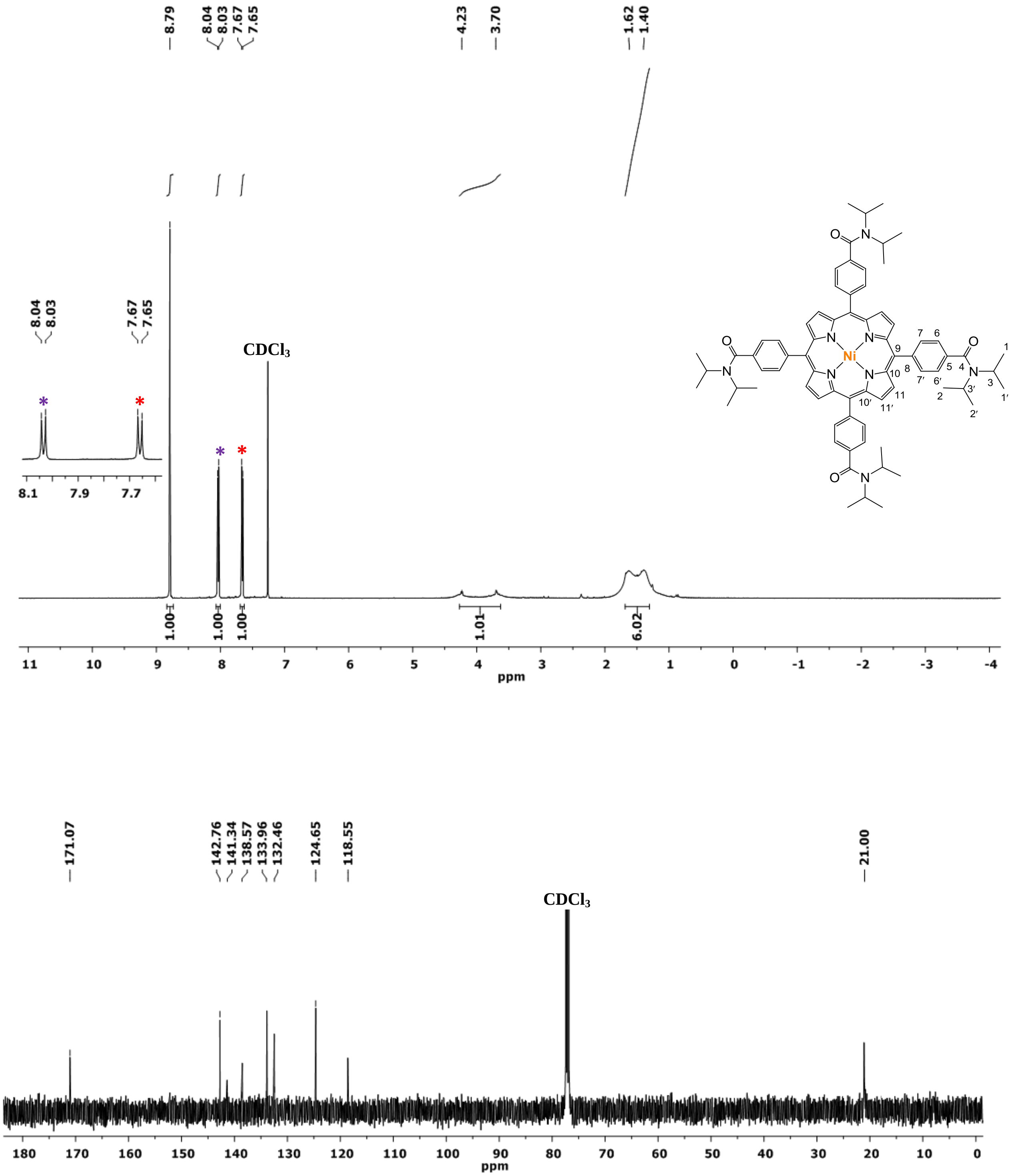


**Figure S30:** $^{1}$H (above) and $^{13}$C{$^{1}$H} NMR spectra (below) of **3c**.

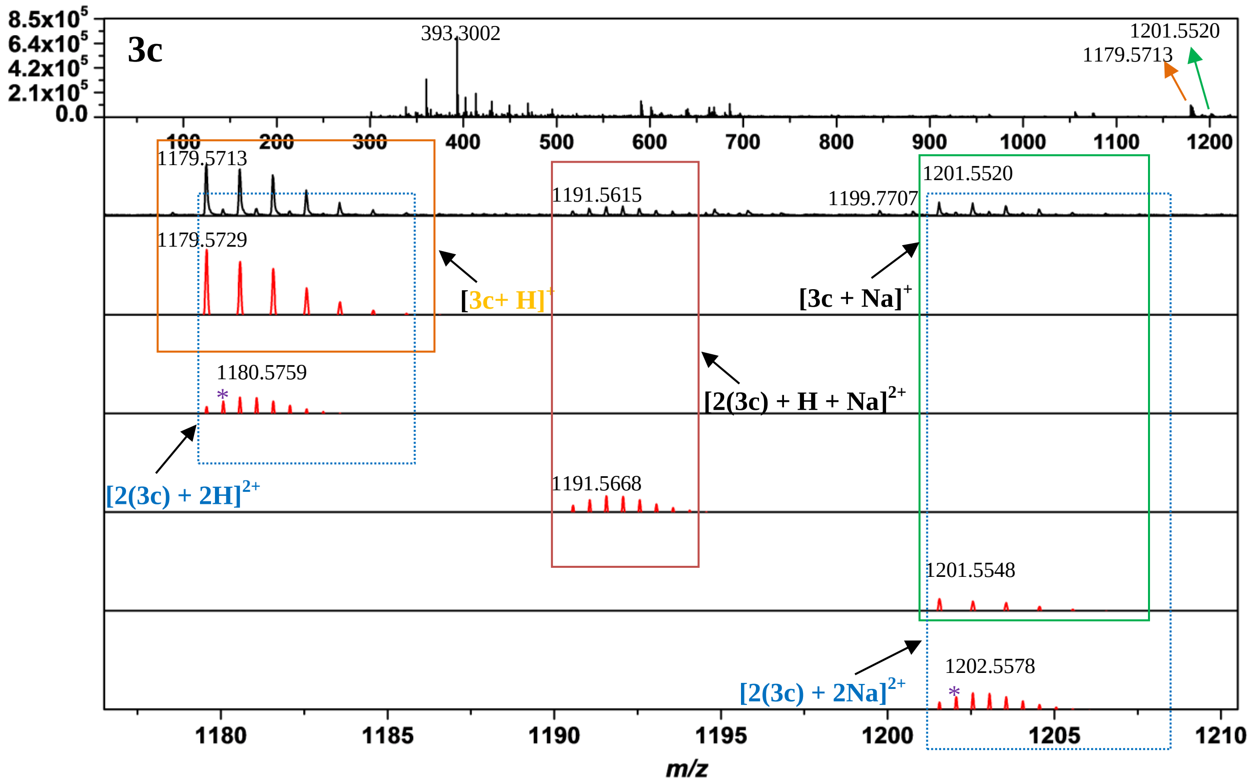


**Figure S31:** ESI-MS spectrum of **3c** (Black: Measured. Red: Calculated).

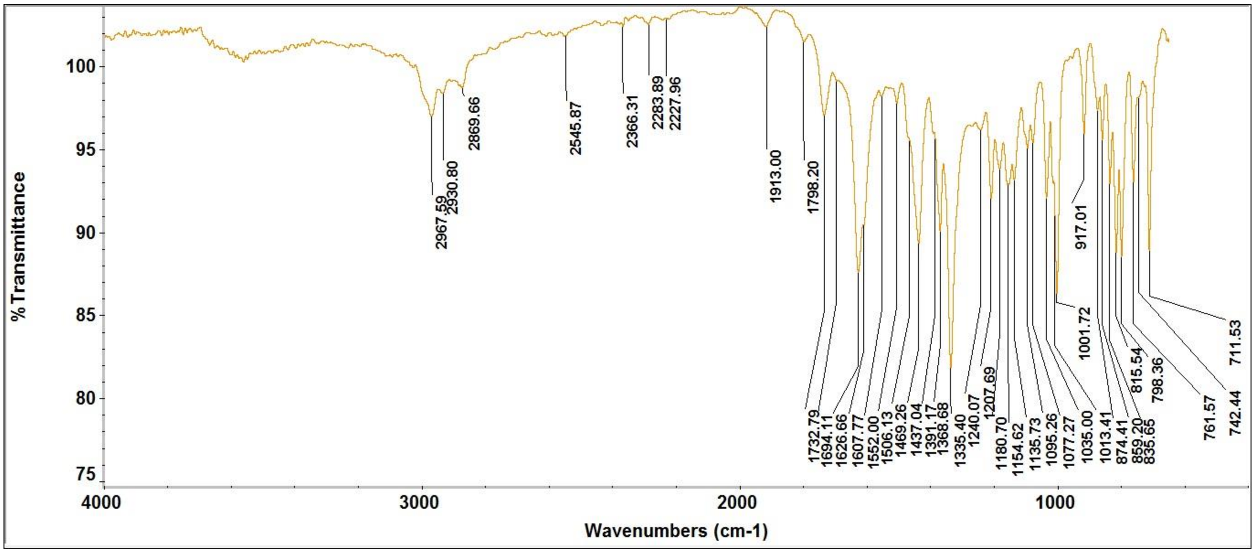


**Figure S32a:** IR spectrum (ATR-IR) of **3c**.

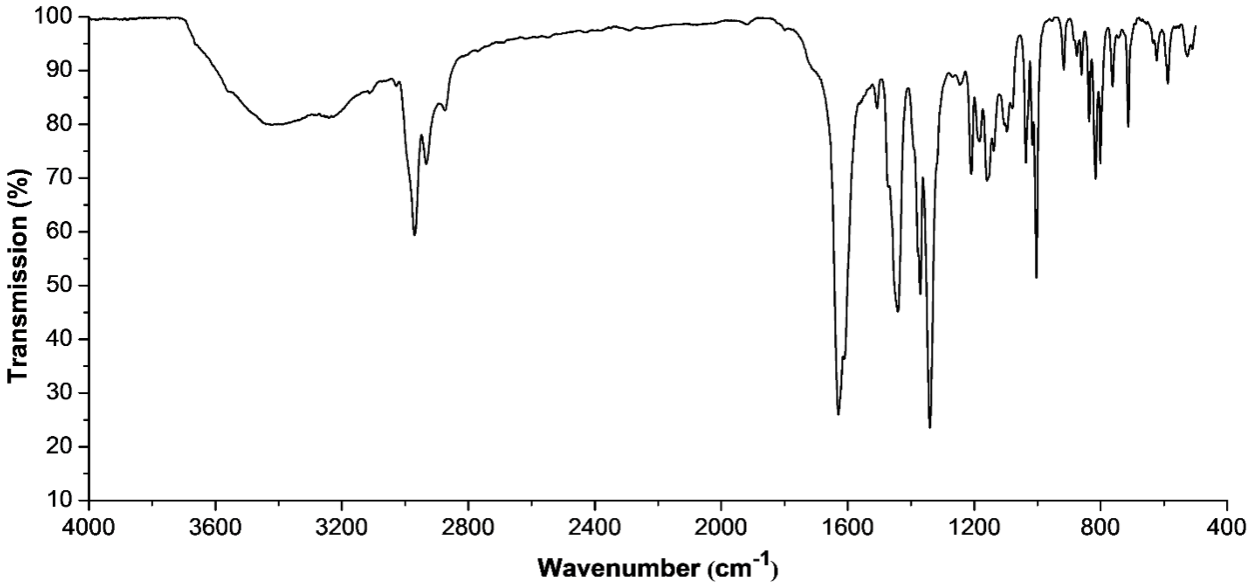


**Figure S32b:** IR spectrum (KBr) of **3c**.

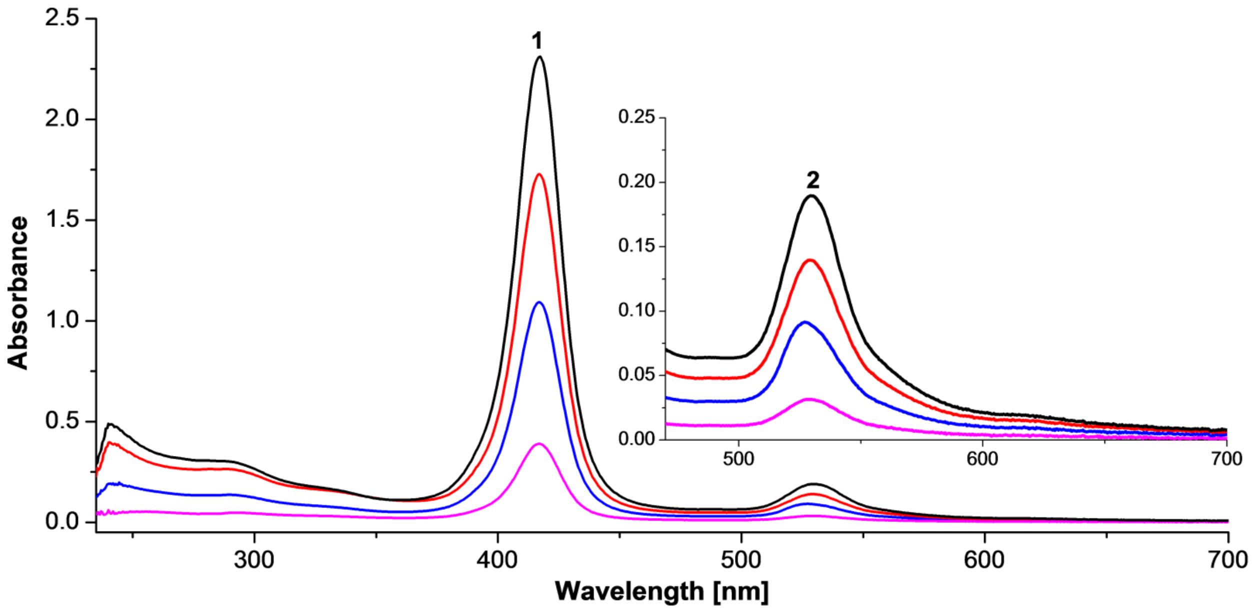


**Figure S33:** UV–vis spectrum of **3c** in $CHCl_3$ at different concentrations.

**Table S9:** UV–vis data ($\lambda_{max}$ [nm] log (ε [$L{\cdot}mol^{-1}{\cdot}cm^{-1}$])) of **3c** at different concentrations.

| | **Absorption $\lambda_{max}$ [nm] log (ε [$L{\cdot}mol^{-1}{\cdot}cm^{-1}$])** | |
|---|---|---|
| | **1** | **2** |
| $C_1 = 1.151{\cdot}10^{-5}$ **mol/L** | 417.2<br>(5.30) | 529.8<br>(4.22) |
| $C_2 = 8.219{\cdot}10^{-6}$ **mol/L** | 417.2<br>(5.32) | 529.5<br>(4.23) |
| $C_3 = 4.932{\cdot}10^{-6}$ **mol/L** | 417.2<br>(5.35) | 527.2<br>(4.27) |
| $C_4 = 1.644{\cdot}10^{-6}$ **mol/L** | 417.2<br>(5.38) | 529.5<br>(4.29) |

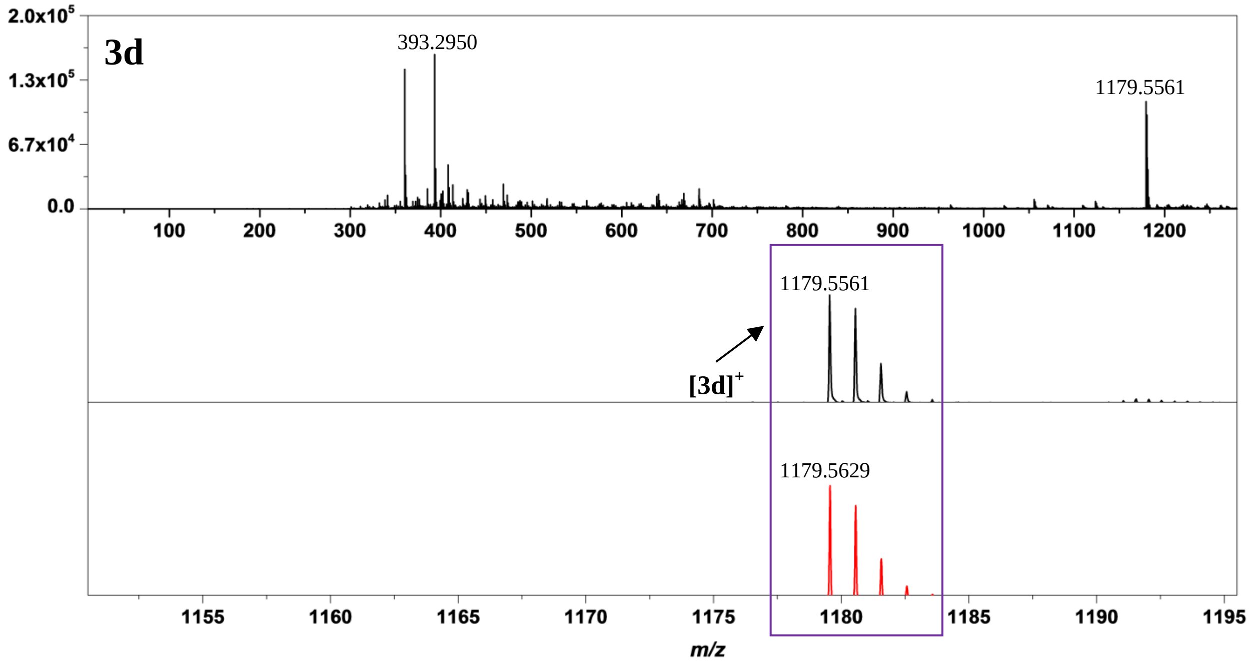


**Figure S34:** ESI-MS spectrum of **3d** (Black: Measured. Red: Calculated).

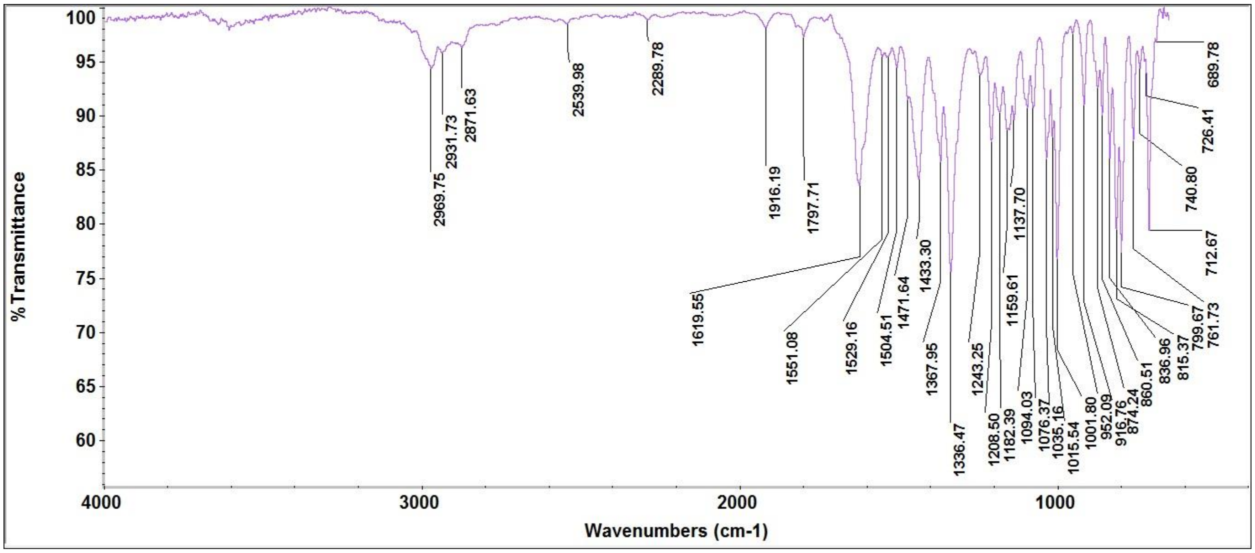


**Figure S35a:** IR spectrum (ATR-IR) of **3d**.

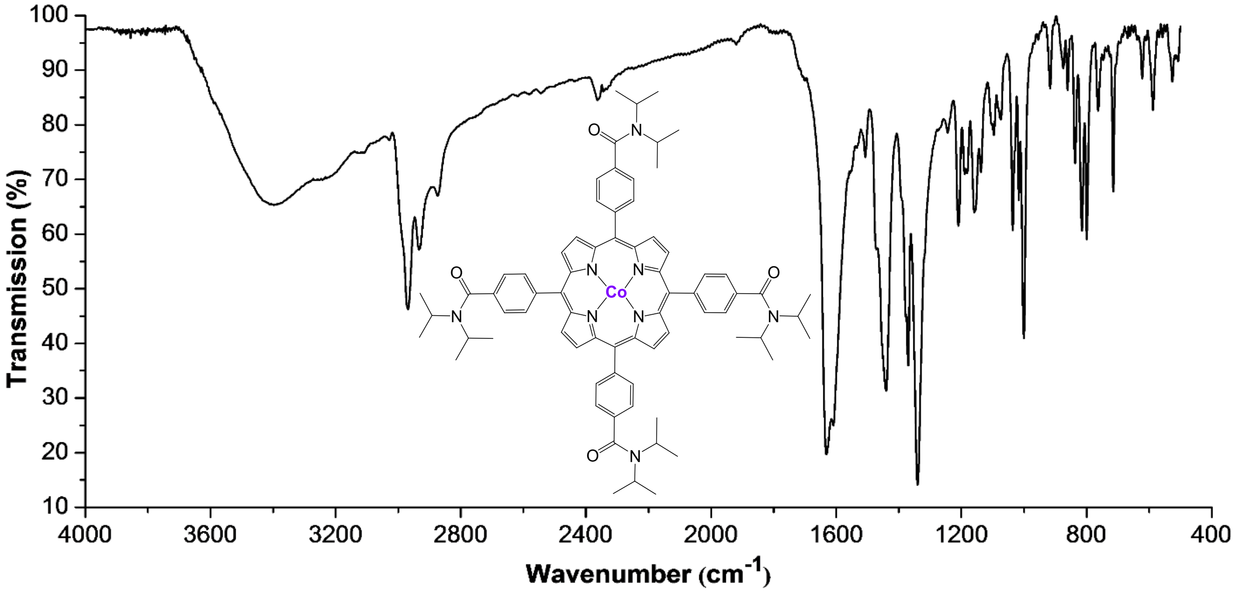


**Figure S35b:** IR spectrum (KBr) of **3d**.

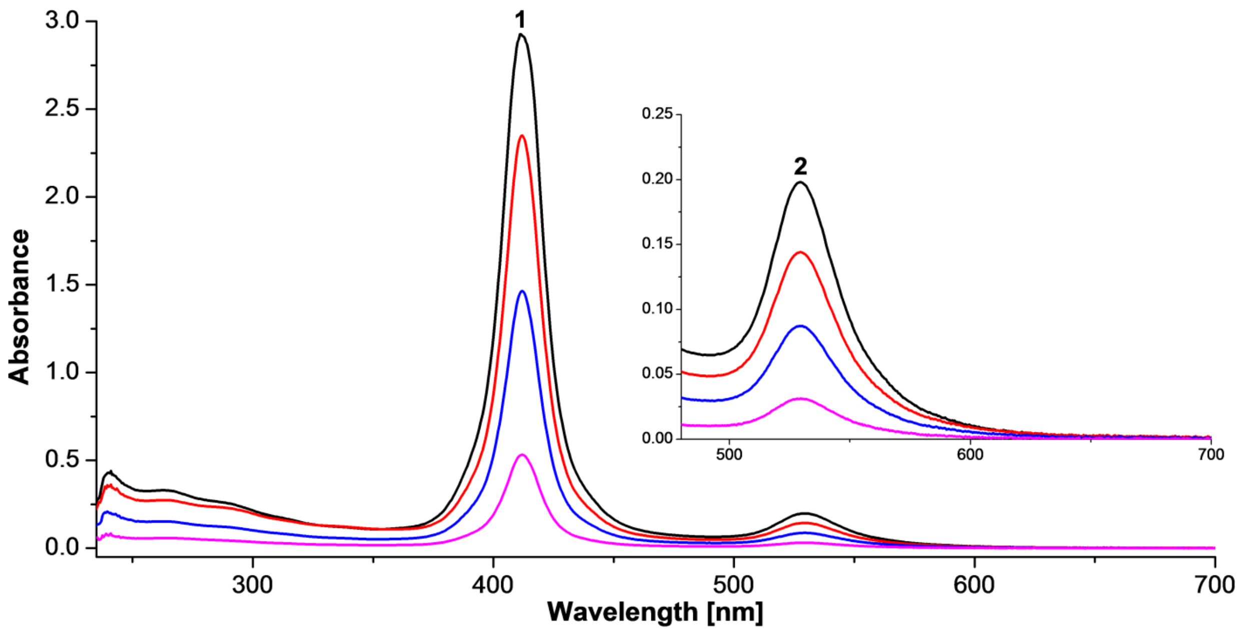


**Figure S36:** UV–vis spectrum of **3d** in $CHCl_3$ at different concentrations.

**Table S10**: UV–vis data ($\lambda_{max}$ [nm] log (ε [L·mol$^{-1}$·cm$^{-1}$])) of **3d** at different concentrations.

| | **Absorption $\lambda_{max}$ [nm] log (ε [L·mol$^{-1}$·cm$^{-1}$])** | |
|---|---|---|
| | **1** | **2** |
| **$C_1$ = 1.148·10$^{-5}$ mol/L** | 412.3<br>(5.41) | 529.4<br>(4.10) |
| **$C_2$ = 8.203·10$^{-6}$ mol/L** | 412.3<br>(5.45) | 529.4<br>(4.24) |
| **$C_3$ = 4.922·10$^{-6}$ mol/L** | 412.3<br>(5.47) | 529.4<br>(4.25) |
| **$C_4$ = 1.641·10$^{-6}$ mol/L** | 412.3<br>(5.51) | 529.4<br>(4.28) |

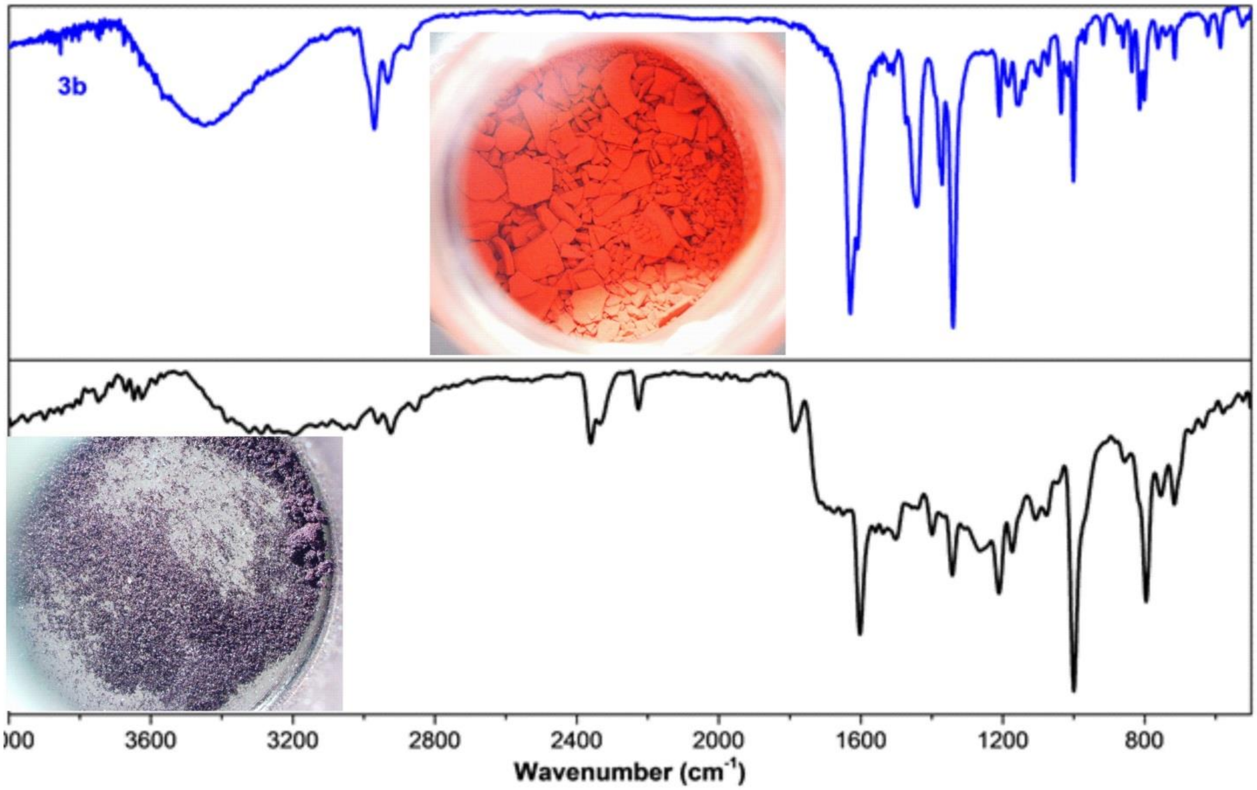


**Figure S37:** IR spectra (KBr) of **3b** (above) and in comparison to remains of **3b** after OMBD trials (below). Optical photographs of the material before (red) and after (purple) OMBD.

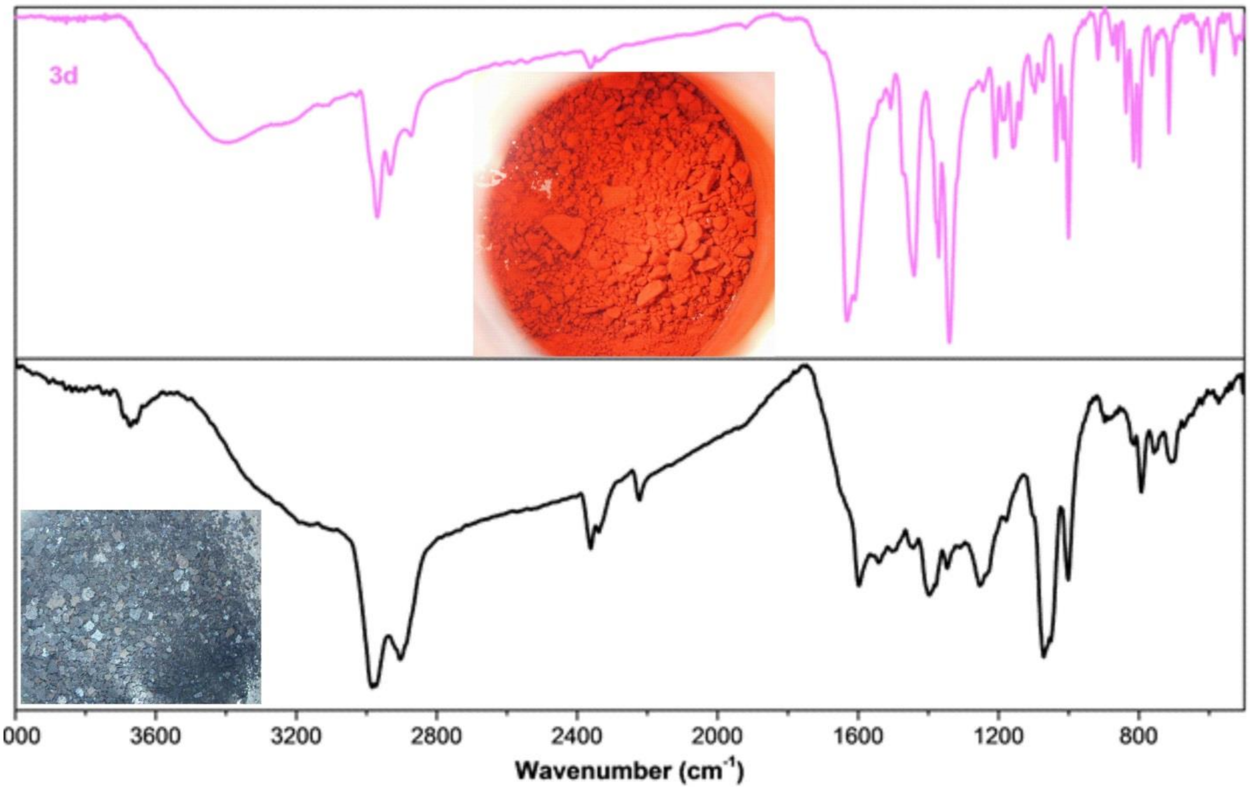


**Figure S38:** IR spectra (KBr) of **3d** (above) and in comparison to remains of **3d** after OMBD trials (below). Optical photographs of the material before (red) and after (purple) OMBD.

## References

S1 Jones, R. G.; Wilkins, J. M. *Organic Magnetic Resonance*. **1978**, *11*, 20–26. doi:10.1002/mrc.1270110102

S2 Manke, A. M.; Geisel K.; Fetzer, A.; Kurz, P. *Phys. Chem. Chem. Phys*. **2014**, *16*, 12029–12042. doi:10.1039/c3cp55023k
